# Antimicrobial Resistance and Associated Genetic Determinants of *Aliivibrio* Colonizing the Gastrointestinal Tract of Farmed Atlantic Salmon (*Salmo salar* L.)

**DOI:** 10.3390/antibiotics15090888

**Published:** 2026-09-10

**Authors:** Josephine K. Hamlett, John P. Bowman

**Affiliations:** Tasmanian Institute of Agriculture, University of Tasmania, Launceston, TAS 7250, Australia

**Keywords:** aquaculture, functional gene prediction, plasmids, integrons, Vibrionaceae, gut microbiome

## Abstract

Background/Objectives: Currently, readily available data on antimicrobial resistance in bacteria colonizing farmed Atlantic salmon (*Salmo salar* L.) are limited. Recent data indicate that the gastrointestinal tract mucosa of adult Atlantic salmon farmed in Tasmania (Australia) is consistently colonized by *Aliivibrio* species. *Aliivibrio* and other Vibrionaceae may contribute to gut dysbiosis through overgrowth. We investigated *Aliivibrio* isolates and other *Aliivibrio* species to link antimicrobial resistance (AMR) phenotypes with the presence of antimicrobial resistance genes (ARGs). Methods: We performed antimicrobial susceptibility testing on Atlantic salmon bacterial isolates (n = 50). We surveyed ARGs in representative genome-sequenced strains (n = 21) and across the genus *Aliivibrio* (n = 112 strains) using a range of bioinformatic approaches. Results: Salmon *Aliivibrio* isolates had high MIC values for multiple antimicrobial drug classes (penams, tetracyclines, sulfonamides, macrolides, and aminoglycosides), including antimicrobials important for aquaculture prophylaxis. The greatest susceptibility was observed for chloramphenicol, oxolinic acid, ciprofloxacin, rifampicin, meropenem, trimethoprim, and trimethoprim–sulfamethoxazole. Provisional analysis suggests that the resistance profile of *Aliivibrio* isolates matches a core set of predicted ARGs common to the genus *Aliivibrio*. Among the isolates, plasmids and integrons did not include genes similar to known ARGs. Based on available genome data, strains of several *Aliivibrio* species carried predicted ARGs on integrons, including genes providing potential resistance to chloramphenicol (*cpt*, *catB*), tetracyclines (*tetE*), sulfonamides (*sul2*), and trimethoprim (*dfrA1*). Conclusions: Based on these results, we estimated provisional epidemiological cutoff values for several antimicrobials for *Aliivibrio* isolates predominant in Atlantic salmon farmed in Tasmania. Furthermore, available data show no evidence that plasmid- or integron-associated ARGs are prevalent and suggest that they are, overall, uncommon in the genus *Aliivibrio*. The data presented provide a foundation for monitoring AMR in Atlantic salmon gut-associated commensal bacteria.

## 1. Introduction

Studies worldwide show ARGs circulating in marine aquaculture systems, with this risk assessed from public health, One Health, and biosecurity management perspectives [1,2,3,4]. Despite advances in prophylaxis for farmed finfish—such as Atlantic salmon (*Salmo salar*)—including vaccines [5], improved farming practices [6,7], and innovative diet-based strategies [8,9,10], antimicrobial agents remain a key lever for bacterial disease management [11]. These approaches have helped reduce reliance on antimicrobials [6,12].

Gene prediction suggests that ARGs for most drug classes may be enriched at Atlantic salmon farming sites [13], while known pathogens may also be enriched in waters surrounding fish farms [14,15]. In salmon farming regions, summer conditions seem to favor the growth of a broader diversity of Vibrionaceae and other fast-growing bacteria in surface waters [16,17]. Though Atlantic salmon are vaccinated against invasive pathogenic species (e.g., *Vibrio anguillarum*, *Aliivibrio salmonicida*, and *Aeromonas salmonicida*), several bacterial species still function as potentially pathogenic or dysbiotic colonizers of the fish gut microbiome or can infect wounds and healthy tissue in the marine phase of farming (e.g., *Aliivibrio* spp., *Moritella viscosa*, and *Tenacibaculum* spp.). ARG spread may therefore increase during summer, especially in thermally stressed fish, and AMR bacteria may occur naturally within fish microbiomes [18].

In a study using a salmon gut simulation model, the florfenicol resistance gene *floR* spread to diverse bacteria via a promiscuous plasmid [19]. This included *Aliivibrio wodanis*, a co-pathogen of Atlantic salmon alongside *Moritella viscosa* [20]. FloR was also found to persist in some non-Vibrionaceae species [19]. This suggests that ARG repertoires could build up in Atlantic salmon gut colonizers on mobile genetic elements (MGEs). With good stewardship, ARG accumulation should, in theory, be unlikely and, if it occurs, localized. Nevertheless, fish farm locations could be affected by ARG inputs from external sources (e.g., [21]), potentially enhanced by climate change and eutrophication.

Australian finfish producers have very limited access to antimicrobials, with none registered for routine use. Usage requires specific permits or direct veterinarian-instigated administration [22]. In the case of Atlantic salmon (*Salmo salar*) farmed in the south-east coastal regions of Tasmania, a large outbreak of piscirickettsiosis in 2025 resulted in allowance of use of florfenicol; however, detection of this drug in wild fish in the local environment led to revocation of the permit by the Australian Pesticides and Veterinary Medicines Authority [23]. With limited therapeutic options, measures are needed to ensure the long-term efficacy of currently available antimicrobial agents. Comprehensive surveillance systems that measure resistance prevalence, identify potential threats, and track trends in antimicrobial resistance in aquatic bacterial species may help manage existing antimicrobial resistance (AMR) thresholds [24]. This is supported by gathering data on minimum inhibitory concentration (MIC) breakpoints and investigating the involvement or presence of ARGs that can facilitate AMR. Researchers have developed a range of comprehensive online and cloud-based resources for ARG detection [25].

In the Tasmanian Atlantic growing region, evidence of ARG accumulation in salmon-associated bacteria remains unknown. Detailed knowledge of bacterial gut colonizers of farmed Atlantic salmon has only recently been defined. Multi-year surveys of Tasmanian salmon have shown that mostly *Aliivibrio* spp. peak in population in the gut microbiome 7 to 9 months following the transfer of smolts to coastal farm sites, essentially after the conclusion of the first summer season on the farm [26]. Other bacteria showing consistent colonizing trends include *Vibrio scophthalmi* group strains, *Photobacterium piscicola*, and an unclassified Mycoplasmoidaceae. Because *Aliivibrio* consistently outnumbers other bacteria in salmon gut microbiomes, this investigation focused on *Aliivibrio* AMR to better understand its relationship to ARGs and MGEs.

Currently, published data on AMR of the genus *Aliivibrio* is practically non-existent. To establish clinical susceptibility data, we need to explicitly determine antimicrobial susceptibility ranges using CLSI (Clinical and Laboratory Standards Institute) clinical breakpoints and define the presence of ARGs that may influence breakpoints [27]. Here, we performed MIC and disk diffusion assays for several antimicrobials, particularly drugs used for finfish prophylaxis, on 50 *Aliivibrio* isolates. This approach is limited because no breakpoint data are available for any *Aliivibrio* species; therefore, intrinsic versus acquired resistance can only be defined provisionally, and the results will not be suitable for clinical applications. To advance this study, we also aimed to define MIC-based Epidemiological Cutoff (ECOFF) values to estimate expected antimicrobial tolerance ranges for the salmon *Aliivibrio* isolates. We also determined provisional susceptibility by comparing data from human-pathogenic members of the genus *Vibrio*, the closest relatives to *Aliivibrio*, using the available CLSI criteria and literature. Because knowledge of target genus-specific ARGs was limited, PCR detection was not considered feasible or informative. Instead, we attempted to link AMR phenotypes to genotype data using genome-based analysis [27]. We also assessed the association of putative ARGs with MGEs, including plasmids and integrons, to determine whether isolates had acquired laterally transferred ARGs.

## 2. Results

### 2.1. Atlantic Salmon Aliivibrio Isolates Surveyed

Of the 50 strains tested, 21 had been previously selected for genome sequencing to study genes associated with gut colonization [26]. This selection was firstly based on distinct 16S rRNA sequence groups amongst the strains. In this respect, three distinct groups occurred. Two strain clusters, containing six and four strains respectively, include the distinct species *Aliivibrio* sp028765545 and *Aliivibrio* sp028765575. Of the remaining 40 strains, 13 strains were selected based on REP-PCR profiles [28]. This profiling revealed three distinct band groups, from which we randomly selected representatives for sequencing. Group 1 included strains A2, A4, A33; group 2 contained A6, A9, A15, A21, A26, A37, and A46; and group 3 included A32, S2TY2, and S3TY1 (more details in the Supplementary Data). These groups differ as revealed by ANI analysis and in silico DNA:DNA hybridization [26]. The three groups belong to the same species (ANI ≥ 96.1%, [26]), designated *Aliivibrio finisterrensis_A* based on the GTDB framework. The 40 strains tested included 7, 28, and 5 group 1, 2, and 3 strains, respectively.

### 2.2. MIC-Based Resistance Profiles of Aliivibrio Isolates

Table 1 shows MIC50 and MIC90 values, the overall MIC range, and estimated ECOFF values for the three *Aliivibrio* species groups. Susceptibility was provisionally estimated based on responses typical of *Vibrio* spp., to which *Aliivibrio* are most closely related (see Section 4.2). Using these presumptive breakpoints, 88 to 100% of salmon *Aliivibrio* isolates were susceptible to chloramphenicol, oxolinic acid, ciprofloxacin, meropenem, rifampicin, and trimethoprim. *Aliivibrio* isolates had much higher MICs for the other antimicrobials, including various penams, tylosin, florfenicol, and all of the aminoglycosides evaluated. Tetracycline and oxytetracycline resistance was found to be lower in *A.* sp028765575 strains compared to the other isolates (*A. finisterrensis_A*. *A.* sp028765545) (Table 1).

### 2.3. Disk Diffusion Susceptibility Profiles of Aliivibrio Isolates

Based on presumptive breakpoints (Section 4.2), the *Aliivibrio* isolates demonstrated apparent resistance to ampicillin (10 μg), benzylpenicillin (1 IU), and streptomycin (10 μg) (Table 2). A greater range of responses occurred with tetracycline (25 μg), with 4 out of 50 strains sensitive (all belonging to *Aliivibrio* sp028765575, zone radius 20–21 mm), with the remaining strains having intermediate or higher levels of resistance (zones 0 to 12 mm). All the isolates were susceptible to chloramphenicol (25 μg), cefepime (10 μg), and ceftriaxone (10 μg). Most (76%) of the isolates were also sensitive to trimethoprim–sulfamethoxazole (1.25/23.75 μg) or had marginal intermediate resistance (12/50 strains, zones 14–15 mm). In comparison, resistance (38% of strains) was more prevalent against sulfatriad (200 μg).

### 2.4. Aliivibrio ARG Core Set

**Figure 1:**
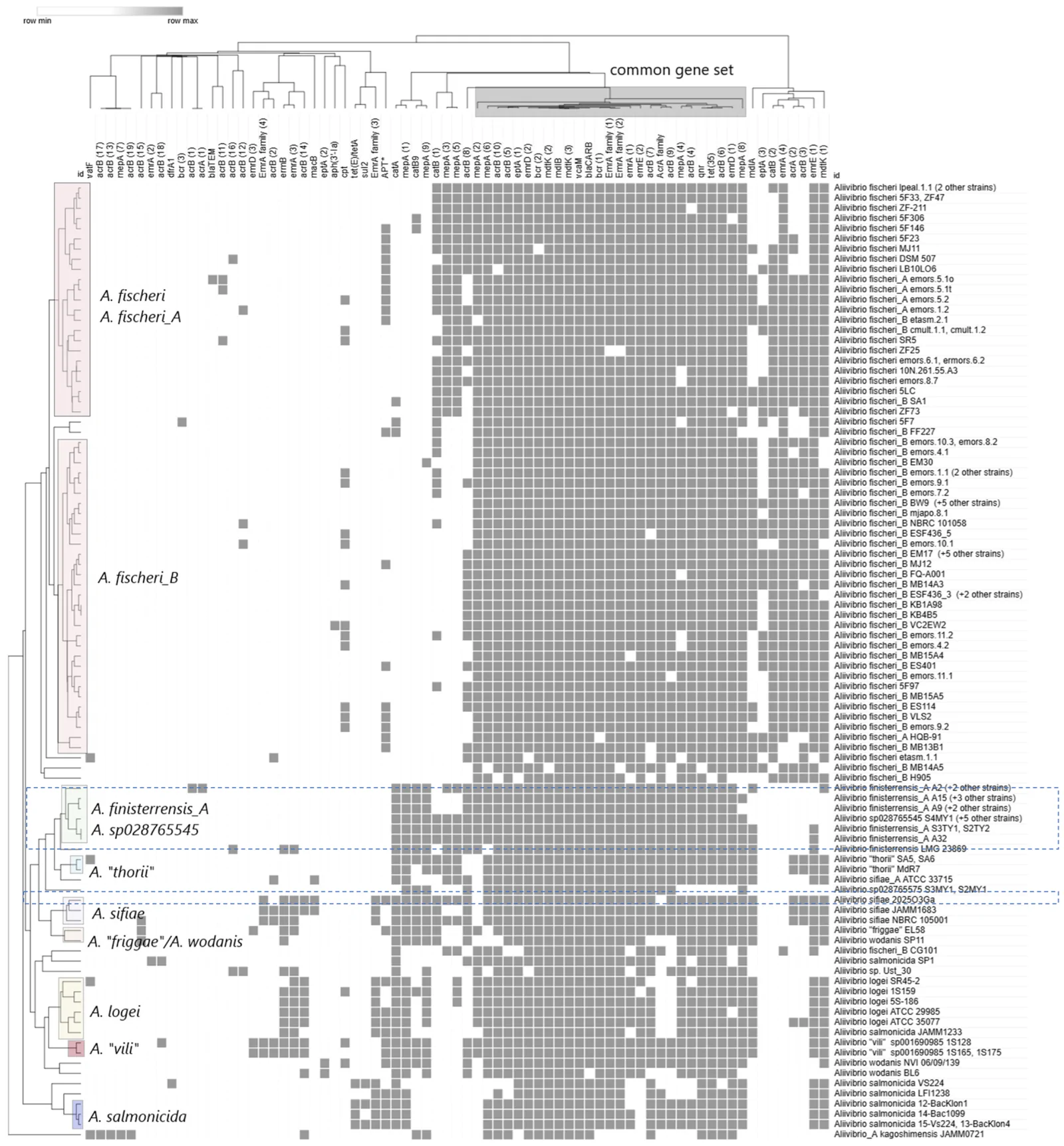
Weighted presence/absence heat map (generated using Morpheus, Broad Institute) showing the distribution of putative ARGs in *Aliivibrio* genomes. *Aliivibrio* species strains forming common SIMPROF groups are denoted by colored boxes. A common gene set for all strains is denoted by the gray box among the gene clusters. We clustered ARGs and strains using Euclidean distances and complete linkage. SIMPROF analysis used α = 0.05. For strains, we show only those with a distinct ARG pattern. The number of strains with identical patterns is indicated in parentheses or listed in the same row. APT* indicates a gene with an aminoglycoside phosphotransferase domain.

**Figure 2:**
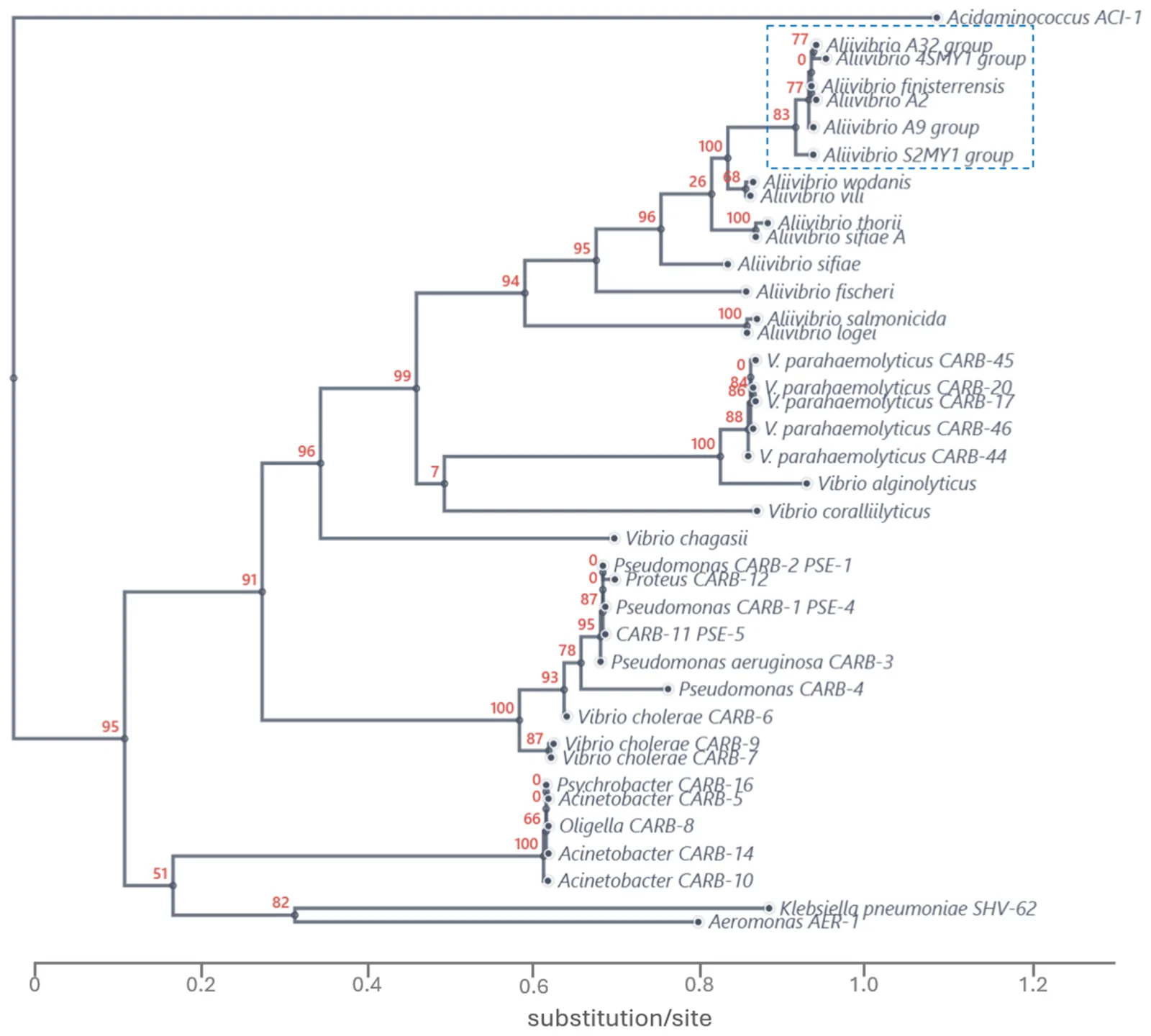
Phylogenetic tree showing the relationship of putative type A beta-lactamases/carbenicillinases from *Aliivibrio* species with authenticated CARB-type lactamases from *Vibrio* spp. and other members of class Gammaproteobacteria. Atlantic salmon isolate homologs are confined to the blue-checked box. Red numbers are percent bootstrap probabilities. The outgroup is the ACI-1 lactamase from *Acidaminococcus fermentans* (class Negativicutes, phylum Bacillota).

From the database surveys, we identified putative ARGs and compared them in a clustered heat map (Figure 1). Supplementary Data show functional predictions for the ARGs and their relation to KEGG orthology. Clustering and SIMPROF analysis revealed a suite of ARGs common across the genus *Aliivibrio*. The most common resistance mechanisms were efflux pumps, with paralogous genes coding for multiple drug/toxic substance transporter protein families. In these cases, the gene label is annotated with a number to indicate distinct paralogous gene clusters (Figure 1). Efflux pump protein-coding genes were found in >90% of *Aliivibrio* strains. They included the transporter protein families Tet(35), MdlB-like/Tet(60), VcaM, MdtK, Bcr, EmrA, EmrD, EmrE, Qnr, ArcA/ArcB, and MepA. The core ARG set included a *bla_CARB_* (carbenicillinase) gene. Bla_CARB_ proteins form a cluster (similarity > 64%) closest to CARB-17 group A lactamases of *Vibrio parahaemolyticus* and related *Vibrio* species (similarity 50–55%) (Figure 2). All *Aliivibrio* also possess at least one *etpA* (ethanolamine phospholipid acyltransferase) homolog.

### 2.5. Salmon Aliivibrio Isolate ARG Distributions

Salmon isolate ARGs are shown in relation to other genome-sequenced strains of the genus *Aliivibrio* (Figure 1). *A. finisterrensis_A* and the S4MY1 group strains (*A.* sp028765545), the latter of which are positive for the genes for cytolethal distending toxin (CDT) [26], have a similar array of ARGs forming a common SIMPROF cluster. This common cluster groups closely with the *A. finisterrensis* type strain LMG 23869, *A. “thorii”* (common SIMPROF cluster), and *A. sifiae_A* ATCC 35077. These strains varied in ARGs mainly by the presence or absence of homologs of the efflux-related genes *mdtK*, *emrE*, *mdfA*, *bcr*, and *acrA/acrB*. All possess a homolog similar to the *catB9* gene, while some strains also have an additional *catB* gene (Figure S1). The second group of salmon strains included *A.* sp028765575, which clustered separately from other species. *A.* sp028765575 strains lacked *tet(35)* and *qnr* homologs and had fewer efflux pump genes, especially those encoding MepA family proteins.

### 2.6. Non-Core ARG Distribution Across Aliivibrio

Broadly, ARG distribution followed species lines based on clustering patterns and SIMPROF groups (Figure 1). However, many strains had distinct ARG arrays. *A. fischeri* strain genomes (n = 83), which cover three closely related ANI-defined groups (i.e., *A. fischeri* sensu stricto, *A. fischeri_A*, and *A. fischeri_B*), mostly grouped into two SIMPROF groups, with *A. fischeri* and *A. fischeri_A* present in one group and most of *A. fischeri_B* in the second. These strains had, in addition to the core *Aliivibrio* ARGs, homologs of *eptA*, *catB*, *mdlA*, *mepA*, and *acrAB*. Furthermore, 20% and 24% of A. fischeri genomes possessed predicted homologs of cpt (chloramphenicol 3-O-phosphotransferase) and a gene encoding a protein with an aminoglycoside phosphotransferase (APH) domain, respectively. The latter protein differs substantially from well-defined 2″ and 3″-site Aph proteins; thus, its resistance-related function is only a tentative prediction. For completeness, Figure 1 includes this gene, designated APT*. Putative homologs for *bla_TEM_*, *vatF* (virginiamycin/streptogramin acetyltransferase), and *aph(3′-Ia)* (aminoglycoside phosphotransferase) were found in individual strains isolated from squid light organs (strains emors.5.1o, etasm.1.1, and VC2EW2, respectively). It should be noted that strain etasm.1.1 was obtained in Tasmanian waters from the southern bobtail squid (*Euprymna tasmanica*). It was observed that *cpt, vatF,* and APT* are also present in specific strains of *A. “vili”*, *A. logei*, *A. salmonicida*, *A. sifiae*, and *A. wodanis*. These species, as well as *A. “thorii”* and *A. “friggae*”, also host additional paralogs of *ermA/ermB*, *acrA/acrB*, *ermD*, and *mdfA* depending on the strain (Figure 1).

### 2.7. ARG Detection in Plasmids of Genus Aliivibrio

A 167–179 kb plasmid, presumed to be conjugative due to the presence of a *tra* region was found in 20% of the *Aliivibrio* isolates from salmon (A9, A21, S2TY2, S3TY1, S3MY1) and also several other *Aliivibrio* species including *A. fischeri* (MJ11, BW9, MB11B1, MB13B3, emors.8.7), *A. wodanis* (NVI 06/09/139) and *A. “thorii”* (SA6) (Table S2). The plasmids share genetic similarity with ANI ranging from 77 to 98%. None of these plasmids carried identifiable ARGs. This was also true for other *Aliivibrio* species with plasmids, except for large plasmids found in certain *A. salmonicida* strains. Strain VS224 plasmid pRVS1-224 (size 285 kb) contains homologs of *tetE* (DHA1 family MFS transporter), *dfrA1* (trimethoprim-resistant dihydrofolate reductase), and *sul2* (sulfonamide-resistant dihydropteroate synthase type 2). Three other strains (15-Vs224, 13-BacKlon4, 12-BacKlon1, 14-Bac1099) possess 236 kb plasmids that are genetically similar to pRVS1 (98% ANI); *dfrA1*, *tetE*, and *sul2* homologs also occur on these plasmids (Table 3).

### 2.8. ARG Detection in Integrons of Genus Aliivibrio

Integrons were detected in almost all *Aliivibrio* strains (details shown in Supplementary Data, Tables S3 and S4) though in most strains they are degenerate, appearing as clusters of (≥2 sites) (≥2 sites) AttC sites in regions lacking integron integrases. These are designated as “CALIN” (Table 3). We did not investigate genetic regions with only one AttC site, as they indicate a nearly fully decayed integron and do not reliably define integron location. The largest integrons ranged from 62 to 75 kb, though most were much smaller (typically 2 to 10 kb). In the *Aliivibrio* isolates and in the large majority of other *Aliivibrio* integron regions, we did not find definitively identifiable ARGs. Intact and degenerate integrons with the homologs of *cpt*, *catB,* and APT* were found in genomes of some strains of *A. fischeri*, *A. sifiae*, *A. logei*, *A. salmonicida*, and *A. wodanis* (Table 3). Where this could be confirmed, integrons with ARGs were almost always located on chromosome 2, except in the case of plasmid-associated *dfrA1*, which was associated with 4.04 kb integrons present as three copies on pRVS1-224 of strain VS224, with one *dfrA1* allele being a pseudogene. Three strains (15-Vs224, 13-BacKlon4, 12-BacKlon1) have a single intact copy of the same 4.04 kb integron with *dfrA1* genes, while strain 14-Bac1099 has lost the integron. *Aliivibrio* integron regions in the isolates code mostly for hypothetical proteins, proteins with only generalized predicted functions, and are often toxin-antitoxin module-rich. We flag potentially interesting functional genes in the Supplementary Data.

## 3. Discussion

*Aliivibrio* are key members of the GI tract of farmed Atlantic salmon in Tasmania [29,30,31] and, depending on the temperature conditions, also occur in salmon of other production regions [26]. In Tasmanian waters, *Aliivibrio,* alongside other Vibrionaceae and unclassified Mycoplasmoidaceae, reaches peak populations in fish within 7–9 months following the winter-time transfer of juvenile smolt from freshwater hatcheries. Genome biology-based analyses [26] attribute colonization to a range of capabilities, including attachment, quorum sensing, putative mucinases, exopolysaccharide production, biofilm formation, and interbacterial competition, potentially involving type VI secretion systems. Dysbiosis can affect salmonid species such as Atlantic salmon when they are medicated with antimicrobials [32,33], infested by sea-lice [34], or exposed to thermal stress [35]. This may be because certain drugs promote bacterial dominance or because stress induces gut functional changes. Some bacteria in the fish gut may produce effectors that lead to gut damage or dysfunction. Strains of *Aliivibrio* with genes for cytolethal distending toxin [26] could contribute to dysbiosis through genotoxicity [36]; however, no data are available at this stage on whether or how the toxin affects GI tract epithelial cells of Atlantic salmon. Some studies have linked *Aliivibrio* to increased incidence of fecal casts and inappetence [32,33,37], coinciding with gut bacterial overgrowth [31,33,35] in fish under thermally induced dysbiotic conditions. Bacterial overgrowth occurs when water temperature increases, resulting in faster growth rates that may compound thermal physiological stress and/or external tissue infections such as tenacibaculosis [34], for which antimicrobial therapy is impractical.

Regardless of the mechanistic basis, dysbiosis substantially reduces farm performance and creates an animal welfare concern. Alternative approaches, such as probiotic treatments, could be used but have not been tested specifically for overgrowth in the marine phase and are limited because, when dysbiosis occurs, an abundant, entrenched bacterial population already exists, limiting the potential effectiveness of diet-based remedies. As a result, there is a temptation to use antimicrobials instead. However, this runs counter to stewardship principles, since use for this purpose may only work temporarily and could create longer-term AMR issues. This supports the value of monitoring AMR and changes in ARGs in commensal bacteria that colonize, grow within, and are intimately associated with fish in aquaculture systems, in addition to examining ARGs from bacteria more known for active pathogenesis. These data provide a provisional baseline of ECOFF values for microbiological management of bacteria known to actively colonize salmon in the marine phase of aquaculture and to be implicated in bacterial-linked gut dysfunction distinct from infectious disease.

Because current databases tend to focus on mammalian pathogens, prior knowledge of *Aliivibrio* AMR is limited. *Aliivibrio* species description papers suggested that ampicillin and/or penicillin resistance is prevalent [38]. Studying commensal species AMR has been advocated as an important component of monitoring programs since they can contribute to the bacterial resistome [39]. Indeed, evidence shows that commensal bacteria act as reservoirs for clinically significant antimicrobials [40] and can protect pathogens within microbiomes [41]. This study seeks to fill this information gap to some extent for the genus *Aliivibrio* and specific strains from Atlantic salmon; further studies are clearly needed to assess the breadth of diversity and the capacity for uptake of ARGs inherent to Vibrionaceae.

Given the lack of data on *Aliivibrio* AMR, the susceptibility and ECOFF estimates presented here must be considered provisional and are not suitable for clinical application. First, the MIC ranges used did not fit some antimicrobials well, either being too high (meropenem, ciprofloxacin) or too low (Table 1). We could not use *Aeromonas salmonicida* as a reference for testing because this species is prohibited in Australia under biosecurity regulations. ECOFF values could only be determined for the dominant salmon-associated species, *A. finisterrensis_A.* Further strain testing and expansion of the strain pool will be necessary to obtain more precise results and better evaluate less abundant species. The results as they stand still provide a strong foundation for refined testing, including alternative antimicrobial testing. Furthermore, confirming the exact function of the detected putative ARGs, as shown in Figure 1, Table 3 and Table S1, is not feasible at this stage. ARGs in relatively unstudied bacteria such as *Aliivibrio* differ substantially from known sequences (i.e., usually <55% amino acid similarity) that have been functionally authenticated. We assigned ARG functions (Table 3, Table S1) based on conserved domains and sequence similarity to curated protein orthologies in the KEGG database. Certain proteins (CatA, APT*) are highlighted as particularly different from confirmed sequences; therefore, the predicted functions for these proteins should be interpreted with considerable caution.

The pathogenic species *Aliivibrio salmonicida* [42] carries multiple plasmids [43]. Strain VS224 contains pRVS1-224, which carries homologs of the genes *dfrA1*, *sul2*, and *tetE*, while related strains have degenerated but are very genetically similar plasmids in which the ARGs have been lost. Conceivably, pRVS1-224 could confer resistance to most drugs used in finfish aquaculture that are effective against Vibrionaceae, though this needs verification. At this stage, it is unknown how common pRVS1-224 and similar plasmids are among *Aliivibrio*, especially in salmon farming locales. This prompted an investigation in this study to determine the distribution of ARGs across the genus *Aliivibrio*, since a range of *Aliivibrio* species have been isolated from Atlantic salmon as well as several other marine faunal species, seawater, and sediment. Available genome data remain limited and, to some degree, biased toward *A. fischeri*, due to its significance as a model for faunal mutualism, bioluminescence, and quorum sensing [44]. The available genome data pool is useful because it covers a broad range of ecosystems and includes isolates obtained over several decades. From these data, the only clear exemplar of ARG lateral gene transfer is strain VS224, isolated from Norwegian Atlantic salmon in 1987, while related strains with plasmid-borne ARGs were isolated from the same region later (1995–1998). This may suggest that ARG accumulation within the genus *Aliivibrio* is limited so far, though it still has the potential to occur at local scales. Plasmid stability should also be considered, especially in relation to effective antimicrobial stewardship. Plasmids like pRVS1-224 may only have a finite presence when antimicrobial usage is restricted. Given the dataset limitations, more time-based surveys and targeted ARG analysis could help better determine ARG associations in other salmon farm-associated bacteria besides *Aliivibrio*.

Overall, within the limitations of the available data, MGE-driven acquisition appears to be the exception rather than the rule for Tasmanian salmon *Aliivibrio* isolates and across the genus *Aliivibrio*. ARG distribution largely follows species-level or genetic-group lineages. Some distinctive ARGs identified as widely distributed in genus *Aliivibrio*, for example, Bla(CARB)-related proteins, also occur in the species *Vibrio parahaemolyticus* and various related strictly marine species (such as *V. harveyi*, *V. campbellii*, *V. pomeroyi*, *V. coralliilyticus*; Figure 2) [45]. All of these species readily colonize various faunal hosts [46]. Overall, this suggests an ancient origin of natural antibiotic compound resistance among marine Vibrionaceae that conceivably enables success within microbiomes and during interactions with eukaryotic hosts, i.e., resistance to antimicrobial peptides and secondary metabolites. This occurrence has been proposed as part of a suite of capabilities evolved in bacteria to maximize fitness, particularly by managing inter-bacterial competition [47]. Though salmon isolate plasmids and integrons did not carry ARGs, they were found to carry genes that could promote fitness in terms of competition within the fish gut, including type VI secretion system structural proteins (Hcp/VgrG homologs, PAAR-like domain proteins) and effectors (S-type pyocins, lecithin retinol acyltransferase [LRAT] family proteins) as well as DNA-related defense systems (methylase-based restriction, phosphorothioation modification systems, anti-viral reverse transcriptase). The plasmids prevalent among the isolates have a low incidence of transposons and lack integrons, suggesting relative stability; continued maintenance suggests a positive contribution to fitness.

The main antimicrobials used in the Tasmanian salmon industry have included oxytetracycline and tylosin, with a shift toward trimethoprim in the last 15 years [48]. Florfenicol has only been used to control the piscirickettsiosis outbreak that occurred in 2025–2026. From these data, the salmon *Aliivibrio* had high MICs for tylosin. Resistance to tylosin may be due to the presence of numerous efflux pump proteins; however, the specific mechanism remains to be determined. Known specific tylosin-resistant determinants, such as the macrolide methyltransferase gene *ermB*, are absent, and in any case, tylosin resistance determinants have only been confirmed in Gram-positive bacteria [49].

The high tetracycline MICs may largely be due to the tetracycline resistance efflux protein Tet(35), which was found in most *Aliivibrio* isolates except *Aliivibrio* “sp028765575” strains, which have significantly lower MICs for both tetracycline and oxytetracycline. Furthermore, isolates were largely sensitive to trimethoprim–sulfamethoxazole and to trimethoprim alone. In this respect, the isolates only possess a single type III dihydrofolate reductase (*folA*) gene. A minority of strains demonstrate higher-than-average MICs to trimethoprim alone (MICs: five strains 16 μg/mL one strain 32 μg/mL). Strains with 16–32 μg/mL MICs included strains that were sequenced (A32, S3TY1, S2TY2). However, these strains were not found to carry genes for alternative dihydrofolate reductases (i.e., *dfrA*, *dfrB*), suggesting undefined traits may aid protection. It should be noted that strain VS224, the plasmid of which contains integrons bearing *dfrA1*, also has not been specifically evaluated for trimethoprim resistance, at least within the available literature. Further analysis is clearly needed to determine the prevalence of trimethoprim resistance and also discover if this could be problematic with continued occasional reliance on trimethoprim for prophylaxis.

The high MICs (>512 μg/mL) observed for ampicillin, benzylpenicillin, and carbenicillin, and the lack of resistance to cefepime and ceftriaxone are consistent with carbenicillinase/ Bla(CARB) substrate-binding properties [50]. The presence of Bla(TEM) homologs in two *Aliivibrio* genomes is noteworthy; however, whether this contributes to broad-spectrum penam resistance is yet to be determined.

The salmon strains demonstrate high florfenicol MIC values (MIC > 256 μg/mL) but not with chloramphenicol (MIC ≤ 1–2 μg/mL, zones ≥ 20 mm). The source of florfenicol resistance is unclear since standard ARGs connected to the phenotype were absent, including MFS-type efflux proteins FloR (Cml), FexA/FexB and CmlR/Cmx; 23S rRNA (adenine-2503) methyltransferase Cfr; and relevant hydrolases/esterases (e.g., EstDL136, [51]). The presence of putative chloramphenicol and streptogramin acetyltransferases (*catA*, *catB*, *vatF*) did not correspond to high chloramphenicol MIC values. The presence of predicted *cpt* and *catB* in integrons (Table 3) could suggest that greater-than-normal chloramphenicol background resistance is possible in other *Aliivibrio* species, but this still needs to be tested. PCR-based detection of *floR* does not always correlate with florfenicol or other amphenicol resistance [52], and contradictions between phenotype and ARG-related genotypes can occur [53], as is apparent here. This could reflect inaccurate assumptions about protein function. For example, the CatA-like homolog (possessing the xenobiotic acetyltransferase domain) common to *Aliivibrio* is quite diverged from functionally verified homologs. It may not affect chloramphenicol as a substrate. Various DHA-family MFS transporters that are weakly homologous to FloR proteins (27–35% similarity) are present, but substrates for these proteins, as well as most of the other efflux proteins detected, remain undefined. A series of efflux proteins could confer additive resistance to florfenicol; however, this requires further research to confirm. This could be initially determined by performing proteomic and/or transcriptomic analyses on strains with different AMR phenotypes to identify which genes and proteins respond to antimicrobial pressure.

Antimicrobials known to be effective against most human pathogenic *Vibrio* spp. that were evaluated were also effective against *Aliivibrio* isolates, especially ciprofloxacin and meropenem, which inhibited growth at the lowest tested MIC (0.5 μg). Other effective antimicrobials (rifamycins, oxolinic acid) lack official breakpoint data for Vibrionaceae, so the reported data are provisional. Applying these antimicrobials to finfish (in Australia) is currently not permitted, and use could potentially lead to acquisition of ARGs [54] and direct toxicity [32].

## 4. Materials and Methods

### 4.1. Atlantic Salmon Aliivibrio Isolates Evaluated

A total of 50 *Aliivibrio* isolates from the digesta or gut mucosa of Atlantic salmon were investigated. The strains comprise three undescribed species as defined in Reid et al. [26] and the Genome Taxonomy Database [55]. The strains were routinely grown aerobically on marine agar (MA) at 25 °C. MA comprises 0.5% (*w*/*v*) bacteriological peptone (Oxoid LP0037B, Thermo Scientific, Scoresby, VIC, Australia), 0.2% (*w*/*v*) yeast extract (Oxoid LP0021), 2.5% (*w*/*v*) sea salts (Instant Ocean^TM^, Aquarium Systems via Aquarium Suppliers Pty Ltd., Banksmeadow, NSW, Australia), and 1.5% (*w*/*v*) agar (Gelita, Beaudesert, QLD, Australia). All strains were stored at −80 °C in marine broth containing 30% (*v*/*v*) glycerol (Sigma-Aldrich, Bayswater, VIC, Australia).

### 4.2. Antimicrobial Panel and Breakpoint Adoption

For MIC testing, we used 24 antimicrobial agents covering most drug classes. We purchased all antimicrobials from Sigma-Aldrich. Antimicrobial stock solutions were prepared by weighing 51.2 mg of each chemical, dissolving it in an appropriate solvent [56], and diluting 10-fold to a final working concentration of 512 µg/ mL [57]. For antimicrobials already in solution (tylosin), 360 µL was added to 640 µL of distilled water and diluted 10-fold. For disk diffusion testing mast-rings (Southern Biological, Alphington, VIC, Australia) containing streptomycin (10 μg), sulfatriad (sulfamerazine, sulfadiazine, sulfathiazole) (200 μg), chloramphenicol (25 μg), tetracycline (25 μg), ampicillin (10 μg) and benzylpenicillin (1 IU, ~0.6 μg) was utilized. Furthermore, disks containing cefepime (10 μg), ceftriaxone (10 μg), and trimethoprim–sulfamethoxazole (1.25/23.75 μg) (Liofilchem; Integrated Sciences, Chatswood, NSW, Australia) were also evaluated. Because no breakpoint data are available for *Aliivibrio* species, we used presumptive breakpoint values to estimate potential susceptibilities. We adopted these presumptive breakpoints from CLSI VET03 guidelines [57] and literature sources [53,58,59,60,61,62,63,64,65,66,67] for *Vibrio* species (Table 4). We used *Vibrio*-derived data since *Aliivibrio* is a close relative and may also be tested in NaCl-supplemented media.

### 4.3. Cultivation Conditions

MIC determination used the broth microdilution method with 96-well microtiter plates, as outlined in VET03 and VET04 [57,62]. The test medium required salt supplementation for two reasons. Firstly, the hindgut of Atlantic salmon in its marine phase is saline (400 mOsm/L, ~13 ppt salinity) to maintain osmotic balance [68]. Second, salt supports the growth of *Aliivibrio* isolates, which cannot grow in media with ≤0.5% (*w*/*v*) NaCl. As a result, for MIC and disk diffusion testing, we supplemented Mueller–Hinton broth and agar with 1.0% (*w*/*v*) NaCl (MHB + NaCl), as suggested for halophilic *Vibrio* species [69]. Incubation was also performed at 25(±1) °C, as this temperature supports optimal *Aliivibrio* spp. growth. The isolates are incapable of growing at ≥33 °C (growth range: ~4 °C to 32 °C, [26]).

### 4.4. MIC Testing

MIC testing used 96-well U-bottom (Greiner) microplates (Integrated Sciences, Chatswood, NSW, Australia). Duplicate wells were inoculated with 50 µL of MHB + NaCl before 50 µL of antimicrobial solution was pipetted into each well of the first column. A cascade two-fold dilution was then applied from column 1 to 10, starting with a 512 µg/mL antimicrobial stock solution, yielding an MIC range of 0.5 to 256 µg/mL Column 11 of microplates contained 100 µL of MHB + NaCl as a negative growth control, and column 12 contained 50 µL of MHB + NaCl medium and 50 µL of bacterial suspension as a positive growth control. Cultures were grown at 25 °C for 24–28 h in MHB + NaCl broth and were used for inoculation of 96-well plates. We prepared these broth cultures by suspending 3–4 single colonies of *Aliivibrio* isolates from MA in 10 mL of MHB + NaCl and growing them to approximately McFarland 0.5 turbidity. Turbidity was measured by spectrophotometric analysis (OD_600_ 0.08–0.13) [29]. To confirm that the correct colony-forming units (CFU) per ml concentration was reached, serial dilutions of cultures were performed at 24–28 h, and plate counts were routinely performed. Cultures of approximately 1 × 10^8^ CFU/ mL were diluted to obtain 1 × 10^6^ CFU/;mL 50 µL of bacterial suspension was inoculated into columns 1 to 10 containing 50 µL of MHB + NaCl plus antimicrobial solution, yielding a final concentration of 5 × 10^5^ CFU/ mL or 5 × 10^4^ CFU/well [29]. Plates were incubated for 24 h and analyzed by spectrophotometry using a 96-well plate reader (BMG LABTECH, Ortenburg, Germany) at OD_600_. MIC (100% growth inhibition), MIC50 (50% inhibition), and MIC90 (90% inhibition) values were calculated by comparing optical density (OD) at 600 nm of the test samples (*ODx*) against that of the no-antimicrobial control OD (*ODc*):

$$\%\;inhibition=\left(1-\frac{ODx}{ODc}\right)\times 100$$

To account for experimental variation and potency, the experiment was performed twice at different times by two different individuals. The results shown indicate mean responses. Reference strains *Escherichia coli* ATCC 25922 and *Enterococcus faecalis* ATCC 29212 were also used to ensure antimicrobial potency; however, these strains were evaluated in standard Mueller–Hinton broth at 35(±1) °C. *Aeromonas salmonicida* type strain ATCC 33658, normally used as a reference for aquaculture MIC testing [57,69], was not available for our use. This is because the species is exotic to Australia and forbidden for experimental use [70].

### 4.5. Disk Diffusion Antimicrobial Susceptibility Assay

Three to five bacterial colonies grown from purified isolates on marine agar were inoculated into 10 mL of MHB + NaCl for incubation at 25(±1) °C for 24–28 h to reach 0.5 McFarland Standard. This was verified by spectrophotometric analysis (absorbance at 600 nm between 0.08 and 0.13) and by counting colonies from serial dilutions (10^−1^ to 10^−7^) plated as 0.1 mL onto MHB + NaCl agar (1.5% agar). As per the CLSI guidelines VET03, sterile swabs were dipped into cultures containing approximately 1 × 10^8^ CFU/ mL and spread over plates to achieve uniform lawn cultures [57]. We removed mast rings and individual disks from containers using ethanol-flamed, sterilized tweezers and placed them on agar. Plates were incubated for 24 h at 25(±1) °C, and inhibition zones were recorded as the diameter of cleared halos present around the disks, ensuring that the measured diameter intersected the antimicrobial disk [57].

### 4.6. Determination of Wild-Type Cutoff Values and MIC Interpretation

Wild-type cutoff values were generated using Normalized Resistance Interpretation (NRI) [71] utilizing an automated spreadsheet (2019 version) available from http://www.bioscand.se/nri/ (accessed on 5 September 2026). These estimations separated the bacterial population into wild-type strains with no acquired resistance traits and strains with presumed acquired resistance traits. Isolates with an MIC above the cutoff wild-type value (CO_wt_), synonymous with epidemiological cutoff (ECOFF) values, were classified as “non-wild type” strains. Using this approach, we estimated ECOFF values for species-level groups [72].

### 4.7. ARG Detection in Aliivibrio Genomes

Assembled whole-genome sequence data from salmon bacterial isolates ([26], 21 genomes) and *Aliivibrio* species genomes (n = 118) (Table S1) were obtained from NCBI and surveyed for ARGs. We also investigated the genomes for the presence and location of MGEs. We initially used the following database/search engines: CARD version 4.01 [73], MOB-Recon version 3.1.9 [74], and IntegronFinder version 2.0.5 [75]. We performed MOB-Recon and IntegronFinder analyses using the Galaxy Australia cloud server [76]. We supplemented these analyses with manual inspection of regions highlighted in the MGE search results. We also compared representative ARG orthologs from the BRITE orthology in the KEGG database [77] to *Aliivibrio* genomes using systematic local BLASTP searches implemented in Unipro UGENE version 53.1 [78]. A specific ARG was defined across *Aliivibrio* spp. as having >62% amino acid similarity (>55% for outlier species “*Aliivibrio_A kagoshimensis*”) to distinguish paralogous gene clusters. We used these ARG similarity thresholds for practical reasons when analyzing data for both the salmon *Aliivibrio* isolates and the entire genus *Aliivibrio*. The thresholds correspond to the expected divergence observed for bacterial genera and closely related genera [79]. To ensure accurate protein family assignment, we aligned representative isolate proteins against reference sequences from the KEGG addendum set and compared them in phylograms. We aligned the proteins using MAFFT and generated trees with FastTree 2 [80] via phylogeny.fr using the “One-Click” process [81]. We visualized ARG presence/absence for each strain using a heat map generated with Morpheus (Broad Institute, https://software.broadinstitute.org/morpheus/, accessed on 5 September 2026). We hierarchically clustered ARGs and strains using Euclidean distances and complete linkage. We performed SIMPROF testing [82] to assess cluster significance in relation to species association and define the core set of genes for the genus *Aliivibrio*. We performed SIMPROF analysis in Primer version 7.0.24 (Primer-e, Quest Research Ltd., Auckland, New Zealand) and defined groups at an alpha level of 0.05. We assessed the genetic similarity of detected plasmids derived from Mob-Recon by determining average nucleotide identity (ANI) using OrthoANI [83].

## 5. Conclusions

The results suggest little evidence of extensive lateral ARG transfer among Tasmanian-region Atlantic salmon *Aliivibrio* isolates overall, based on available data. Data for the overall Aliivibrio genus suggest the potential to acquire ARGs via MGEs. However, the provisional data also suggest that this genus has high natural resilience to many common antimicrobials. Furthermore, the susceptibility patterns of the *Aliivibrio* isolates indicate limited options to control *Aliivibrio* populations if that ever becomes the goal. Trimethoprim and trimethoprim–sulfamethoxazole represent the best options but require vigilant antimicrobial usage management. Further studies characterizing *Aliivibrio* MICs could include a greater range of strains and MIC values to refine ECOFF values. This would help define effective antimicrobials and establish new treatment regimes, if required. Furthermore, more targeted genome- and cultivation-based experiments would help determine the extent of ARG occurrence in salmon farming waters, especially during summer, to assess the risk of ARG transfer to microbiome-dominant bacteria.

## Figures and Tables

**Table 1:** Median MIC_50_ and MIC_90_, sensitivity, MIC range and epidemiological cut off concentration values for *Aliivibrio* isolatesfrom Atlantic salmon.

Species Tested	Antimicrobial	MIC (μg/mL)										ECOFF	Sensitivity% ^a^	MIC50 (MIC90) ^b^	Total MIC Range ^c^
		0.5	1	2	4	8	16	32	64	128	256				
*Aliivibrio finisterrensis_A*	tetracycline	-	-	-	-	-	25	12	-	2	-	128	0	16 (64)	16–128
	oxytetracycline	-	-	-	-	3	14	19	4	-	-	128	8	32 (64)	4–64
	erythromycin	-	-	-	3	5	8	13	6	4	1	128	0	32 (128)	4–256
	tylosin	-	-	-	-	-	-	1	3	19	17	≥512 ^d^	0	128–256 (256)	32–256
	florfenicol	-	-	-	-	-	-	-	-	-	40	≥512 ^d^	0	>256	>256
	chloramphenicol	11	19	10	-	-	-	-	-	-	-	2	100	1 (1)	1–2
	ampicillin	-	-	-	-	-	-	-	-	22	18	≥512 ^d^	0	128 (256)	128–256
	benzylpenicillin	-	-	-	-	-	-	-	-	3	37	≥512 ^d^	0	256 (256)	128–256
	carbenicillin	-	-	-	-	-	-	-	12	14	14	512	0	64–256 (256)	64–256
	meropenem	-	-	-	-	-	-	-	-	-	-	<0.5 ^d^	100	no growth	no growth
	polymyxin B	-	-	-	4	11	20	5	-	-	-	64	0	16 (32)	4–32
	gentamicin	-	-	-	-	-	-	-	-	40	-	256	0	128 (128)	128
	streptomycin	-	-	-	-	-	-	-	-	28	12	512	0	128 (256)	128–256
	kanamycin	-	-	-	-	-	-	-	-	26	14	256	0	128 (256)	128–256
	rifampicin	23	15	-	2	-	-	-	-	-	-	1	95	0.5 (1)	0.5–8
	oxolinic acid	7	12	11	6	3	1	-	-	-	-	8	90	1–2 (2)	0.5–8
	ciprofloxacin	-	-	-	-	-	-	-	-	-	-	<0.5 ^d^	100	no growth	no growth
	trimethoprim	3	14	14	3	-	5	1	-	-	-	8	85	1–2 (2)	0.5–32
	sulfamerazine	-	-	-	-	-	-	-	2	12	26	512	35	>256	64–256
	sulfadiazine	-	-	-	-	-	-	-	-	9	31	512	22	>256	128–256
*Aliivibrio* sp028765545	tetracycline	-	-	-	-	-	6	-	-	-	-	ND	0	16 (64)	16
	oxytetracycline	-	-	-	-	-	-	6	-	-	-	ND	0	32 (64)	8–64
	erythromycin	-	-	-	-	-	-	6	-	-	-	ND	0	32 (128)	32
	tylosin	-	-	-	-	-	-	-	-	5	1	ND	0	128–256 (256)	128–256
	florfenicol	-	-	-	-	-	-	-	-	-	6	ND	0	>256	>256
	chloramphenicol	-	5	1	-	-	-	-	-	-	-	ND	100	1 (1)	1–2
	ampicillin	-	-	-	-	-	-	-	-	4	2	ND	0	256	128–256
	benzylpenicillin	-	-	-	-	-	-	-	-	-	6	ND	0	256 (256)	256
	carbenicillin	-	-	-	-	-	-	-	-	1	5	ND	0	256 (256)	128–256
	meropenem	-	-	-	-	-	-	-	-	-	-	ND	100	no growth	no growth
	polymyxin B	-	-	-	-	2	4	-	-	-	-	ND	0	16 (32)	8–16
	gentamicin	-	-	-	-	-	-	-	-	6	-	ND	0	128 (128)	128
	streptomycin	-	-	-	-	-	-	-	-	3	3	ND	0	128–256 (256)	128–256
	kanamycin	-	-	-	-	-	-	-	-	4	2	ND	0	128 (256)	128–256
	rifampicin	3	3	-	-	-	-	-	-	-	-	ND	100	0.5	0.5–1
	oxolinic acid	1	3	2	-	-	-	-	-	-	-	ND	0	1–2	0.5–2
	ciprofloxacin	-	-	-	-	-	-	-	-	-	-	ND	100	no growth	no growth
	trimethoprim	-	6	-	-	-	-	-	-	-	-	ND	100	1	1
	sulfamerazine	-	-	-	-	-	-	-	-	3	3	ND	50	>256	128–256
	sulfadiazine	-	-	-	-	-	-	-	-	2	4	ND	33	>256	128–256
*Aliivibrio* sp028765575	tetracycline	-	-	-	-	3	1	-	-	-	-	ND	0	8 (16)	8–16
	oxytetracycline	-	-	2	2	-	-	-	-	-	-	ND	100	2 (4)	2–4
	erythromycin	-	-	-	-	-	-	4	-	-	-	ND	0	32 (128)	32
	tylosin	-	-	-	-	-	-	-	-	2	2	ND	0	128–256 (256)	128–256
	florfenicol	-	-	-	-	-	-	-	-	-	4	ND	0	>256	>256
	chloramphenicol	1	2	1	-	-	-	-	-	-	-	ND	100	1 (1)	1–2
	ampicillin	-	-	-	-	-	-	-	-	3	1	ND	0	128 (256)	128–256
	benzylpenicillin	-	-	-	-	-	-	-	-	1	3	ND	0	256 (256)	128–256
	carbenicillin	-	-	-	-	-	-	-	-	2	2	ND	0	256 (256)	128–256
	meropenem	-	-	-	-	-	-	-	-	-	-	ND	100	no growth	no growth
	polymyxin B	-	-	-	1	-	3	-	-	-	-	ND	0	16 (32)	4–16
	gentamicin	-	-	-	-	-	-	-	-	4	-	ND	0	128 (128)	128
	streptomycin	-	-	-	-	-	-	-	-	1	3	ND	0	256 (256)	128–256
	kanamycin	-	-	-	-	-	-	-	-	4	-	ND	0	128 (128)	128
	rifampicin	3	1	-	-	-	-	-	-	-	-	ND	100	0.5	1–2
	oxolinic acid	-	3	1	-	-	-	-	-	-	-	ND	0	1	1–2
	ciprofloxacin	-	-	-	-	-	-	-	-	-	-	ND	100	no growth	no growth
	trimethoprim	-	3	1	-	-	-	-	-	-	-	ND	100	1	1–2
	sulfamerazine	-	-	-	-	-	-	-	-	1	3	ND	25	>256	128–256
	sulfadiazine	-	-	-	-	-	-	-	-	-	4	ND	0	>256	256

^a^ Provisional values based on presumptive breakpoints as described in the methodology (Section 4.2). ^b^ MIC90 values are shown in parentheses. ^c^ Total range based on MIC50 and MIC90 data. ^d^ ECOFFF values could not be calculated, and thus only minimum and maximum threshold values are shown. No estimates (ND) were possible for species sp028765545 and sp028765575 due to small strain numbers.

**Table 2:** Disc diffusion-based sensitivity of by Atlantic salmon *Aliivibrio* strains for a select panel of antibiotics.

Antimicrobial	Disk Concentration (μg)	*Aliivibrio finisterrensis_A*			*Aliivibrio* sp028765545			*Aliivibrio* sp028765575		
		S ^a^	I	R	S	I	R	S	I	R
		No. isolates								
Chloramphenicol	25	40	-	-	6	-	-	4	-	-
Tetracycline	25	-	24	18	-	2	4	4	-	-
Sulfatriad	200	25	-	15	-	5	1	-	-	4
Streptomycin	10	-	-	40	-	-	6	-	-	4
Ampicillin	10	-	-	40	-	-	6	-	-	4
Benzylpenicillin	10	-	-	40	-	-	6	-	-	4
Cefepime	10	40	-	-	6	-	-	4	-	-
Ceftriaxone	10	40	-	-	6	-	-	4	-	-
Trimethoprim-sulfamethoxazole	1.25/23.75	29	11	-	5	1	-	4	-	-

^a^ Determinations of sensitivities and resistances are based on presumptive break points only as indicated in Section 4.2.

**Table 3:** ARGs present on MGEs amongst genome sequenced *Aliivibrio* strains.

Species	Strains	Predicted ARGs Present on MGEs	Genetic Location
*A. fischeri*	5F146, LB10LO6, emors.5.1t, emors.9.2, MJ12	aminoglycoside phosphotransferase family protein	CALIN
*A. fischeri*	MJ11, VCW2E2, emors.5.1o, emors.5.1t, emors.5.2		Intact integron
*A. sifiae*	CL1, CL3		CALIN
*A. fischeri*	VLS2, SR5, VCW2E2, emors.5.2, emors.9.1, emors.11.2	chloramphenicol 3-O phosphotransferase (*cpt*)	CALIN
*A. logei*	1S159		
*A. sifiae*	CL4, CL5		
*A. wodanis*	NVI 06/09/139		
*A. fischeri*	5F7	chloramphenicol acetyltransferase type B (*catB*)	CALIN
*A. wodanis*	NVI 06/09/139		
*A. salmonicida*	SP1		
*A. salmonicida*	VS224, 15-Vs224, 13-BacKlon4, 12-BacKlon1	trimethoprim-resistant dihydrofolate reductase (*dfrA1*)	Plasmid
	VS224, 15-Vs224, 13-BacKlon4, 12-BacKlon1	sulfonamide-resistant dihydropteroate synthase (*sul2*)	
	VS224, 15-Vs224, 13-BacKlon4, 12-BacKlon1, 14-Bac1099	DHA1 family MFS-type tetracycline efflux antiporter (*tetE*)	

**Table 4:** Antimicrobials evaluated, including their interpretive MIC breakpoints and Inhibition zones as adapted from CLSI, EUCAST, and literature sources.

Antimicrobials	Disk Concentration (μg)	MIC Breakpoints (μg/mL)			Disk Diffusion Breakpoints (mm)			Reference Source(s)
		S ^a^	I	R	S	I	R	
Tetracycline, oxytetracycline	25	≤4	8	≥16	≥15	12–14	≤11	[58,59,60]
Erythromycin, tylosin		≤0.5	4	≥8	~	~	~	[59]
Florfenicol		≤4	8	≥16	~	~	~	[59,60]
Chloramphenicol	25	≤8	16	≥32	≥18	13–16	≤12	[58,59,60]
Ampicillin, Carbenicillin	10	≤8	16	≥32	≥17	14–16	≤13	[58,59,60]
Penicillin G	1 IU	~	~	≥64	≥17	14–16	≤13	[62] ^b^
Cefepime		~	~	≥16	≥24	19–24	≤18	[63]
Ceftriaxone		~	~	≥8	≥24	19–24	≤18	[64]
Meropenem		≤0.5		≥1	≥23	20–22	≤19	[65]
Streptomycin	10	≤16	32	≥64	≥17	13–16	≤12	[60,61]
Gentamicin		≤2	4	≥8	~	~	~	[58,60]
Kanamycin		≤8	16	≥32	~	~	~	[60]
Oxolinic Acid		≤2	4	≥8	~	~	~	[59]
Ciprofloxacin		≤0.06	0.12–0.5	≥1	~	~	~	[62,65]
Rifampicin		~	~	≥4	~	~	~	[66] ^b,c^
Polymyxin B		≤2	4	≥8	~	~	~	[67]
Trimethoprim		≤8	~	≥16	~	~	~	[59,60]
Sulfamerazine, sulfadiazine		≤256	~	≥512	~	~	~	[61] ^b,d^
Sulfatriad	200	~	~	~	≥16	11–15	≤10	[61] ^b,d^
Trimethoprim–sulfamethoxazole	1.25/23.75	~	~	~	≥16	11–15	≤10	[62,65]

^a^ Abbreviations: S = Sensitive, I = Intermediate Resistant R = Resistant. ^b^ Breakpoints not available in either EUCAST or CLSI guidelines. ^c^ Adapted from rifamixin. ^d^ Adapted from sulfisoxazole.

## Data Availability

In addition to data presented here, the original data is available in a publicly accessible repository: Mendeley Data (https://doi.org/10.17632/gyrpd8hkdw.1).

## Supplementary Materials

The following supporting information can be downloaded at https://www.mdpi.com/article/10.3390/antibiotics15090888/s1, Table S1: Antibiotic resistance gene codes, protein designation, KEGG ontology codes, representative proteins for *Aliivibrio* genomes. Table S2: Genome information for *Aliivibrio* strains investigated in the study. Table S3: Details of intact integrons present in *Aliivibrio* strains including ARG presence. Table S4: Details of CALIN present in *Aliivibrio* strains including ARG presence. Figure S1: Comparison of CatB (chloramphenicol acetyltransferase type B) and Vat (virginiamycin/streptogramin acetyltransferases) homologous proteins from *Aliivibrio* and reference sequences from KEGG orthology. The outgroup sequence is GlmU (WP_017020791.1). The tree was generated using phylogeny.fr (MAFFT/FastTree).

## Abbreviations

The following abbreviations are used in this manuscript:

GI	Gastrointestinal
AMR	Antimicrobial resistance
ARGs	Antimicrobial resistance genes
IU	International Unit (as a measure of activity)
MGEs	Mobile genetic elements
MIC	Minimum inhibitory concentration
MA	Marine agar
MHB	Mueller–Hinton medium
GTDB	Genome Taxonomy Database
OD	Optical density
APT*	Putative aminoglycoside phosphotransferase family protein
CALIN	Clusters of AttC sites lacking (integron) integrases
NRI	Normalized Resistance Interpretation
CO_wt_	Cut-off wild-type value
ECOFF	Epidemiological cutoff
ANI	Average nucleotide identity
CDT	Cytolethal distending toxin
