# Supplementary material for: Antimicrobial Resistance and Associated Genetic Determinants of Aliivibrio Colonizing the Gastrointestinal Tract of Farmed Atlantic Salmon (Salmo salar L.)
Source: Antibiotics (Basel). 2026 Sep 10;15(9):888. doi: 10.3390/antibiotics15090888 (PMC13603697; doi:10.3390/antibiotics15090888)
Supplement: Supplementary file 1 [file antibiotics-15-00888-s001.zip › Supplementary data Aliivibrio AMR paper August 21 2026.pdf]

Table S1. Antibiotic resistance gene codes, protein designation, KEGG ontology codes, representative proteins for *Aliivibrio* genomes

Gene code	Protein designation	KEGG code	*Aliivibrio* representative protein accession code	Notes
cpt	chloramphenicol 3-O phosphotransferase	K18554	WP_446713025.1	
catA-like	chloramphenicol O-acetyltransferase type A	K19271	WP_130043164.1	
catB9	chloramphenicol O-acetyltransferase type B	K00638	WP_151656088.1	Figure S1
catB-like (1)[a]	chloramphenicol O-acetyltransferase type B	K00638	WP_155684076.1	Figure S1
catB-like (2)	chloramphenicol O-acetyltransferase type B	K00638	WP_061037187.1	Figure S1
vat(F)	virginiamycin A acetyltransferase	K18234	WP_430520439.1	Figure S1
blaCARB	beta-lactamase (CARB-17) class A	K19127, K17836	WP_418193289.1, WP_233940049.1	Figure 2
blaTEM	beta-lactamase class A TEM	K18698	WP_416503101.1	
tet(35)	tetracycline resistance efflux pump	K18218	WP_274419719.1	
mdlB	ATP-binding cassette, subfamily B, multidrug efflux pump	K18890	WP_122036386.1	
tet(E)/tetA	MFS transporter, DHA1 family, tetracycline resistance protein	K08151	WP_032490168.1	Present in pRVS1-224 of strain VS224
aph(3'-Ia)	aminoglycoside 3'-phosphotransferase I	K19272	WP_000018329.1	
*APT**	aminoglycoside phosphotransferase family protein	~	WP_390521813.1	
dfrA1	dihydrofolate reductase (trimethoprim resistance protein)	K18589	WP_001355915.1	Present in pRVS1-224 of strain VS224
sul2	dihydropteroate synthase type 2	K18824	WP_063855540.1	Present in pRVS1-224 of strain VS224
eptA (1)	lipid A ethanolaminephosphotransferase	K03760	WP_274439853.1	2 distinct genes in strains S3TY1, S2TY2
eptA (2)	lipid A ethanolaminephosphotransferase	K03760	WP_446713160.1	
eptA (3)	putative phosphoethanolamine transferase	~	WP_133415070.1	
qnr	fluoroquinolone resistance protein	K18555	WP_130087113.1	
macB	macrolide transport system ATP-binding/permease protein	K05685	WP_105055689.1	
mdtK (1)	MATE family, multidrug efflux pump	K26939	WP_233941130.1	
mdtK (2)	MATE family, multidrug efflux pump	K26939	WP_012551309.1	
mdtK (3)	MATE family, multidrug efflux pump	K26939	WP_130087224.1	
mepA (1)	MATE family, multidrug efflux pump	K26937	WP_130045318.1	
mepA (2)	MATE family, multidrug efflux pump	K26937	WP_130047331.1	
mepA (3)	MATE family, multidrug efflux pump	K26937	WP_151653098.1	
mepA (4)	MATE family, multidrug efflux pump	K26937	WP_182700092.1	
mepA (5)	MATE family, multidrug efflux pump	K26937	WP_061002244.1	
mepA (6)	MATE family, multidrug efflux pump	K26937	WP_155689175.1	

mepA (7)	MATE family, multidrug efflux pump	K26937	WP_428197244.1	
mepA (8)	MATE family, multidrug efflux pump	K26937	WP_300181053.1	
mepA (9)	MATE family, multidrug efflux pump	K26937	WP_130043574.1	
vcaM	ATP-binding cassette, subfamily B, multidrug efflux pump	K18893	WP_486315345.1	
mdlA	ATP-binding cassette, subfamily B, multidrug efflux pump	K18889	WP_130087818.1	
bcr (1)	MFS transporter, DHA1 family, multidrug resistance protein	K07552	WP_045100863.1	
bcr (2)	MFS transporter, DHA1 family, multidrug resistance protein	K07552	WP_065622204.1	
bcr (3)	MFS transporter, DHA1 family, multidrug resistance protein	K07552	WP_069594629.1	
ermB	MFS transporter, DHA2 family, multidrug resistance protein	K03446	WP_446702020.1	
emrE (1)	small multidrug resistance protein	K03927	WP_300174057.1	
emrE (2)	small multidrug resistance protein	K03927	WP_060992221.1	
emrA (1)	multidrug resistance efflux system	K03543	WP_339967831.1	
emrA (2)	multidrug resistance efflux system	K03543	WP_300173127.1	
emrA (3)	multidrug resistance efflux system	K03543	WP_017021551.1	
emrA (4)	multidrug resistance efflux system	K03543	WP_026029279.1	
EmrA family (1)	EmrA family RND transporter	~	WP_105063673.1	
EmrA family (2)	EmrA family RND transporter	~	WP_105056288.1	
EmrA family (3)	EmrA family RND transporter	~	WP_105064103.1	
EmrA family (4)	EmrA family RND transporter	~	WP_446711788.1	
emrD (1)	MFS transporter, DHA1 family, 2-module integral membrane pump	K08154	WP_130043673.1	
emrD (2)	MFS transporter, DHA1 family, 2-module integral membrane pump	K08154	WP_017022627.1	
emrD (3)	MFS transporter, DHA1 family, 2-module integral membrane pump	K08154	WP_065591790.1	
acrA (1)	membrane fusion protein, multidrug efflux system	K03585	WP_005596437.1	
acrA (2)	membrane fusion protein, multidrug efflux system	K03585	WP_105063719.1	
AcrA family (3)	AcrA family membrane fusion protein	~	WP_063653498.1	
acrB (1)	multidrug efflux system component	K18138	WP_130045454.1	
acrB (2)	multidrug efflux system component	K18138	WP_336505629.1	
acrB (3)	multidrug efflux system component	K18138	WP_063647724.1	
acrB (4)	multidrug efflux system component	K18138	WP_065604819.1	
AcrB family (5)	multidrug efflux system component	~	WP_371968705.1	
AcrB family (6)	multidrug efflux system component	~	WP_017021790.1	
AcrB family (7)	multidrug efflux system component	~	WP_005422054.1	

AcrB family (8)	multidrug efflux system component	~	WP_473577718.1	
AcrB family (9)	multidrug efflux system component	~	WP_155697401.1	
AcrB family (10)	multidrug efflux system component	~	WP_135354157.1	
AcrB family (11)	multidrug efflux system component	~	WP_375320805.1	
AcrB family (12)	multidrug efflux system component	~	WP_453051847.1	
AcrB family (13)	multidrug efflux system component	~	WP_145574753.1	
AcrB family (14)	multidrug efflux system component	~	WP_664601153.1	
acrB (15)	multidrug efflux system component	K18138	WP_122033548.1	
acrB (16)	multidrug efflux system component	K18138	WP_151654807.1	
acrB (17)	multidrug efflux system component	K18138	WP_430520733.1	
acrB (18)	multidrug efflux system component	K18138	WP_300178421.1	
acrB (19)	multidrug efflux system component	K18138	WP_430519686.1	

[a]Numbers in parentheses denote distinct gene clusters (based on protein similarity >62% for *Aliivibrio* spp., >55% for "*Aliivibrio_A kagoshimensis*"). Notes - Figure S1 shows a comparison of CatB homologs to characterised proteins. Bla(CARB) homologs are shown in Figure 2 against related proteins including CARB-17 from *Vibrio parahaemolyticus*. Plasmid pRSV1-224 is from *Aliivibrio salmonicida* strain VS224. Both *Aliivibrio finisterrensis_A* strains S3TY1 and S2TY2 have two near identical copies of EptA (protein cluster WP_274439853).

Table S2. Genome information *for Aliivibrio* strains investigated in the study.

Species	Strain	GenBank Accessions	Contigs	Total size	GC Content	Isolation Source	Collection Year	Isolation Country	Host Name	Plasmids (sizes in bp)
Aliivibrio "friggae"	ATCC 15382	CM170638:CM170639	2	4377311	39.00	Pacific cod skin	1964	USA	*Gadus macrocephalus*	~
Aliivibrio "friggae"	SR10	CM170642:CM170643	2	4316750	39.00	squid light organ	1995	France	*Sepiola robusta*	~
Aliivibrio "friggae"	SR9	CM170644:CM170645	2	4311151	39.00	squid light organ	1995	France	*Sepiola robusta*	~
Aliivibrio "friggae"	SR6	CM170646:CM170647	2	4421717	39.00	squid light organ	1995	France	*Sepiola robusta*	~
Aliivibrio "friggae"	SA12	CM170650:CM170651	2	4342586	39.00	squid light organ	1995	France	*Sepiola affinis*	~
Aliivibrio "friggae"	SR8	CM170648:CM170649	3	4321147	39.00	squid light organ	1995	France	*Sepiola robusta*	5530
Aliivibrio "friggae"	EL58 (or EL581)	OMPC00000000	12	4297465	39.02	Gorgonian coral	2015	Portugal	*Eunicella labiata*	~
Aliivibrio "thorii"	MdR7	CM170640:CM170641	2	4488222	38.50	coastal seawater	1989	USA	~	~
Aliivibrio "thorii"	SA5	CM170652:CM170653	2	4531799	38.50	squid light organ	1995	France	*Sepiola affinis*	~
Aliivibrio "thorii"	SA6	JBXXRD00000000	3	4699157	38.50	squid light organ	1995	France	*Sepiola affinis*	166899
Aliivibrio "vili" sp001690985	1S165	MAJT00000000	90	4317411	38.66	seawater	2006	USA	~	~
Aliivibrio "vili" sp001690985	1S175	MAJS00000000	99	4320255	38.66	seawater	2006	USA	~	~
Aliivibrio "vili" sp001690985	1S128	MAJV00000000	189	4320262	38.62	seawater	2006	USA	~	~
Aliivibrio finisterrensis	LMG 23869	WBVP00000000	143	3916971	39.52	Manila clam	2004	Spain	*Ruditapes philippinarum*	~
Aliivibrio finisterrensis_A	S2TY2	JARACS000000000	57	4341966	38.92	gastrointestinal mucosa	2018	Australia	*Salmo salar*	176281
Aliivibrio finisterrensis_A	S3TY1	JARACT000000000	60	4342759	38.93	gastrointestinal mucosa	2018	Australia	*Salmo salar*	176260
Aliivibrio finisterrensis_A	A32	SEZJ00000000	69	4169111	39.06	hind gut digesta	2016	Australia	*Salmo salar*	~
Aliivibrio finisterrensis_A	A33	SEZQ00000000	97	4123830	39.03	hind gut digesta	2016	Australia	*Salmo salar*	~
Aliivibrio finisterrensis_A	A4	SEZR00000000	105	4018091	39.04	hind gut digesta	2016	Australia	*Salmo salar*	~
Aliivibrio finisterrensis_A	A2	SEZS00000000	115	4140759	39.03	hind gut digesta	2016	Australia	*Salmo salar*	~
Aliivibrio finisterrensis_A	A9	SEZP00000000	122	4393741	38.85	hind gut digesta	2016	Australia	*Salmo salar*	179729
Aliivibrio finisterrensis_A	A21	SEZN00000000	129	4394502	38.85	hind gut digesta	2016	Australia	*Salmo salar*	179102
Aliivibrio finisterrensis_A	A15	SEZO00000000	163	4266806	38.91	hind gut digesta	2016	Australia	*Salmo salar*	~
Aliivibrio finisterrensis_A	A26	SEZM00000000	168	4262285	38.91	hind gut digesta	2016	Australia	*Salmo salar*	~
Aliivibrio finisterrensis_A	A37	SEZL00000000	170	4264469	38.91	hind gut digesta	2016	Australia	*Salmo salar*	~
Aliivibrio finisterrensis_A	A46	SEZK00000000	173	4228214	38.90	hind gut digesta	2016	Australia	*Salmo salar*	~

Aliivibrio finisterrensis_A	A6	JARACR000000000	177	4174510	38.89	gastrointestinal mucosa	2017	Australia	*Salmo salar*	~
Aliivibrio fischeri	DSM 507	CP092712,CP092713	2	4503206	38.32	seawater	1940	USA	~	~
Aliivibrio fischeri	MJ11	CP001133,CP001139,CP001134	3	4503336	38.20	pineapplefish	1994	Japan	*Monocentris japonica*	179459
Aliivibrio fischeri	ENK5	JBLOOV000000000	11	4750424	38.43	great white shark cloaca	2023	Australia	*Carcharodon carcharias*	~
Aliivibrio fischeri	etasm.1.1	WOBA00000000	41	4228612	38.09	light organ	2000	Australia	*Euprymna tasmanica*	~
Aliivibrio fischeri	5LC	CVOI00000000	54	4250496	38.29	gilthead bream	2013		*Sparus aurata*	~
Aliivibrio fischeri	emors.6.1	WOBI00000000	55	4203219	38.20	squid light organ	1995	Japan	*Euprymna morsei*	~
Aliivibrio fischeri	emors.6.2	WOBH00000000	56	4202714	38.19	squid light organ	1995	Japan	*Euprymna morsei*	~
Aliivibrio fischeri	emors.8.7	WOBD00000000	73	4310914	38.25	squid light organ	1995	Japan	*Euprymna morsei*	~
Aliivibrio fischeri	SR5	CM001400.1,CM001401.1	75	4272414	38.40	squid light organ	1995	Mediterranean Sea	*Sepiola robusta*	~
Aliivibrio fischeri	lpeal.1.1	WOAY00000000	78	4266928	38.32	squid light organ	1989	USA	*Doryteuthis pealeii*	~
Aliivibrio fischeri	5F33	MAJO00000000	81	4100707	38.27	seawater	2006	USA	~	~
Aliivibrio fischeri	5F97	MAJN00000000	83	4343102	38.35	seawater	2006	USA	~	~
Aliivibrio fischeri	lpeal.1.2	WOAX00000000	87	4260855	38.31	squid light organ	1989	USA	*Doryteuthis pealeii*	~
Aliivibrio fischeri	5F306	MAJP00000000	93	4154990	38.28	seawater	2006	USA	~	~
Aliivibrio fischeri	ZF47	MAJY00000000	103	4242906	38.28	seawater	2006	USA	~	~
Aliivibrio fischeri	ZF25	MCGK00000000	118	4090141	38.30	seawater	2006	USA	~	~
Aliivibrio fischeri	5F7	MCGJ00000000	119	4317687	38.27	seawater	2006	USA	~	~
Aliivibrio fischeri	LB10LO6	JAJVEL000000000	126	4111116	38.34	Atlantic brief squid	2017	USA	*Lolliguncula brevis*	~
Aliivibrio fischeri	5F23	MAJQ00000000	128	4159023	38.26	seawater	2006	USA	~	~
Aliivibrio fischeri	ZF73	MAJX00000000	128	4153668	38.30	seawater	2006	USA	~	~
Aliivibrio fischeri	5F146	MAJR00000000	149	4074230	38.30	seawater	2006	USA	~	~
Aliivibrio fischeri	10N.261.55. A3	JBFRMZ000000000	153	4114535	38.33	seawater	2010	USA	~	~
Aliivibrio fischeri	FF227	MAJW00000000	178	4246565	38.36	seawater	2006	USA	~	~
Aliivibrio fischeri	S10_S31	JAAUWR000000000	192	4349486	38.38	coastal water	2017	Denmark	~	~
Aliivibrio fischeri	ZF-211	AJYI00000000	197	4037340	38.30	seawater	2006	USA	~	~
Aliivibrio fischeri_A	HQB-91	JBHZSK000000000	36	4282080	38.22	euryhaline nematode	2019	China	*Litoditis marina*	~
Aliivibrio fischeri_A	emors.1.2	WOBW00000000	44	4243833	38.27	squid light organ	1995	Japan	*Euprymna morsei*	~
Aliivibrio fischeri_A	emors.2.2	WOBQ00000000	45	4243496	38.27	squid light organ	1995	Japan	*Euprymna morsei*	~
Aliivibrio fischeri_A	emors.5.1t	WOBK00000000	49	4200693	38.25	squid light organ	1995	Japan	*Euprymna morsei*	~
Aliivibrio fischeri_A	emors.5.1o	WOBL00000000	61	4204818	38.27	squid light organ	1995	Japan	*Euprymna morsei*	2495, 1727
Aliivibrio fischeri_A	emors.5.2	WOBJ00000000	91	4337347	38.25	squid light organ	1995	Japan	*Euprymna morsei*	~

Aliivibrio fischeri_B	EM17	JBUYRU000000000	2	4406490	38.47	squid light organ	1998	Japan	*Euprymna morsei*	~
Aliivibrio fischeri_B	EM30	JBUYRT000000000	2	4285186	38.35	squid light organ	1994	Japan	*Euprymna morsei*	~
Aliivibrio fischeri_B	KB4B5	JBUYRS000000000	2	4243277	38.37	squid light organ	2005	USA	*Euprymna scolopes*	~
Aliivibrio fischeri_B	MB14A5	JBQAPA000000000	2	4150073	38.43	squid light organ	2005	USA	*Euprymna scolopes*	~
Aliivibrio fischeri_B	MJ12	JBUYRR000000000	2	4178746	38.42	fish light organ	1994	Japan	*Monocentris japonica*	~
Aliivibrio fischeri_B	mjapo.8.1	JBUYRQ000000000	2	4209253	38.40	fish light organ	2005	Japan	*Monocentris japonica*	~
Aliivibrio fischeri_B	SA1	JBUYRP000000000	2	4365748	38.38	squid light organ	1998	France	*Sepiola affinis*	~
Aliivibrio fischeri_B	CG101	JBUYRV000000000	3	4133750	38.50	fish light organ	1998	Australia	*Cleidopus gloriamaris*	~
Aliivibrio fischeri_B	ES114	CP000020,CP000021,CP000022	3	4273718	38.30	squid light organ	1989	USA	*Euprymna scolopes*	45489
Aliivibrio fischeri_B	KB1A98	JBQAOV000000000	3	4289549	38.39	squid light organ	2005	USA	*Euprymna scolopes*	~
Aliivibrio fischeri_B	KB2B1	JBQAOW000000000	3	4479127	38.35	squid light organ	2005	USA	*Euprymna scolopes*	1886
Aliivibrio fischeri_B	MB14A3	JBQAOZ000000000	3	4215028	38.40	squid light organ	2005	USA	*Euprymna scolopes*	~
Aliivibrio fischeri_B	BW9	JBTYYO00000000	4	4795462	38.00	squid light organ	2023	USA	*Euprymna berryi*	167136, 47639
Aliivibrio fischeri_B	FQ-A001	SJSX00000000	4	4286900	38.26	squid light organ	2015	USA	*Euprymna scolopes*	~
Aliivibrio fischeri_B	MB11B1	JBQAOX000000000	4	4729286	38.21	squid light organ	2005	USA	*Euprymna scolopes*	167498, 8026
Aliivibrio fischeri_B	MB13B2	JBQAOY000000000	4	4480537	38.36	squid light organ	2005	USA	*Euprymna scolopes*	23019, 4901
Aliivibrio fischeri_B	ES213	JBQAOU000000000	5	4572427	38.36	squid light organ	1989	USA	*Euprymna scolopes*	51378, 4710
Aliivibrio fischeri_B	H905	JBCDWF000000000	5	4417355	38.46	seawater	1990	USA	~	~
Aliivibrio fischeri_B	ES401	SRJG00000000	6	4283732	38.31	squid light organ	1990	USA	*Euprymna scolopes*	~
Aliivibrio fischeri_B	ESF436_4	JAJVEF000000000	33	4130996	38.18	sand flat	2018	USA	*Euprymna scolopes*	~
Aliivibrio fischeri_B	MB15A5	LTZE00000000	33	4118676	38.21	squid light organ	2007	USA	*Euprymna scolopes*	~
Aliivibrio fischeri_B	ESF436_3	JAJVEE000000000	35	4129014	38.19	sand flat	2018	USA	*Euprymna scolopes*	~
Aliivibrio fischeri_B	ESF436_6	JAJVEH000000000	35	4128606	38.18	sand flat	2018	USA	*Euprymna scolopes*	~
Aliivibrio fischeri_B	VLS2	LTZF00000000	37	4092361	38.25	squid light organ	2007	USA	*Euprymna scolopes*	~
Aliivibrio fischeri_B	emors.11.2	WOBS00000000	40	4125930	38.21	squid light organ	1995	Japan	*Euprymna morsei*	~
Aliivibrio fischeri_B	ESM442_6	JAJVEJ000000000	41	4043186	38.20	sand flat	2018	USA	*Euprymna scolopes*	~
Aliivibrio fischeri_B	MB13B1	LTZA00000000	42	4309902	38.09	squid light organ	2007	USA	*Euprymna scolopes*	~
Aliivibrio fischeri_B	ESM442_5	JAJVEI000000000	44	4045866	38.21	sand flat	2018	USA	*Euprymna scolopes*	~
Aliivibrio fischeri_B	etasm.2.1	WOAZ00000000	44	4206316	38.18	squid light organ	2000	Australia	*Euprymna tasmanica*	~
Aliivibrio fischeri_B	emors.2.1	WOBR00000000	45	4228982	38.28	squid light organ	1995	Japan	*Euprymna morsei*	~
Aliivibrio fischeri_B	ESM429_3	JAJVEC000000000	45	4102582	38.26	sand flat	2017	USA	*Euprymna scolopes*	~
Aliivibrio fischeri_B	emors.9.2	WOBB00000000	46	4301235	38.18	squid light organ	1995	Japan	*Euprymna morsei*	~
Aliivibrio fischeri_B	ESM429_1	JAJVEB000000000	46	4105691	38.26	sand flat	2017	USA	*Euprymna scolopes*	~

Aliivibrio fischeri_B	MB15A4	LTYZ00000000	48	4201762	38.23	squid light organ	2007	USA	*Euprymna scolopes*	~
Aliivibrio fischeri_B	ESF436_5	JAJVEG000000000	50	4175983	38.25	sand flat	2018	USA	*Euprymna scolopes*	~
Aliivibrio fischeri_B	emors.3.1	WOBP00000000	51	4235862	38.22	squid light organ	1995	Japan	*Euprymna morsei*	~
Aliivibrio fischeri_B	emors.4.2	WOBM00000000	55	4212072	38.18	squid light organ	1995	Japan	*Euprymna morsei*	~
Aliivibrio fischeri_B	emors.3.2	WOBO00000000	59	4254142	38.22	squid light organ	1995	Japan	*Euprymna morsei*	~
Aliivibrio fischeri_B	emors.9.1	WOBC00000000	59	4472417	38.07	squid light organ	1995	Japan	*Euprymna morsei*	~
Aliivibrio fischeri_B	emors.1.1	WOBX00000000	60	4242687	38.22	squid light organ	1995	Japan	*Euprymna morsei*	~
Aliivibrio fischeri_B	emors.7.1	WOBG00000000	63	4251982	38.19	squid light organ	1995	Japan	*Euprymna morsei*	51188
Aliivibrio fischeri_B	MB13B3	LTZC00000000	72	4542584	38.05	squid light organ	2007	USA	*Euprymna scolopes*	168202
Aliivibrio fischeri_B	emors.11.1	WOBT00000000	75	4387723	38.15	squid light organ	1995	Japan	*Euprymna morsei*	~
Aliivibrio fischeri_B	ESM429_4	JAJVED000000000	77	4424884	38.18	sand flat	2017	USA	*Euprymna scolopes*	52361, 3323
Aliivibrio fischeri_B	emors.7.2	WOBF00000000	88	4177598	38.20	squid light organ	1995	Japan	*Euprymna morsei*	~
Aliivibrio fischeri_B	emors.4.1	WOBN00000000	116	4576484	38.08	squid light organ	1995	Japan	*Euprymna morsei*	~
Aliivibrio fischeri_B	emors.10.1	WOBV00000000	119	4308364	38.15	squid light organ	1995	Japan	*Euprymna morsei*	~
Aliivibrio fischeri_B	cmult.1.2	WOBY00000000	130	4103641	38.29	squid light organ	2006	Japan	*Coelorinchus multispinulosus*	~
Aliivibrio fischeri_B	cmult.1.1	WOBZ00000000	149	4105798	38.29	squid light organ	2006	Japan	*Coelorinchus multispinulosus*	~
Aliivibrio fischeri_B	emors.10.3	WOBU00000000	152	4302699	38.19	squid light organ	1995	Japan	*Euprymna morsei*	~
Aliivibrio fischeri_B	emors.8.2	WOBE00000000	158	4443451	38.08	squid light organ	1995	Japan	*Euprymna morsei*	167154
Aliivibrio fischeri_B	NBRC 101058	BJTZ00000000	176	4683933	38.22	octopus	1998	Japan	*Octopus vulgaris*	61671
Aliivibrio fischeri_B	VC2WE2	LYCF00000000	40	4218610	38.19	squid light organ	2003	USA	*unknown*	~
Aliivibrio logei	ATCC 35077	CM170656:CM170657	2	4770788	39.00	Tanner crab lesion	1978	USA	*Chionoecetes bairdi*	~
Aliivibrio logei	ATCC 29985	CM170658:CM170659	2	4521013	39.00	Arctic mussel gut	1978	USA	*Mytilus edulis*	~
Aliivibrio logei	1S159	MAJU00000000	76	4638003	38.97	seawater	2006	USA	~	~
Aliivibrio logei	5S-186	AJYJ00000000	162	4451230	39.00	seawater	2006	USA	~	~
Aliivibrio logei	SR45-2	JACJDQ000000000	227	4649514	39.13	seawater	2018	USA	~	~
Aliivibrio salmonicida	VS224	CP034240,CP034241,CP034238,CP 034239	4	4760779	38.82	Atlantic salmon	1987	Norway	*Salmo salar*	285684, 31858
Aliivibrio salmonicida	LFI1238	FM178379,FM178380,FM178382,FM 178384,FM178383,FM178381	6	4655660	38.97	Atlantic cod		Norway	*Gadus morhua*	83540, 30807, 5360, 4327
Aliivibrio salmonicida	SP1	JBREXB000000000	26	5127577	39.18	Atlantic salmon	2024	Canada	*Salmo salar*	~
Aliivibrio salmonicida	JAMM1233	JAKJUB000000000	169	4825530	38.98	marine sediment	2006	Japan	~	15153
Aliivibrio salmonicida	14-Bac1099	JAKZZP000000000	256	4249971	38.73	Atlantic salmon		Norway	*Salmo salar*	235995, 29089
Aliivibrio salmonicida	15-Vs224	JAKZZO000000000	260	4246637	38.72	Atlantic salmon		Norway	*Salmo salar*	236803, 29108, 5360

Aliivibrio salmonicida	12-BacKlon1	JAKZZR000000000	262	4178062	38.70	Atlantic salmon		Norway	*Salmo salar*	236725, 29109, 5360
Aliivibrio salmonicida	13-BacKlon4	JAKZZQ000000000	267	4172142	38.69	Atlantic salmon		Norway	*Salmo salar*	236598, 29101, 5360
Aliivibrio sifiae	NBRC 105001	MSCP00000000	8	4743237	38.25	seawater	2007	Japan	~	~
Aliivibrio sifiae	2025O3Ga	JBXBSD00000000	11	4912487	38.50	Pacific oyster	2025	Canada	*Crassostrea gigas*	~
Aliivibrio sifiae	CL3	LNRJ00000000	57	4567225	38.28	Coastal seawater	2016	Chile	~	~
Aliivibrio sifiae	CL1	LNRI00000000	71	4565725	38.27	Coastal seawater	2016	Chile	~	~
Aliivibrio sifiae	CL6	LNRM00000000	79	4660341	38.19	Coastal seawater	2016	Chile	~	~
Aliivibrio sifiae	CL4	LNRK00000000	83	4660786	38.22	Coastal seawater	2016	Chile	~	~
Aliivibrio sifiae	JAMM1683	JAKJTF000000000	88	4661563	38.25	marine sediment	2004	Japan	~	~
Aliivibrio sifiae	CL5	LNRL00000000	90	4663233	38.23	Coastal seawater	2016	Chile	~	~
Aliivibrio sifiae	CL7	LNRN00000000	105	4590040	38.22	Coastal seawater	2016	Chile	~	~
Aliivibrio sifiae_A	ATCC 33715	MSCO00000000	4	4462628	38.30	seawater, off California	ca 1976	USA	~	~
Aliivibrio sp.	Ust_30	JAAGZR000000000	788	4172194	38.92	sediemnt foram metagenome	2017	Peru	*Uvigerina striata*	~
Aliivibrio sp028765545	S4TY2	JARACX000000000	89	4373731	38.84	gastrointestinal mucosa	2018	Australia	*Salmo salar*	~
Aliivibrio sp028765545	S4MY1	JARACP000000000	89	4340179	38.84	gastrointestinal mucosa	2018	Australia	*Salmo salar*	~
Aliivibrio sp028765545	S4MY4	JARACW000000000	91	4373432	38.84	gastrointestinal mucosa	2018	Australia	*Salmo salar*	~
Aliivibrio sp028765545	S4MY2	JARACU000000000	91	4373838	38.84	gastrointestinal mucosa	2018	Australia	*Salmo salar*	~
Aliivibrio sp028765545	S4MY3	JARACV000000000	93	4373166	38.84	gastrointestinal mucosa	2018	Australia	*Salmo salar*	~
Aliivibrio sp028765545	S4TY1	JARACQ000000000	93	4372989	38.84	gastrointestinal mucosa	2018	Australia	*Salmo salar*	~
Aliivibrio sp028765575	S2MY1	JARACN000000000	159	3647736	39.05	gastrointestinal mucosa	2018	Australia	*Salmo salar*	~
Aliivibrio sp028765575	S3MY1	JARACO000000000	161	3803276	39.00	gastrointestinal mucosa	2018	Australia	*Salmo salar*	167236
Aliivibrio wodanis	NVI 06/09/139	LR721750-3	4	4972774	38.50	winter ulcer	2006	Norway	*Salmo salar*	194193, 79322
Aliivibrio wodanis	BL6	JBREXA000000000	11	5166846	38.50	Atlantic salmon	2024	Canada	*Salmo salar*	~
Aliivibrio wodanis	SP11	JBREXC000000000	11	5017375	38.90	Atlantic salmon	2024	Canada	*Salmo salar*	73983
Aliivibrio_A kagoshimensis	JAMM0721	JAKJSQ000000000	171	4349344	42.67	marine sediment	2004	Japan	~	~

Table S3. Details of intact integrons present in *Aliivibrio* strains including ARG presence.

Species	Strain	Intact integron genomic location	length (bp)	# CDS (excluding integrase)	ARGs	Notes
Aliivibrio salmonicida	Vs224	CP034238.1:4559-8600	4041	4	*dfrA1* (pseudogene)	integron integrase (pseudogene)
Aliivibrio salmonicida	Vs224	CP034238.1:273511-277553	4042	5	*dfrA1*	integron integrase (pseudogene)
Aliivibrio salmonicida	Vs224	CP034238.1:251297-255341	4044	5	*dfrA1*	~
Aliivibrio salmonicida	15-Vs224	JAKZZO010000118.1:6864-10908	4044	6	*dfrA1*	~
Aliivibrio salmonicida	13-BacKlon4	JAKZZQ010000117.1:6864-10908	4044	6	*dfrA1*	~
Aliivibrio salmonicida	12-BacKlon1	JAKZZR010000116.1:1000-5044	4044	5	*dfrA1*	~
Aliivibrio fischeri	MJ11	CP001133.1:747682-818288	70456	80	*APT** (2 copies)	gloA (2 copies), SDR family oxidoreductase, cysteine hydrolase family protein, speG, RM
Aliivibrio fischeri	VCW2E2	NZ_LTZF01000018.1:154995-158731	3736	3	*APT**	~
Aliivibrio fischeri_A	emors.5.1o	WOBL01000013.1:8753-12611	3858	4	*APT**	~
Aliivibrio fischeri_A	emors.5.1t	WOBK01000002.1:454353-457624	3271	3	*APT**	~
Aliivibrio fischeri_A	emors.5.2	WOBJ01000028.1:83455-85853	2398	1	*APT**	~
Aliivibrio "friggae"	SR9	NZ_CM170645.1:1029969-1038156	8187	8	~	aspartate/glutamate racemase family protein, alkD
Aliivibrio "friggae"	ATCC 15382	CM170638.1:1080642-1082794	2152	2	~	~
Aliivibrio "friggae"	EL58	NZ_OMPC01000001.1:1123734-1128361	4627	2	~	~
Aliivibrio "friggae"	SR9	NZ_CM170645.1:1029969-1033116	3147	1	~	~
Aliivibrio "friggae"	SR8	JBXXRG010000001.1:1028879-1032026	3147	1	~	~
Aliivibrio "friggae"	SR10	JBXXRI010000001.1:1029969-1033116	3147	1	~	~
Aliivibrio "friggae"	SA12	CM170651.1.1:83017-93006	9989	11	~	~
Aliivibrio finisterrensis	LMG 23869	WBVP01000025.1:22101-39430	17329	18	~	cdd, pseudogene rich
Aliivibrio finisterrensis_A	S2TY2	JARACS010000002:2854-29993	27139	24	~	lecithin retinol acyltransferase family protein, hpt, toll/interleukin-1 receptor domain-containing protein
Aliivibrio finisterrensis_A	S3TY1	JARACT010000002:2854-29993	27139	24	~	lecithin retinol acyltransferase family protein, hpt, toll/interleukin-1 receptor domain-containing protein
Aliivibrio fischeri	emors.6.2	WOBH01000012.1:8518-10358	1840	0	~	~
Aliivibrio fischeri	ES114	CP000021.2:727937-736001	8064	8	~	~
Aliivibrio fischeri	S10_S31	JAAUWR010000025.1:54909-58225	3316	3	~	~

Aliivibrio fischeri	SR5	CM001401.1:709571-712164	2593	3	~	~
Aliivibrio fischeri	5F97	MAJN01000045.1:38-3741	3703	3	~	~
Aliivibrio fischeri	KB1A98	LTYV01000031.1:66814-71187	4373	5	~	~
Aliivibrio fischeri	ZF25	MCGK01000024.1:123-3709	3586	3	~	~
Aliivibrio fischeri	emors.6.1	WOBI01000003.1:565020-568478	3458	4	~	~
Aliivibrio fischeri	MB15A5	LTZE01000012.1:59887-62472	2585	2	~	~
Aliivibrio fischeri	ES213	LTYU01000025.1:41025-47763	6738	10	~	~
Aliivibrio fischeri	VSL2	LCYF01000018.1:1-3644	3643	4	~	~
Aliivibrio fischeri	MB15A4	LTYZ01000024.1:113202-116471	3269	3	~	~
Aliivibrio fischeri	FF227	MAJW01000113.1:326-3275	2949	3	~	~
Aliivibrio fischeri	MB13B1	LTZA01000014.1:181931-188080	6149	3	~	class I SAM-dependent methyltransferase
Aliivibrio fischeri_B	emors.9.1	WOBC01000029.1:12-4860	4848	2	~	S-type pyocin T6SS effector
Aliivibrio fischeri_B	emors.3.2	WOBO01000005.1:614856-619285	4429	4	~	~
Aliivibrio fischeri_B	SA1	JBUYRP010000002.1:166469-172160	5691	6	~	class I SAM-dependent methyltransferase
Aliivibrio fischeri_B	ESF436_4	JAJVEF010000005.1:220344-225003	4659	4	~	~
Aliivibrio fischeri_B	ESF436_3	JAJVEE010000011.1:36722-41381	4659	4	~	~
Aliivibrio fischeri_B	ESM442_5	JAJVEI010000005.1:44864-51172	6308	7	~	~
Aliivibrio fischeri_B	MB14A5	JBQAPA010000002.1:302353-307819	5466	4	~	~
Aliivibrio fischeri_B	emors.1.1	WOBX01000001.1:2842-13071	10229	8	~	~
Aliivibrio fischeri_B	MB1B11	JBQAOX010000004.1:47890-51157	3267	2	~	~
Aliivibrio fischeri_B	etasm.2.1	WOAZ01000007.1:206469-210636	4167	3	~	~
Aliivibrio fischeri_B	ESM442_6	JAJVEJ010000003.1:209492-215800	6308	7	~	~
Aliivibrio fischeri_B	emors.11.2	WOBS01000001.1:384457-389940	5483	5	~	~
Aliivibrio fischeri_B	NBRC 101058	BJTZ01000001.1:535-5461	4926	5	~	~
Aliivibrio fischeri_B	emors.10.3	WOBU01000033.1:257237-262032	4795	3	~	~
Aliivibrio fischeri_B	mjapo.8.1	JBUYRQ010000002.1:1261253-1273446	12193	12	~	ribB, YgeA-like amino acid racemase, AAA family ATPase (2 copies)
Aliivibrio fischeri_B	CG101	JBUYRV010000003.1:48617-51062	2445	2	~	SIR2 family NAD-dependent protein deacylase, rdgB
Aliivibrio fischeri_B	MB11B1	JBQAOX010000004.1:47890-51157	3267	2	~	~
Aliivibrio fischeri_B	ESF436_6	JAJVEH010000010.1:94405-99064	4659	4	~	~
Aliivibrio logei	1S159	MAJU01000015.1:62-2897	2835	3	~	~
Aliivibrio logei	1S159	MAJU01000015.1:62-2897	2835	3	~	~

Aliivibrio logei	SR45-2	JACJDQ010000071.1:1103-4415	3312	2	~	trypsin-like peptidase domain-containing protein
Aliivibrio salmonicida	JAMM1233	JAKJUB010000035.1:601-5030	4429	2	~	~
Aliivibrio salmonicida	LFI1238	FM178380.1:593499-598810	5311	2	~	~
Aliivibrio salmonicida	Vs224	CP034241.1:593550-598861	5311	2	~	~
Aliivibrio sifiae	CL4	LNRK01000011.1:119-5381	5262	5	~	~
Aliivibrio sifiae	CL3	LNRJ01000012.1:63689-69259	5570	6	~	~
Aliivibrio sifiae	CL5	LNRL01000012.1:119-5381	5262	5	~	~
Aliivibrio sifiae	JAMM1683	JAKJTF010000039.1:56946-64314	7368	7	~	HAD hydrolase, gstA
Aliivibrio wodanis	NVI 06/09/139	LN554847.1:614731-620016	5285	4	~	~
Aliivibrio wodanis	NVI 06/09/139	LN554846.1:1117147-127096	9949	9	~	5'-nucleotidase
Aliivibrio wodanis	SP11	JBREXC010000009.1:133237-139790	6553	7	~	~
Aliivibrio wodanis	BL6	JBXXRA010000001.1:1088877-1094392	5515	6	~	~
Aliivibrio logei	ATCC 35077	ASAH01000025.1:339-5302	4963	4	~	RM
Aliivibrio wodanis	SP11	JBREXC010000009.1:123415-127115	3700	4	~	RES domain protein

Table S4. Details of CALIN present in *Aliivibrio* strains including ARG presence.

Species	Strain	CALIN region	length (bp)	# CDS	ARGs	Notes
Aliivibrio fischeri	5F146	MAJR01000145.1:13-1503	1490	1	*APT**	~
Aliivibrio fischeri	LB10LO6	JAJVEL010000022.1:130-8459	8329	9	*APT**	class I SAM-dependent methyltransferase
Aliivibrio fischeri_A	emors.5.1t	WOBK01000023.1:698-5246	4548	5	*APT**	nucleotidyltransferase family protein
Aliivibrio fischeri_B	emors.9.2	WOBB01000025.1:289-2398	2109	2	*APT**	~
Aliivibrio fischeri_B	MJ12	JBUYRR010000001.1:1026695-1031918	5223	8	*APT**	~
Aliivibrio sifiae	CL1	LNRI01000016.1:307-20463	20156	29	*APT**	Rho-binding antiterminator, MdaB family protein, nucleotidyltransferase family protein
Aliivibrio sifiae	CL3	LNRJ01000014.1:4329-12097	7768	12	*APT**	Rho-binding antiterminator, nucleotidyltransferase family protein
Aliivibrio fischeri	5F7	MCGJ01000039.1:103-2057	1954	3	*catB*	~
Aliivibrio salmonicida	SP1	JBREXB010000001.1:1330155-1345563	15408	7	*catB*	HNH endonuclease, P-loop NTPase fold protein
Aliivibrio wodanis	NVI 06/09/139	LN554847.1:853218-856216	2998	5	*catB*	~
Aliivibrio fischeri	VSL2	NZ_LCYF01000007.1:1913-4678	2765	3	*cpt*	~
Aliivibrio fischeri	SR5	CM001401.1:697687-703985	6298	8	*cpt*	NAD-dependent epimerase/dehydratase
Aliivibrio fischeri	VCW2E2	NZ_LTZF01000025.1:3309-6255	2946	4	*cpt*	ubiquitin-activating E1 family protein
Aliivibrio fischeri_A	emors.5.2	WOBJ01000057.1:203-3489	3286	4	*cpt*	~
Aliivibrio fischeri_B	emors.9.1	WOBC01000011.1:7078-18473	11395	14	*cpt*	GGCT-like domain protein, protein-threonine AMPylation (FIDO) domain protein, cold shock/excalibur domain-containing protein
Aliivibrio fischeri_B	emors.11.2	WOBS01000008.1:3241-22145	18904	20	*cpt*	SDR family oxidoreductase
Aliivibrio logei	1S159	MAJU01000023.1:258-10266	10008	15	*cpt*	~
Aliivibrio sifiae	CL6	LNRM01000023.1:1218-5922	4704	6	*cpt*	~
Aliivibrio sifiae	CL4	LNRK01000019.1:19290-27584	8294	10	*cpt*	cysteine hydrolase family protein, HAD hydrolase
Aliivibrio sifiae	CL5	LNRL01000018.1:19290-27584	8294	10	*cpt*	cysteine hydrolase family protein, HAD hydrolase
Aliivibrio wodanis	NVI 06/09/139	LN554847.1:802325-806044	3719	6	*cpt*	~
Aliivibrio "friggae"	ATCC 15382	CM170638.1:1086617-1092160	5543	6	~	~
Aliivibrio "friggae"	ATCC 15382	CM170638.1:1543509-1545074	1565	2	~	~
Aliivibrio "friggae"	EL58	NZ_OMPC01000009.1:465621-469665	4044	5	~	~
Aliivibrio "friggae"	EL58	NZ_OMPC01000005.1:6870-9846	2976	5	~	~
Aliivibrio "friggae"	EL58	NZ_OMPC01000005.1:826-1934	1108	2	~	~

Aliivibrio "friggae"	SA12	CM170651.1.1:71422-79008	7586	9	~	~
Aliivibrio "friggae"	SA12	CM170650.1.1:1540987-1545455	4468	3	~	~
Aliivibrio "friggae"	SA12	CM170651.1.1:54521-57179	2658	5	~	~
Aliivibrio "friggae"	SA12	CM170651.1.1:48934-50699	1765	3	~	~
Aliivibrio "friggae"	SR6	JBXXRF010000001.1:1516955-1522384	5429	6	~	~
Aliivibrio "thorii"	MdR7	JBXXRJ010000001.1:1213794-1220552	6758	9	~	~
Aliivibrio "thorii"	MdR7	JBXXRJ010000001.1:1197834-1201203	3369	5	~	~
Aliivibrio "thorii"	MdR7	JBXXRJ010000001.1:1205883-1209121	3238	4	~	~
Aliivibrio "thorii"	MdR7	JBXXRJ010000001.1:1169799-1172555	2756	3	~	~
Aliivibrio "thorii"	MdR7	JBXXRJ010000001.1:1102736-1104354	1618	3	~	~
Aliivibrio "thorii"	SA5	JBXXRC010000002.1:1111979-1115580	3601	6	~	~
Aliivibrio "thorii"	SA5	JBXXRC010000001.1:1426875-1429896	3021	5	~	~
Aliivibrio "thorii"	SA5	JBXXRC010000002.1:1159827-1161260	1433	2	~	~
Aliivibrio "thorii"	SA6	JBXXRD010000002.1:1111980-1115581	3601	6	~	~
Aliivibrio "thorii"	SA6	JBXXRD010000001.1:1426870-1429891	3021	5	~	~
Aliivibrio "thorii"	SA6	JBXXRD010000002.1:1159828-1161261	1433	2	~	~
Aliivibrio "vili"	1S128	MAJV01000174.1:13-2564	2551	5	~	~
Aliivibrio "vili"	1S128	MAJV01000114.1:859-2983	2124	3	~	~
Aliivibrio "vili"	1S128	MAJV01000118.1:2-1733	1731	3	~	~
Aliivibrio "vili"	1S128	MAJV01000105.1:51-1734	1683	3	~	~
Aliivibrio "vili"	1S128	MAJV01000174.1:13-2564	2551	5	~	~
Aliivibrio "vili"	1S128	MAJV01000114.1:859-2983	2124	3	~	~
Aliivibrio "vili"	1S128	MAJV01000118.1:2-1733	1731	3	~	~
Aliivibrio "vili"	1S128	MAJV01000105.1:51-1734	1683	3	~	~
Aliivibrio "vili"	1S165	MAJT01000043.1:3711-7996	4285	7	~	~
Aliivibrio "vili"	1S165	MAJT01000048.1:803-3172	2369	4	~	~
Aliivibrio "vili"	1S165	MAJT01000059.1:435-1141	706	0	~	~
Aliivibrio "vili"	1S175	MAJS01000030.1:113-3876	3763	6	~	~
Aliivibrio "vili"	1S175	MAJS01000002.1:828-3209	2381	4	~	~
Aliivibrio "vili"	1S175	MAJS01000029.1:137-1626	1489	2	~	~
Aliivibrio finisterrensis	LMG 23869	WBVP01000044.1:1-1113	1112	1	~	~
Aliivibrio finisterrensis	LMG 23869	WBVP01000051.1:36-999	963	3	~	~
Aliivibrio finisterrensis_A	A15	NZ_SEZO01000063:226-6563	6337	3	~	~
Aliivibrio finisterrensis_A	A15	NZ_SEZO01000072.1:3-4355	4352	4	~	~
Aliivibrio finisterrensis_A	A15	NZ_SEZO01000069.1:1334-5064	3730	4	~	~
Aliivibrio finisterrensis_A	A15	NZ_SEZO01000066:746-4108	3362	1	~	~

Aliivibrio finisterrensis_A	A15	NZ_SEZO01000076.1:171-2640	2469	3	~	~
Aliivibrio finisterrensis_A	A15	NZ_SEZO01000078.1:1290-3590	2300	3	~	~
Aliivibrio finisterrensis_A	A15	NZ_SEZO01000084.1:360-2056	1696	1	~	~
Aliivibrio finisterrensis_A	A15	NZ_SEZO01000108.1:23-1384	1361	2	~	~
Aliivibrio finisterrensis_A	A15	NZ_SEZO01000101.1:25-133.2	1307	1	~	~
Aliivibrio finisterrensis_A	A15	NZ_SEZO01000106.1:121-1420	1299	1	~	~
Aliivibrio finisterrensis_A	A15	NZ_SEZO01000085.1:1171-2416	1245	0	~	~
Aliivibrio finisterrensis_A	A15	NZ_SEZO0100067.1:4244-5325	1081	1	~	~
Aliivibrio finisterrensis_A	A15	NZ_SEZO0100114.1:189-1114	925	1	~	~
Aliivibrio finisterrensis_A	A15	NZ_SEZO0100122.1:3-874	871	1	~	~
Aliivibrio finisterrensis_A	A15	NZ_SEZO01000126:3-822	819	1	~	~
Aliivibrio finisterrensis_A	A15	NZ_SEZO01000103.1:728-1429	701	1	~	~
Aliivibrio finisterrensis_A	A15	NZ_SEZO01000096.1:701-1369	668	0	~	~
Aliivibrio finisterrensis_A	A2	NZ_SEZS01000039.1:1514-8456	6942	6	~	~
Aliivibrio finisterrensis_A	A2	NZ_SEZS01000051.1:113-3761	3648	4	~	~
Aliivibrio finisterrensis_A	A2	NZ_SEZS0100050.1:680-3548	2868	2	~	~
Aliivibrio finisterrensis_A	A2	NZ_SEZS0100053.1:681-2945	2264	5	~	~
Aliivibrio finisterrensis_A	A2	NZ_SEZS0100049.1:141-2364	2223	3	~	~
Aliivibrio finisterrensis_A	A2	NZ_SEZS0100063.1:164-1995	1831	2	~	~
Aliivibrio finisterrensis_A	A2	NZ_SEZS0100038.1:4117-5753	1636	2	~	~
Aliivibrio finisterrensis_A	A2	NZ_SEZS0100065.1:714-1906	1192	1	~	~
Aliivibrio finisterrensis_A	A2	NZ_SEZS01000058.1:1486-2595	1109	1	~	~
Aliivibrio finisterrensis_A	A2	NZ_SEZS0100084.1.43:3-873	870	1	~	~
Aliivibrio finisterrensis_A	A2	NZ_SEZS0100088.1:3-796	793	1	~	~
Aliivibrio finisterrensis_A	A21	NZ_SEZN01000063.1:172-3626	3454	7	~	~
Aliivibrio finisterrensis_A	A21	NZ_SEZN01000073.1:1287-4107	2820	3	~	~
Aliivibrio finisterrensis_A	A21	NZ_SEZN01000075.1:1334-3649	2315	3	~	~
Aliivibrio finisterrensis_A	A21	NZ_SEZN01000081.1:728-2633	1905	2	~	~
Aliivibrio finisterrensis_A	A21	NZ_SEZN01000093.1:247-1554	1307	1	~	~
Aliivibrio finisterrensis_A	A21	NZ_SEZN01000088.1:1001-2114	1113	1	~	~
Aliivibrio finisterrensis_A	A21	NZ_SEZN01000067.1:4244-5325	1081	1	~	~
Aliivibrio finisterrensis_A	A21	NZ_SEZN01000104:25-983	958	1	~	~
Aliivibrio finisterrensis_A	A21	NZ_SEZN01000074.1:25-870	845	3	~	~
Aliivibrio finisterrensis_A	A21	NZ_SEZN01000108.1:2-689	687	1	~	~
Aliivibrio finisterrensis_A	A26	NZ_SEZM01000061:226-6563	6337	3	~	~
Aliivibrio finisterrensis_A	A26	NZ_SEZM01000068.1:3-4355	4352	4	~	~

Aliivibrio finisterrensis_A	A26	NZ_SEZM01000067.1:323-4513	4190	5	~	~
Aliivibrio finisterrensis_A	A26	NZ_SEZM01000073.1:171-2640	2469	3	~	~
Aliivibrio finisterrensis_A	A26	NZ_SEZM01000072.1:1334-3649	2315	3	~	~
Aliivibrio finisterrensis_A	A26	NZ_SEZM01000074.1:1290-3590	2300	3	~	~
Aliivibrio finisterrensis_A	A26	NZ_SEZM01000079:946-2587	1641	1	~	~
Aliivibrio finisterrensis_A	A26	NZ_SEZM01000109.1:25-1386	1361	2	~	~
Aliivibrio finisterrensis_A	A26	NZ_SEZM01000099.1:25-1332	1307	1	~	~
Aliivibrio finisterrensis_A	A26	NZ_SEZM01000107.1:121-1420	1299	1	~	~
Aliivibrio finisterrensis_A	A26	NZ_SEZM01000083.1:1171-2416	1245	0	~	~
Aliivibrio finisterrensis_A	A26	NZ_SEZM01000100.1:281-1394	1113	1	~	~
Aliivibrio finisterrensis_A	A26	NZ_SEZM01000064.1:4244-5325	1081	1	~	~
Aliivibrio finisterrensis_A	A26	NZ_SEZM01000122.1:131-1089	958	1	~	~
Aliivibrio finisterrensis_A	A26	NZ_SEZM01000128.1:3-874	871	1	~	~
Aliivibrio finisterrensis_A	A26	NZ_SEZM01000131.1:3-822	819	1	~	~
Aliivibrio finisterrensis_A	A26	NZ_SEZM01000102.1:728-1429	701	1	~	~
Aliivibrio finisterrensis_A	A26	NZ_SEZM01000134.1:1-691	690	0	~	~
Aliivibrio finisterrensis_A	A26	NZ_SEZM01000091.1:25-708	683	0	~	~
Aliivibrio finisterrensis_A	A26	NZ_SEZM01000096.1:701-1369	668	0	~	~
Aliivibrio finisterrensis_A	A32	NZ_SEZJ01000026.1:10641-31246	20605	19	~	~
Aliivibrio finisterrensis_A	A32	NZ_SEZJ01000026.1:1165-5165	4000	4	~	~
Aliivibrio finisterrensis_A	A32	NZ_SEZJ01000007.1:97470-100613	3143	4	~	~
Aliivibrio finisterrensis_A	A32	NZ_SEZJ01000036.1:99-2801	2702	5	~	~
Aliivibrio finisterrensis_A	A32	NZ_SEZJ01000040.1:3-2511	2508	2	~	~
Aliivibrio finisterrensis_A	A32	NZ_SEZJ01000035.1:780-2658	1878	2	~	~
Aliivibrio finisterrensis_A	A32	NZ_SEZJ01000042.1:156-1849	1693	2	~	~
Aliivibrio finisterrensis_A	A32	NZ_SEZJ01000038.1:1011-2377	1366	4	~	~
Aliivibrio finisterrensis_A	A33	NZ_SEZQ01000038.1:2074-7600	5526	8	~	~
Aliivibrio finisterrensis_A	A33	NZ_SEZQ01000040.1:2180-6326	4146	3	~	~
Aliivibrio finisterrensis_A	A33	NZ_SEZQ01000044.1:130-3778	3648	3	~	~
Aliivibrio finisterrensis_A	A33	NZ_SEZQ0100046.1:194-3533	3339	2	~	~
Aliivibrio finisterrensis_A	A33	NZ_SEZQ0100045:680-3548	2868	2	~	~
Aliivibrio finisterrensis_A	A33	NZ_SEZQ0100048.1:914-3254	2340	3	~	~
Aliivibrio finisterrensis_A	A33	NZ_SEZQ0100035.1:4117-5753	1636	2	~	~
Aliivibrio finisterrensis_A	A33	NZ_SEZQ0100057.1:714-1906	1192	1	~	~
Aliivibrio finisterrensis_A	A33	NZ_SEQ01000052.1:1486-2595	1109	1	~	~
Aliivibrio finisterrensis_A	A33	NZ_SEZQ0100075.1:3-873	870	1	~	~

Aliivibrio finisterrensis_A	A33	NZ_SEZS0100079.1:3-796	793	1	~	~
Aliivibrio finisterrensis_A	A37	NZ_SEZL01000065:226-6563	6337	3	~	~
Aliivibrio finisterrensis_A	A37	NZ_SEZL01000072.1:3-4355	4352	4	~	~
Aliivibrio finisterrensis_A	A37	NZ_SEZL01000071.1:1334-5064	3730	4	~	~
Aliivibrio finisterrensis_A	A37	NZ_SEZL01000068:717-4079	3362	1	~	~
Aliivibrio finisterrensis_A	A37	NZ_SEZL01000076.1:171-2640	2469	3	~	~
Aliivibrio finisterrensis_A	A37	NZ_SEZL01000077.1:1290-3590	2300	3	~	~
Aliivibrio finisterrensis_A	A37	NZ_SEZL01000080.1:1001-2697	1696	1	~	~
Aliivibrio finisterrensis_A	A37	NZ_SEZL01000109.1:23-1384	1361	2	~	~
Aliivibrio finisterrensis_A	A37	NZ_SEZL01000101.1:25-1332	1307	1	~	~
Aliivibrio finisterrensis_A	A37	NZ_SEZL01000106.1:121-1426	1305	1	~	~
Aliivibrio finisterrensis_A	A37	NZ_SEZL01000085.1:1171-2416	1245	0	~	~
Aliivibrio finisterrensis_A	A37	NZ_SEZL01000069.1:4244-5325	1081	1	~	~
Aliivibrio finisterrensis_A	A37	NZ_SEZL01000120.1:3-874	871	1	~	~
Aliivibrio finisterrensis_A	A37	NZ_SEZL01000125.1:3-822	819	1	~	~
Aliivibrio finisterrensis_A	A37	NZ_SEZL01000103.1:728-1469	741	1	~	~
Aliivibrio finisterrensis_A	A37	NZ_SEZL01000129.1:1-691	690	0	~	~
Aliivibrio finisterrensis_A	A37	NZ_SEZL01000096.1:701-1369	668	0	~	~
Aliivibrio finisterrensis_A	A4	NZ_SEZR0100045.1:194-3533	3339	2	~	~
Aliivibrio finisterrensis_A	A4	NZ_SEZS0100041.1:141-3232	3091	3	~	~
Aliivibrio finisterrensis_A	A4	NZ_SEZR0100044.1:680-3548	2868	2	~	~
Aliivibrio finisterrensis_A	A4	NZ_SEZR0100048.1:914-3254	2340	3	~	~
Aliivibrio finisterrensis_A	A4	NZ_SEZR0100049.1:681-2945	2264	5	~	~
Aliivibrio finisterrensis_A	A4	NZ_SEZR0100043.1:141-2364	2223	3	~	~
Aliivibrio finisterrensis_A	A4	NZ_SEZR0100040.1:1514-3369	1855	2	~	~
Aliivibrio finisterrensis_A	A4	NZ_SEZR0100058.1:164-1995	1831	2	~	~
Aliivibrio finisterrensis_A	A4	NZ_SEZR0100054.1:113-1630	1517	1	~	~
Aliivibrio finisterrensis_A	A4	NZ_SEZR0100035.1:4117-5353	1236	2	~	~
Aliivibrio finisterrensis_A	A4	NZ_SEZR0100061.1:714-1906	1192	1	~	~
Aliivibrio finisterrensis_A	A4	NZ_SEZR01000055.1:1486-2595	1109	1	~	~
Aliivibrio finisterrensis_A	A4	NZ_SEZR0100078.1:3-873	870	1	~	~
Aliivibrio finisterrensis_A	A4	NZ_SEZR0100083.1:3-796	793	1	~	~
Aliivibrio finisterrensis_A	A4	NZ_SEZR0100084:130-890	760	0	~	~
Aliivibrio finisterrensis_A	A46	NZ_SEZK01000065:226-6563	6337	3	~	~
Aliivibrio finisterrensis_A	A46	NZ_SEZK01000072.1:104-4341	4237	4	~	~
Aliivibrio finisterrensis_A	A46	NZ_SEZK01000071.1:323-4513	4190	5	~	~

Aliivibrio finisterrensis_A	A46	NZ_SEZK01000075.1:1287-4107	2820	3	~	~
Aliivibrio finisterrensis_A	A46	NZ_SEZK01000077.1:171-2640	2469	3	~	~
Aliivibrio finisterrensis_A	A46	NZ_SEZL01000076.1:1334-3649	2315	3	~	~
Aliivibrio finisterrensis_A	A46	NZ_SEZK01000086.1:159-2049	1890	4	~	~
Aliivibrio finisterrensis_A	A46	NZ_SEZL01000081.1:946-2587	1641	1	~	~
Aliivibrio finisterrensis_A	A46	NZ_SEZK01000113.1:25-1386	1361	2	~	~
Aliivibrio finisterrensis_A	A46	NZ_SEZK01000102.1:25-1332	1307	1	~	~
Aliivibrio finisterrensis_A	A46	NZ_SEZK01000110.1:121-1426	1305	1	~	~
Aliivibrio finisterrensis_A	A46	NZ_SEZL01000084.1:1171-2416	1245	0	~	~
Aliivibrio finisterrensis_A	A46	NZ_SEZL01000100.1:360-1473	1113	1	~	~
Aliivibrio finisterrensis_A	A46	NZ_SEZL01000068.1:4244-5325	1081	1	~	~
Aliivibrio finisterrensis_A	A46	NZ_SEZL01000129.1:25-983	958	1	~	~
Aliivibrio finisterrensis_A	A46	NZ_SEZL01000130.1:3-874	871	1	~	~
Aliivibrio finisterrensis_A	A46	NZ_SEZK01000107.1:728-1429	701	1	~	~
Aliivibrio finisterrensis_A	A46	NZ_SEZK01000137.1:1-691	690	0	~	~
Aliivibrio finisterrensis_A	A46	NZ_SEZK01000092.1:1456-2139	683	0	~	~
Aliivibrio finisterrensis_A	A46	NZ_SEZK01000097.1:701-1369	668	0	~	~
Aliivibrio finisterrensis_A	A6	JARACR010000054.1:99-11411	11312	9	~	~
Aliivibrio finisterrensis_A	A6	JARACR010000056.1:3678-9329	5651	7	~	~
Aliivibrio finisterrensis_A	A6	JARACR010000065.1:50-2519	2469	3	~	~
Aliivibrio finisterrensis_A	A6	JARACR010000069.1:395-1993	1598	2	~	~
Aliivibrio finisterrensis_A	A6	JARACR010000072.1:2-1233	1231	2	~	~
Aliivibrio finisterrensis_A	A6	JARACR010000089.1:3-1171	1168	1	~	~
Aliivibrio finisterrensis_A	A6	JARACR010000079.1:460-1128	668	0	~	~
Aliivibrio finisterrensis_A	A9	NZ_SEZP01000059.1:3-3388	3385	6	~	~
Aliivibrio finisterrensis_A	A9	NZ_SEZP01000070.1:1287-4107	2820	3	~	~
Aliivibrio finisterrensis_A	A9	NZ_SEZP01000078.1:728-2633	1905	2	~	~
Aliivibrio finisterrensis_A	A9	NZ_SEZP01000071.1:2264-4018	1754	3	~	~
Aliivibrio finisterrensis_A	A9	NZ_SEZP01000073.1:1000-2696	1696	1	~	~
Aliivibrio finisterrensis_A	A9	NZ_SEZP01000087.1:247-1554	1307	1	~	~
Aliivibrio finisterrensis_A	A9	NZ_SEZP01000063.1:4244-5325	1081	1	~	~
Aliivibrio finisterrensis_A	A9	NZ_SEZP01000097.1:3-874	871	1	~	~
Aliivibrio finisterrensis_A	A9	NZ_SEZP01000065.1:564-1334	770	2	~	~
Aliivibrio finisterrensis_A	A9	NZ_SEZP01000066.1:587-1255	668	0	~	~
Aliivibrio finisterrensis_A	S2TY2	JARACS010000001.1:715408-718551	3143	4	~	~
Aliivibrio finisterrensis_A	S2TY2	JARACS010000021.1:29664-31357	1693	2	~	~

Aliivibrio finisterrensis_A	S2TY2	JARACS010000022.1:24503-26193	1690	2	~	~
Aliivibrio finisterrensis_A	S2TY2	JARACS010000027.1:945-2445	1500	2	~	~
Aliivibrio finisterrensis_A	S2TY2	JARACS010000026.1:972-2338	1366	4	~	~
Aliivibrio finisterrensis_A	S3TY1	JARACT010000001.1:715408-718551	3143	4	~	~
Aliivibrio finisterrensis_A	S3TY1	JARACT010000022.1:373-2783	2410	3	~	~
Aliivibrio finisterrensis_A	S3TY1	JARACT010000021.1:574-2267	1693	2	~	~
Aliivibrio finisterrensis_A	S3TY1	JARACT010000027.1:945-2445	1500	2	~	~
Aliivibrio finisterrensis_A	S3TY1	JARACT010000026.1:884-2250	1366	4	~	~
Aliivibrio fischeri	10N.261.55.A3	JBFRMZ010000050.1:61-2288	2227	3	~	~
Aliivibrio fischeri	10N.261.55.A3	JBFRMZ010000027.1:354-2374	2020	3	~	~
Aliivibrio fischeri	10N.261.55.A3	JBFRMZ010000026.1:2881-4449	1568	3	~	~
Aliivibrio fischeri	5F146	MAJR01000143.1:26-5605	5579	8	~	~
Aliivibrio fischeri	5F146	MAJR01000124.1:41-1803	1762	3	~	~
Aliivibrio fischeri	5F146	MAJR01000106.1:690-2325	1635	3	~	~
Aliivibrio fischeri	5F23	MAJQ01000054.1:429-4411	3982	5	~	~
Aliivibrio fischeri	5F23	MAJQ01000084.1:125-3155	3030	5	~	~
Aliivibrio fischeri	5F23	MAJQ01000077.1:468-3462	2994	2	~	~
Aliivibrio fischeri	5F23	MAJQ01000056.1:65210-67843	2633	3	~	~
Aliivibrio fischeri	5F23	MAJQ01000072.1:1-2323	2322	3	~	~
Aliivibrio fischeri	5F23	MAJQ01000116.1:167-2041	1874	3	~	~
Aliivibrio fischeri	5F23	MAJQ01000057.1:3354-4107	753	1	~	~
Aliivibrio fischeri	5F306	MAJP01000030.1:21-3790	3769	3	~	~
Aliivibrio fischeri	5F306	MAJP01000051.1:134-2603	2469	3	~	~
Aliivibrio fischeri	5F306	MAJP01000024.1:3481-5565	2084	4	~	~
Aliivibrio fischeri	5F306	MAJP01000055.1:188-2052	1864	3	~	~
Aliivibrio fischeri	5F306	MAJP01000062.1:22-1177	1155	2	~	~
Aliivibrio fischeri	5F306	MAJP01000043.1:36-1047	1011	1	~	~
Aliivibrio fischeri	5F33	MAJO01000032.1:36-3523	3487	2	~	~
Aliivibrio fischeri	5F33	MAJO01000038.1:1374-3623	2249	3	~	~
Aliivibrio fischeri	5F7	MCGJ01000039.1:5847-13832	7985	9	~	~
Aliivibrio fischeri	5F7	MCGJ01000041.1:1116-3944	2828	4	~	~
Aliivibrio fischeri	5F7	MCGJ01000086.1:419-1734	1315	1	~	~
Aliivibrio fischeri	5F97	MAJN01000026.1:1172-7233	6061	10	~	~
Aliivibrio fischeri	5F97	MAJN01000048.1:312-5031	4719	5	~	~
Aliivibrio fischeri	5F97	MAJN01000023.1:253101-257630	4529	4	~	~
Aliivibrio fischeri	5F97	MAJN01000025.1:971-2504	1533	2	~	~

Aliivibrio fischeri	5F97	MAJN01000017.1:2306-3834	1528	4	~	~
Aliivibrio fischeri	5F97	MAJN01000028.1:1276-2632	1356	1	~	~
Aliivibrio fischeri	5LC	NZ_CVOI01000032.1:138-2275	2137	3	~	~
Aliivibrio fischeri	5LC	NZ_CVOI01000033.1:580-1935	1355	1	~	~
Aliivibrio fischeri	DSM 507	NZ_CP092713.1:972681-976888	4207	5	~	~
Aliivibrio fischeri	DSM 507	NZ_CP092713.1:991763-993951	2188	4	~	~
Aliivibrio fischeri	DSM 507	NZ_CP092713.1:1019602-1020918	1316	3	~	~
Aliivibrio fischeri	DSM 507	NZ_CP092713.1:1061240-1062485	1245	0	~	~
Aliivibrio fischeri	DSM 507	NZ_CP092713.1:980772-981947	1175	1	~	~
Aliivibrio fischeri	DSM 507	NZ_CP092713.1:1072121-1073197	1076	2	~	~
Aliivibrio fischeri	emors.6.1	WOBI01000034.1:332-5225	4893	6	~	~
Aliivibrio fischeri	emors.6.1	WOBI01000036.1:273-2546	2273	3	~	~
Aliivibrio fischeri	emors.6.2	WOBH01000029.1:27-4974	4947	6	~	~
Aliivibrio fischeri	emors.6.2	WOBH01000032.1:1391-3285	1894	2	~	~
Aliivibrio fischeri	ES213	NZ_LTYU01000063.1:34-2747	2713	3	~	~
Aliivibrio fischeri	FF227	MAJW01000092.1:77540-81245	3705	6	~	~
Aliivibrio fischeri	KB1A98	NZ_LTYV01000009.1:2647-6619	3972	7	~	~
Aliivibrio fischeri	KB1A98	NZ_LTYV01000013.1:2141-5071	2930	6	~	~
Aliivibrio fischeri	KB1A98	NZ_LTYV01000045.1:3674-5351	1677	3	~	~
Aliivibrio fischeri	KB2B1	NZ_LTYW01000017.1:1587-13497	11910	21	~	~
Aliivibrio fischeri	KB4B5	NZ_LTZD01000011.1:16402:25276	8874	11	~	~
Aliivibrio fischeri	KB4B5	NZ_LTZD01000011.1:7681:12154	4473	6	~	~
Aliivibrio fischeri	KB4B5	NZ_LTZD01000015.1:191020-194746	3726	4	~	~
Aliivibrio fischeri	KB4B5	NZ_LTZD01000015.1:534169-536011	1842	3	~	~
Aliivibrio fischeri	LB10LO6	JAJVEL010000039.1:20-1292	1272	2	~	~
Aliivibrio fischeri	LB10LO6	JAJVEL010000029.1:1244-2021	777	1	~	~
Aliivibrio fischeri	lpeal.1.1	WOAY01000024.1:6-2371	2365	4	~	~
Aliivibrio fischeri	lpeal.1.1	WOAY01000057.1:479-1709	1230	1	~	~
Aliivibrio fischeri	MB13B1	NZ_LTZA01000006.1:5685-14889	9204	9	~	~
Aliivibrio fischeri	MB13B2	NZ_LTZB01000030.1:115-2828	2713	3	~	~
Aliivibrio fischeri	MB13B3	NZ_LTZC01000070.1:115-2828	2713	3	~	~
Aliivibrio fischeri	MB14A3	NZ_LTYX01000006.1:2751-4674	1923	2	~	~
Aliivibrio fischeri	MB14A3	NZ_LTYX01000001.1:27-1777	1750	3	~	~
Aliivibrio fischeri	MB15A4	NZ_LTYZ01000031.1:479-12388	11909	14	~	~
Aliivibrio fischeri	MB15A5	NZ_LTZE01000016.1:2925-3979	1054	1	~	~
Aliivibrio fischeri	S10_S31	JAAUWR010000036.1:5364-8903	3539	3	~	~

Aliivibrio fischeri	S10_S31	JAAUWR010000045.1:239-3577	3338	4	~	~
Aliivibrio fischeri	S10_S31	JAAUWR010000043.1:1438-3711	2273	2	~	~
Aliivibrio fischeri	S10_S31	JAAUWR010000041.1:1594-3692	2098	3	~	~
Aliivibrio fischeri	S10_S31	JAAUWR010000049.1:1-1468	1467	2	~	~
Aliivibrio fischeri	S10_S31	JAAUWR010000054.1:3-1151	1148	2	~	~
Aliivibrio fischeri	S10_S31	JAAUWR010000042.1:638-1322	684	0	~	~
Aliivibrio fischeri	S10_S31	JAAUWR010000047.1:1-684	683	0	~	~
Aliivibrio fischeri	SR5	AHIH01000015.1:27850-29833	1983	3	~	~
Aliivibrio fischeri	SR5	AHIH01000013.1:254-1562	1308	2	~	~
Aliivibrio fischeri	SR5	AHIH01000016.1:2608-3894	1286	3	~	~
Aliivibrio fischeri	SR5	AHIH01000042.1:456-1739	1283	0	~	~
Aliivibrio fischeri	VCW2E2	NZ_LTZF01000024.1:131017-133231	2214	4	~	~
Aliivibrio fischeri	ZF25	MCGK01000030.1:190675-195015	4340	5	~	~
Aliivibrio fischeri	ZF25	MCGK01000072.1:114-4242	4128	5	~	~
Aliivibrio fischeri	ZF25	MCGK01000053.1:1316-3253	1937	5	~	~
Aliivibrio fischeri	ZF25	MCGK01000085.1:109-1867	1758	2	~	~
Aliivibrio fischeri	ZF47	MAJY01000053.1:190-4578	4388	5	~	~
Aliivibrio fischeri	ZF47	MAJY01000003.1:803-3614	2811	4	~	~
Aliivibrio fischeri	ZF47	MAJY01000001.1:84260-86893	2633	3	~	~
Aliivibrio fischeri	ZF47	MAJY01000033.1:2842-4180	1338	1	~	~
Aliivibrio fischeri	ZF47	MAJY01000070.1:459-1691	1232	1	~	~
Aliivibrio fischeri	ZF47	MAJY01000083.1:1-611	610	2	~	~
Aliivibrio fischeri	ZF73	MAJX01000057.1:1876-4251	2375	5	~	~
Aliivibrio fischeri	ZF73	MAJX01000118.1:470-2153	1683	3	~	~
Aliivibrio fischeri	ZF73	MAJX01000113.1:3-1182	1179	1	~	~
Aliivibrio fischeri_A	emors.1.2	WOBW01000011.1:161481-171518	10037	11	~	~
Aliivibrio fischeri_A	emors.1.2	WOBW01000010.1:558651-564340	5689	5	~	~
Aliivibrio fischeri_A	emors.1.2	WOBW01000021.1:1159-4168	3009	4	~	~
Aliivibrio fischeri_A	emors.1.2	WOBW01000011.1:175816-177983	2167	2	~	~
Aliivibrio fischeri_A	emors.2.2	WOBQ01000012.1:11479-21516	10037	11	~	~
Aliivibrio fischeri_A	emors.2.2	WOBQ01000006.1:814-6503	5689	5	~	~
Aliivibrio fischeri_A	emors.2.2	WOBQ01000011.1:1158-4167	3009	4	~	~
Aliivibrio fischeri_A	emors.2.2	WOBQ01000012.1:5014-7181	2167	2	~	~
Aliivibrio fischeri_A	emors.5.1o	WOBL01000036.1:125-3475	3350	5	~	~
Aliivibrio fischeri_A	emors.5.1o	WOBL01000013.1:1311-4081	2770	4	~	~
Aliivibrio fischeri_A	emors.5.1o	WOBL01000012.1:7556-10144	2588	3	~	~

Aliivibrio fischeri_A	emors.5.1o	WOBL01000012.1:1705-3568	1863	3	~	~
Aliivibrio fischeri_A	emors.5.1t	WOBK01000025.1:126-3476	3350	5	~	~
Aliivibrio fischeri_A	emors.5.1t	WOBK01000002.1:462883-465653	2770	4	~	~
Aliivibrio fischeri_A	emors.5.1t	WOBK01000002.1:475169-477757	2588	3	~	~
Aliivibrio fischeri_A	emors.5.1t	WOBK01000002.1:469318-471181	1863	3	~	~
Aliivibrio fischeri_A	emors.5.2	WOBJ01000055.1:327-7548	7221	7	~	~
Aliivibrio fischeri_A	emors.5.2	WOBJ01000031.1:735-5097	4362	3	~	~
Aliivibrio fischeri_A	emors.5.2	WOBJ01000033.1:2459-6728	4269	7	~	~
Aliivibrio fischeri_A	emors.5.2	WOBJ01000042.1:2265-4395	2130	2	~	~
Aliivibrio fischeri_A	emors.5.2	WOBJ01000064.1:464-2044	1580	1	~	~
Aliivibrio fischeri_A	HQB-91	NZ_JBHZSK010000001.1:662731-667476	4745	4	~	~
Aliivibrio fischeri_A	HQB-91	NZ_JBHZSK010000021.1:68-1098	1030	1	~	~
Aliivibrio fischeri_A	lpeal.1.2	WOAX01000031.1:6-2371	2365	4	~	~
Aliivibrio fischeri_B	CG101	JBUYRV010000003.1:30479-36395	5916	11	~	~
Aliivibrio fischeri_B	CG101	JBUYRV010000003.1:7516-10642	3126	4	~	~
Aliivibrio fischeri_B	CG101	JBUYRV010000003.1:16680-19279	2599	0	~	~
Aliivibrio fischeri_B	CG101	JBUYRV010000003.1:24367-26092	1725	1	~	~
Aliivibrio fischeri_B	cmult.1.1	WOBZ01000003.1:272006-279037	7031	7	~	~
Aliivibrio fischeri_B	cmult.1.1	WOBZ01000099.1:802-3058	2256	4	~	~
Aliivibrio fischeri_B	cmult.1.1	WOBZ01000079.1:88-2065	1977	2	~	~
Aliivibrio fischeri_B	cmult.1.1	WOBZ01000066.1:27-1584	1557	1	~	~
Aliivibrio fischeri_B	cmult.1.2	WOBY01000014.1:650344-657375	7031	6	~	~
Aliivibrio fischeri_B	cmult.1.2	WOBY01000039.1:33-2334	2301	5	~	~
Aliivibrio fischeri_B	cmult.1.2	WOBY01000013.1:171-1750	1579	1	~	~
Aliivibrio fischeri_B	EM30	JBUYRT010000002.1:481919-486823	4904	3	~	~
Aliivibrio fischeri_B	EM30	JBUYRT010000002.1:476744-478237	1493	2	~	~
Aliivibrio fischeri_B	emors.1.1	WOBX01000033.1:722-6587	5865	7	~	~
Aliivibrio fischeri_B	emors.1.1	WOBX01000038.1:866-2929	2063	3	~	~
Aliivibrio fischeri_B	emors.10.1	WOBV01000047.1:1128-7293	6165	9	~	~
Aliivibrio fischeri_B	emors.10.1	WOBV01000083.1:2695-8097	5402	5	~	~
Aliivibrio fischeri_B	emors.10.1	WOBV01000025.1:282135-287261	5126	8	~	~
Aliivibrio fischeri_B	emors.10.1	WOBV01000061.1:216-1387	1171	1	~	~
Aliivibrio fischeri_B	emors.10.1	WOBV01000065.1:101-768	667	0	~	~
Aliivibrio fischeri_B	emors.11.1	WOBT01000006.1:748-10394	9646	12	~	~
Aliivibrio fischeri_B	emors.11.2	WOBS01000009.1:2746-9261	6515	10	~	~
Aliivibrio fischeri_B	emors.11.2	WOBS01000003.1:491339-496089	4750	5	~	~

Aliivibrio fischeri_B	emors.2.1	WOBR01000011.1:159456-169493	10037	11	~	~
Aliivibrio fischeri_B	emors.2.1	WOBR01000010.1:815-6504	5689	5	~	~
Aliivibrio fischeri_B	emors.2.1	WOBR01000029.1:1755-4764	3009	4	~	~
Aliivibrio fischeri_B	emors.2.1	WOBR01000011.1:173791-175958	2167	2	~	~
Aliivibrio fischeri_B	emors.8.7	WOBD01000002.1:4194-7413	3219	3	~	~
Aliivibrio fischeri_B	emors.8.7	WOBD01000003.1:58-744	686	0	~	~
Aliivibrio fischeri_B	emors.8.7	WOBD01000041.1:2192-3963	1771	3	~	~
Aliivibrio fischeri_B	emors.3.1	WOBP01000011.1:544-1596	1052	1	~	~
Aliivibrio fischeri_B	emors.3.1	WOBP01000029.1:13-3322	3309	6	~	~
Aliivibrio fischeri_B	emors.3.1	WOBP01000038.1:697-1748	1051	1	~	~
Aliivibrio fischeri_B	emors.4.1	WOBN01000102.1:17-1766	1749	4	~	~
Aliivibrio fischeri_B	emors.4.1	WOBN01000007.1:11-1638	1627	1	~	~
Aliivibrio fischeri_B	emors.4.2	WOBM01000007.1:356695-360624	3929	5	~	~
Aliivibrio fischeri_B	emors.4.2	WOBM01000046.1:1-1416	1415	2	~	~
Aliivibrio fischeri_B	emors.7.1	WOBG01000041.1:3-5612	5609	9	~	~
Aliivibrio fischeri_B	emors.7.1	WOBG01000037.1:1338-5756	4418	7	~	~
Aliivibrio fischeri_B	emors.7.1	WOBG01000054.1:133-2132	1999	3	~	~
Aliivibrio fischeri_B	emors.7.2	WOBF01000041.1:1-4693	4692	5	~	~
Aliivibrio fischeri_B	emors.7.2	WOBF01000062.1:1224-3247	2023	4	~	~
Aliivibrio fischeri_B	emors.7.2	WOBF01000032.1:2842-4314	1472	1	~	~
Aliivibrio fischeri_B	emors.7.2	WOBF01000046.1:92-1390	1298	1	~	~
Aliivibrio fischeri_B	emors.8.2	WOBE01000092.1:268-3240	2972	4	~	~
Aliivibrio fischeri_B	emors.9.2	WOBB01000022.1:650-7349	6699	7	~	~
Aliivibrio fischeri_B	emors.9.2	WOBB01000025.1:11343-13067	1724	2	~	~
Aliivibrio fischeri_B	ES401	SRJG01000002.1:808081-810568	2487	3	~	~
Aliivibrio fischeri_B	ESF436_3	JAJVEE010000011.1:22067-27301	5234	5	~	~
Aliivibrio fischeri_B	ESF436_3	JAJVEE010000008.1:3238-5124	1886	2	~	~
Aliivibrio fischeri_B	ESF436_3	JAJVEE010000011.1:430-2312	1882	3	~	~
Aliivibrio fischeri_B	ESF436_4	JAJVEF010000005.1:205689-210923	5234	5	~	~
Aliivibrio fischeri_B	ESF436_4	JAJVEF010000005.1:172862-174748	1886	2	~	~
Aliivibrio fischeri_B	ESF436_4	JAJVEF010000005.1:184052-185934	1882	3	~	~
Aliivibrio fischeri_B	ESF436_6	JAJVEH010000010.1:108485-113719	5234	5	~	~
Aliivibrio fischeri_B	ESF436_6	JAJVEH010000007.1:3238-5124	1886	2	~	~
Aliivibrio fischeri_B	ESF436_6	JAJVEH010000010.1:133474-135356	1882	3	~	~
Aliivibrio fischeri_B	ESM429_1	JAJVEB010000001.1:689606-692666	3060	4	~	~
Aliivibrio fischeri_B	ESM429_1	JAJVEB010000027.1:985-2394	1409	2	~	~

Aliivibrio fischeri_B	ESM429_3	JAJVEC010000001.1:689436-692496	3060	4	~	~
Aliivibrio fischeri_B	ESM429_4	JAJVED010000002.1:335771-340132	4361	8	~	~
Aliivibrio fischeri_B	etasm.2.1	WOAZ01000007.1:180864-186117	5253	7	~	~
Aliivibrio fischeri_B	FQ-A001	SJSX01000004.1:188896-194557	5661	7	~	~
Aliivibrio fischeri_B	FQ-A001	SJSX01000004.1:172023-176589	4566	5	~	~
Aliivibrio fischeri_B	FQ-A001	SJSX01000002.1:1183341-1186927	3586	3	~	~
Aliivibrio fischeri_B	FQ-A001	SJSX01000004.1:200314-202452	2138	3	~	~
Aliivibrio fischeri_B	H905	NZ_JBCDWF010000002.1:698871-705625	6754	10	~	~
Aliivibrio fischeri_B	MB11B1	JBQAOX010000004.1:66067-71789	5722	7	~	~
Aliivibrio fischeri_B	MB11B1	JBQAOX010000004.1:56963-58748	1785	2	~	~
Aliivibrio fischeri_B	MB11B1	JBQAOX010000004.1:88892-90483	1591	4	~	~
Aliivibrio fischeri_B	MB14A5	JBQAPA010000002.1:329493-331707	2214	3	~	~
Aliivibrio fischeri_B	MB14A5	JBQAPA010000002.1:323177-325239	2062	3	~	~
Aliivibrio fischeri_B	MJ12	JBUYRR010000001.1:1043008-1045314	2306	3	~	~
Aliivibrio fischeri_B	MJ12	JBUYRR010000001.1:1059170-1061237	2067	2	~	~
Aliivibrio fischeri_B	MJ12	JBUYRR010000001.1:1054358-1056015	1657	2	~	~
Aliivibrio fischeri_B	mjapo.8.1	JBUYRQ010000002.1:1255950-1258365	2415	2	~	~
Aliivibrio fischeri_B	mjapo.8.1	JBUYRQ010000002.1:1247226-1249494	2268	1	~	~
Aliivibrio fischeri_B	NBRC 101058	BJTZ01000002.1:223821-225883	2062	2	~	~
Aliivibrio fischeri_B	NBRC 101058	BJTZ01000093.1:154-1903	1749	4	~	~
Aliivibrio fischeri_B	SA1	JBUYRP010000002.1:159993-161816	1823	2	~	~
Aliivibrio fischeri_B	SA1	JBUYRP010000002.1:148246-149345	1099	1	~	~
Aliivibrio fischeri_B	SA1	JBUYRP010000002.1:126686-127637	951	1	~	~
Aliivibrio logei	1S159	MAJU01000008.1:247816-253468	5652	4	~	~
Aliivibrio logei	1S159	MAJU01000027.1:111-4518	4407	8	~	~
Aliivibrio logei	1S159	MAJU01000017.1:1165-5505	4340	9	~	~
Aliivibrio logei	1S159	MAJU01000069.1:479-1644	1165	2	~	~
Aliivibrio logei	ATCC 29985	JBREXA010000001.1:435306-440208	4902	5	~	~
Aliivibrio logei	ATCC 29985	JBREXA010000001.1:564915-568649	3734	6	~	~
Aliivibrio logei	ATCC 29985	JBREXA010000001.1:529328-532007	2679	2	~	~
Aliivibrio logei	ATCC 29985	JBREXA010000001.1:535356-537979	2623	3	~	~
Aliivibrio logei	ATCC 29985	JBREXA010000001.1:466284-468739	2455	2	~	~
Aliivibrio logei	ATCC 35077	ASAH01000046.1:1632-4399	2767	2	~	~
Aliivibrio logei	ATCC 35077	ASAH01000045.1:311-2720	2409	4	~	~
Aliivibrio logei	SR45-2	JACJDQ010000096.1:225-3194	2969	5	~	~
Aliivibrio logei	SR45-2	JACJDQ010000097.1:1874-3526	1652	2	~	~

Aliivibrio logei	SR45-2	JACJDQ010000147.1:128-1737	1609	2	~	~
Aliivibrio logei	SR45-2	JACJDQ010000132.1:229-1625	1396	2	~	~
Aliivibrio logei	SR45-2	JACJDQ010000168.1:3-997	994	1	~	~
Aliivibrio logei	SR45-2	JACJDQ010000171.1:1-795	794	1	~	~
Aliivibrio salmonicida	12-BacKlon1	JAKZZR010000169.1:4481-7043	2562	5	~	~
Aliivibrio salmonicida	13-BacKlon4	JAKZZQ010000170.1:2-2564	2562	5	~	~
Aliivibrio salmonicida	14-Bac1099	JAKZZP010000152.1:2-4016	4014	6	~	~
Aliivibrio salmonicida	14-Bac1099	JAKZZP010000106.1:164-2202	2038	1	~	~
Aliivibrio salmonicida	15-Vs224	JAKZZO010000170.1:2-2564	2562	5	~	~
Aliivibrio salmonicida	JAMM1233	JAKJUB010000099.1:1-5370	5369	6	~	~
Aliivibrio salmonicida	JAMM1233	JAKJUB010000126.1:495-4295	3800	5	~	~
Aliivibrio salmonicida	JAMM1233	JAKJUB010000100.1:2-1853	1851	3	~	~
Aliivibrio salmonicida	LFI1238	FM178379.1:1747383-1751049	3666	7	~	~
Aliivibrio salmonicida	SP1	JBREXB010000001.1:146363-152419	6056	10	~	~
Aliivibrio salmonicida	SP1	JBREXB010000001.1:1317983-1323200	5217	6	~	~
Aliivibrio salmonicida	SP1	JBREXB010000009.1:6717-10852	4135	5	~	~
Aliivibrio salmonicida	SP1	JBREXB010000025.1:20772-24906	4134	6	~	~
Aliivibrio salmonicida	SP1	JBREXB010000001.1:134181-137972	3791	2	~	~
Aliivibrio salmonicida	SP1	JBREXB010000001.1:89971-92631	2660	5	~	~
Aliivibrio salmonicida	Vs224	CP034241.1:610514-613143	2629	3	~	~
Aliivibrio salmonicida	Vs224	CP034240.1:1671145-1673359	2214	5	~	~
Aliivibrio sifiae	2025O3Ga	JBXBSD010000007.1:1357045-1358032	987	3	~	~
Aliivibrio sifiae	CL1	LNRI01000018.1:5097-9465	4368	5	~	~
Aliivibrio sifiae	CL1	LNRI01000017.1:11541-14605	3064	4	~	~
Aliivibrio sifiae	CL1	LNRI01000017.1:930-2474	1544	2	~	~
Aliivibrio sifiae	CL3	LNRJ01000021.1:1-4227	4226	5	~	~
Aliivibrio sifiae	CL3	LNRJ01000016.1:3826-8010	4184	5	~	~
Aliivibrio sifiae	CL3	LNRJ01000023.1:587-2987	2400	2	~	~
Aliivibrio sifiae	CL3	LNRJ01000015.1:3307-5606	2299	3	~	~
Aliivibrio sifiae	CL3	LNRJ01000020.1:4267-5983	1716	2	~	~
Aliivibrio sifiae	CL4	LNRK01000019.1:3383-6608	3225	2	~	~
Aliivibrio sifiae	CL5	LNRL01000018.1:3383-6608	3225	2	~	~
Aliivibrio sifiae	CL6	LNRM01000020.1:652-5650	4998	6	~	~
Aliivibrio sifiae	CL6	LNRM01000018.1:240-4319	4079	5	~	~
Aliivibrio sifiae	CL6	LNRM01000018.1:23904-26686	2782	4	~	~
Aliivibrio sifiae	CL6	LNRM01000026.1:4358-6118	1760	3	~	~

Aliivibrio sifiae	CL7	LNRN01000004.1:364966-368673	3707	3	~	~
Aliivibrio sifiae	CL7	LNRN01000013.1:6509-10108	3599	3	~	~
Aliivibrio sifiae	CL7	LNRN01000021.1:1166-3363	2197	3	~	~
Aliivibrio sifiae	CL7	LNRN01000035.1:6-1220	1214	1	~	~
Aliivibrio sifiae	CL7	LNRN01000008.1:8281-9243	962	3	~	~
Aliivibrio sifiae	JAMM1683	JAKJTF010000008.1:2-3454	3452	4	~	~
Aliivibrio sifiae	JAMM1683	JAKJTF010000061.1:471-2461	1990	2	~	~
Aliivibrio sifiae	JAMM1683	JAKJTF010000079.1:486-1803	1317	2	~	~
Aliivibrio sifiae	JAMM1683	JAKJTF010000065.1:161-1425	1264	1	~	~
Aliivibrio sifiae	NBRC 100501	MSCP01000002.1:940823-948721	7898	11	~	~
Aliivibrio sifiae	NBRC 100501	MSCP01000002.1:867706-873278	5572	1	~	~
Aliivibrio sifiae	NBRC 100501	MSCP01000002.1:974281-978044	3763	5	~	~
Aliivibrio sifiae	NBRC 100501	MSCP01000002.1:886669-888601	1932	4	~	~
Aliivibrio sifiae	NBRC 100501	MSCP01000002.1:934102-935877	1775	3	~	~
Aliivibrio sifiae_A	ATCC 33715	MSCO01000001.1:1453853-1456352	2499	1	~	~
Aliivibrio sifiae_A	ATCC 33715	MSCO01000002.1:593375-595190	1815	4	~	~
Aliivibrio sifiae_A	ATCC 33715	MSCO01000002.1:542935-544720	1785	4	~	~
Aliivibrio sifiae_A	ATCC 33715	MSCO01000002.1:584128-585848	1720	3	~	~
Aliivibrio sp.	S2MY1	NZ_JARACN010000047.1:343-7483	7140	9	~	~
Aliivibrio sp.	S2MY1	NZ_JARACN010000058:162-3766	3604	5	~	~
Aliivibrio sp.	S2MY1	NZ_JARACN010000055:1236-3417	2181	4	~	~
Aliivibrio sp.	S2MY1	NZ_JARACN010000052.1:2199-4310	2111	1	~	~
Aliivibrio sp.	S2MY1	NZ_JARACN010000059:24-1643	1619	3	~	~
Aliivibrio sp.	S2MY1	NZ_JARACN010000072:113-1468	1355	1	~	~
Aliivibrio sp.	S2MY1	NZ_JARACN010000084:1-1116	1115	1	~	~
Aliivibrio sp.	S3MY1	NZ_JARACO010000044.1:558-7698	7140	9	~	~
Aliivibrio sp.	S3MY1	NZ_JARACO010000056.1:2-3606	3604	5	~	~
Aliivibrio sp.	S3MY1	NZ_JARACO010000054.1:427-2608	2181	4	~	~
Aliivibrio sp.	S3MY1	NZ_JARACO010000057.1:2062-3681	1619	3	~	~
Aliivibrio sp.	S3MY1	NZ_JARACO010000070.1:113-1468	1355	1	~	~
Aliivibrio sp.	S3MY1	NZ_JARACO010000080.1:45-1160	1115	1	~	~
Aliivibrio sp.	S3MY1	NZ_JARACO010000083.1:3-967	964	1	~	~
Aliivibrio sp.	S4MY1	NZ_JARACP010000034.1:573-6614	6041	7	~	~
Aliivibrio sp.	S4MY1	NZ_JARACP010000036.1:459-3527	3068	3	~	~
Aliivibrio sp.	S4MY1	NZ_JARACP010000027.1:530-2509	1979	1	~	~
Aliivibrio sp.	S4MY1	NZ_JARACP010000031.1:4303-6149	1846	3	~	~

Aliivibrio sp.	S4MY2	NZ_JARACU010000035.1:3364-9405	6041	7	~	~
Aliivibrio sp.	S4MY2	NZ_JARACU010000037.1:3973-7041	3068	3	~	~
Aliivibrio sp.	S4MY2	NZ_JARACU010000028.1:530-2509	1979	1	~	~
Aliivibrio sp.	S4MY2	NZ_JARACU010000032.1:4303-6149	1846	3	~	~
Aliivibrio sp.	S4MY3	NZ_JARACV010000038.1:3364-9405	6041	7	~	~
Aliivibrio sp.	S4MY3	NZ_JARACV010000040.1:459-3527	3068	3	~	~
Aliivibrio sp.	S4MY3	NZ_JARACV010000030.1:530-2509	1979	1	~	~
Aliivibrio sp.	S4MY3	NZ_JARACV010000035.1:4303-6149	1846	3	~	~
Aliivibrio sp.	S4MY4	NZ_JARACW010000035.1:3364-9405	6041	7	~	~
Aliivibrio sp.	S4MY4	NZ_JARACW010000037.1:3973-7041	3068	3	~	~
Aliivibrio sp.	S4MY4	NZ_JARACW010000028.1:530-2509	1979	1	~	~
Aliivibrio sp.	S4MY4	NZ_JARACW010000032.1:4303-6149	1846	3	~	~
Aliivibrio sp.	S4TY1	NZ_JARACQ010000036.1:573-6614	6041	7	~	~
Aliivibrio sp.	S4TY1	NZ_JARACQ010000039.1:3973-7041	3068	3	~	~
Aliivibrio sp.	S4TY1	NZ_JARACQ010000028.1:543-2522	1979	1	~	~
Aliivibrio sp.	S4TY1	NZ_JARACQ010000033.1:9226-11072	1846	3	~	~
Aliivibrio sp.	S4TY2	NZ_JARACX010000033.1:3364-9405	6041	7	~	~
Aliivibrio sp.	S4TY2	NZ_JARACX010000036.1:459-3527	3068	3	~	~
Aliivibrio sp.	S4TY2	NZ_JARACX010000026.1:543-2522	1979	1	~	~
Aliivibrio sp.	S4TY2	NZ_JARACX010000030.1:9226-11072	1846	3	~	~
Aliivibrio sp.	Ust_30	JAAGZR010000729.1:1-1341	1340	2	~	~
Aliivibrio sp.	Ust_30	JAAGZR010000075.1:198-1390	1192	2	~	~
Aliivibrio wodanis	BL6	JBXXRA010000001.1:1126239-1130652	4413	6	~	~
Aliivibrio wodanis	BL6	JBXXRA010000001.1:1134758-1138483	3725	6	~	~
Aliivibrio wodanis	BL6	JBXXRA010000002.1:1375258-1378712	3454	3	~	~
Aliivibrio wodanis	BL6	JBXXRA010000001.1:1143561-1146351	2790	3	~	~
Aliivibrio wodanis	BL6	JBXXRA010000001.1:1150475-1153030	2555	4	~	~
Aliivibrio wodanis	NVI 06/09/139	LN554847.1:786498-790360	3862	5	~	~
Aliivibrio wodanis	NVI 06/09/139	LN554847.1:813692-816791	3099	4	~	~
Aliivibrio wodanis	NVI 06/09/139	LN554847.1:623382-626275	2893	5	~	~
Aliivibrio wodanis	NVI 06/09/139	LN554848.1:51662-53825	2163	3	~	~
Aliivibrio wodanis	NVI 06/09/139	LN554847.1:820204-822343	2139	2	~	~
Aliivibrio wodanis	NVI 06/09/139	LN554847.1:840839-842727	1888	2	~	~
Aliivibrio wodanis	SP11	JBREXC010000009.1:54963-61045	6082	8	~	~
Aliivibrio wodanis	SP11	JBREXC010000009.1:116818-119072	2254	2	~	~
Aliivibrio_A kagoshimensis	JAMM0721	JAKJSQ010000105.1:6144-8610	2466	3	~	~

Aliivibrio sifiae	CL7	LNRN01000014.1:1514-5110	3596	5	~	2-thiouracil desulfurase
Aliivibrio sp.	S4MY2	NZ_JARACU010000036.1:542-7373	6831	5	~	2-thiouracil desulfurase, YiiX/YebB-like N1pC/P60 family cysteine hydrolase
Aliivibrio "thorii"	SA5	JBXXRC010000002.1:1178313-1179713	1400	2	~	AAA family ATPase
Aliivibrio "thorii"	SA6	JBXXRD010000002.1:1178303-1179703	1400	2	~	AAA family ATPase
Aliivibrio fischeri	emors.6.1	WOBI01000005.1:428-9064	8636	9	~	AAA family ATPase
Aliivibrio fischeri	emors.6.2	WOBH01000026.1:1349-12832	11483	12	~	AAA family ATPase
Aliivibrio fischeri_B	emors.3.2	WOBO01000043.1:3-4429	4426	5	~	AAA family ATPase
Aliivibrio fischeri_B	emors.7.2	WOBF01000033.1:445-1708	1263	2	~	AAA family ATPase
Aliivibrio fischeri_B	emors.9.1	WOBC01000010.1:648-2617	1969	3	~	AAA family ATPase
Aliivibrio sp.	S2MY1	NZ_JARACN010000049.1:2284-6506	4222	3	~	AAA family ATPase
Aliivibrio "thorii"	MdR7	JBXXRJ010000001.1:1137828-1145472	7644	9	~	AAA family ATPase (2)
Aliivibrio fischeri_B	ESM429_4	JAJVED010000031.1:3-14228	14225	21	~	AAA family ATPase, addiction module rich
Aliivibrio fischeri_B	emors.1.1	WOBX01000025.1:1084-12002	10918	12	~	AAA family ATPase, amino acid racemase
Aliivibrio fischeri_B	emors.3.1	WOBP01000012.1:603-11807	11204	14	~	AAA family ATPase, amino acid racemase
Aliivibrio fischeri_B	ESF436_5	JAJVEG010000023.1:1487-4394	2907	4	~	AAA family ATPase, HNH endonuclease
Aliivibrio sifiae	CL6	LNRM01000019.1:231-10773	10542	12	~	Abi family protein, Rho-binding antiterminator
Aliivibrio "friggae"	SR6	JBXXRF010000002.1:53771-125923	72152	74	~	Abi, PhzF family protein (2 copies), mobilome CxxCx(11)CxxC protein, alpha/beta hydrolase, restriction endonuclease family protein (multiple), arginase family protein, cdd, anti-phage deoxyguanosine triphosphatase, P-loop nucleotide/nucleoside kinase family protein, Rho-binding antiterminator, addiction module rich
Aliivibrio "friggae"	ATCC 15382	CM170639.1:1240882-1272063	31181	39	~	AbiC, type IV RM, bleomycin binding family protein
Aliivibrio fischeri	5LC	NZ_CVOI01000002.1:662342-666377	4035	4	~	AbiTii domain-containing protein, P-loop NTPase fold protein
Aliivibrio wodanis	BL6	JBXXRA010000001.1:1165121-1166619	1498	2	~	AbiU2 domain-containing protein
Aliivibrio sifiae	CL4	LNRK01000019.1:9680-15736	6056	7	~	AbiU2 domain-containing protein, flavodoxin WrbA family protein
Aliivibrio sifiae	CL5	LNRL01000018.1:9680-15736	6056	7	~	AbiU2 domain-containing protein, flavodoxin WrbA family protein

Aliivibrio "thorii"	MdR7	JBXXRJ010000002.1:1553253-1556030	2777	5	~	abortive infection system antitoxin AbiGi family protein
Aliivibrio fischeri	lpeal.1.1	WOAY01000047.1:148-3446	3298	5	~	adaB
Aliivibrio fischeri_B	emors.4.1	WOBN01000006.1:2044-11896	9852	17	~	adaB
Aliivibrio finisterrensis	LMG 23869	WBVP01000040.1:1040-4623	3583	5	~	adaB, HNH endonuclease
Aliivibrio fischeri	ES213	NZ_LTYU01000061.1:2173-9881	7708	17	~	addiction module rich
Aliivibrio fischeri	MB11B1	NZ_LTYY01000075.1:962-10945	9983	16	~	addiction module rich
Aliivibrio fischeri	MB13B2	NZ_LTZB01000060.1:12-9995	9983	16	~	addiction module rich
Aliivibrio fischeri	MB13B3	NZ_LTZC01000030.1:1614-11981	10367	18	~	addiction module rich
Aliivibrio sifiae	JAMM1683	JAKJTF010000063.1:105-3171	3066	3	~	alkD
Aliivibrio "friggae"	SR10	JBXXRI010000001.1:1038156-1042136	3980	5	~	alkD, gstA
Aliivibrio "friggae"	SR8	JBXXRG010000001.1:1037066-1041046	3980	5	~	alkD, gstA
Aliivibrio logei	1S159	MAJU01000020.1:2-5263	5261	6	~	alpha/beta hydrolase
Aliivibrio logei	ATCC 29985	JBREXA010000002.1:867305-869304	1999	3	~	alpha/beta hydrolase
Aliivibrio fischeri	etasm.1.1	WOBA01000021.1:5893-16915	11022	10	~	amino acid racemase
Aliivibrio fischeri_B	emors.3.2	WOBO01000011.1:518-5704	5186	7	~	amino acid racemase, SDR family oxidoreductase
Aliivibrio "friggae"	EL58	NZ_OMPC01000003.1:742-6448	5706	4	~	antiviral reverse transcriptase Drt2
Aliivibrio salmonicida	14-Bac1099	JAKZZP010000106.1:8659-11137	2478	3	~	antiviral reverse transcriptase Drt4
Aliivibrio salmonicida	LFI1238	FM178380.1:605267-613092	7825	11	~	antiviral reverse transcriptase Drt4
Aliivibrio salmonicida	Vs224	CP034241.1:605318-607796	2478	3	~	antiviral reverse transcriptase Drt4
Aliivibrio sp.	S4MY1	NZ_JARACP010000031.1:12428-15372	2944	2	~	antiviral reverse transcriptase Drt4
Aliivibrio sp.	S4MY2	NZ_JARACU010000032.1:12428-15372	2944	2	~	antiviral reverse transcriptase Drt4
Aliivibrio sp.	S4MY3	NZ_JARACV010000035.1:12428-15372	2944	2	~	antiviral reverse transcriptase Drt4
Aliivibrio sp.	S4MY4	NZ_JARACW010000032.1:12428-15372	2944	2	~	antiviral reverse transcriptase Drt4
Aliivibrio sp.	S4TY1	NZ_JARACQ010000033.1:3-2947	2944	2	~	antiviral reverse transcriptase Drt4
Aliivibrio sp.	S4TY2	NZ_JARACX010000030.1:3-2947	2944	2	~	antiviral reverse transcriptase Drt4
Aliivibrio salmonicida	12-BacKlon1	JAKZZR010000104.1:164-11137	10973	14	~	antiviral reverse transcriptase Drt4, protein-threonine AMPylation (FIDO) domain protein, RES domain protein
Aliivibrio salmonicida	13-BacKlon4	JAKZZQ010000105.1:1-13235	13234	16	~	antiviral reverse transcriptase Drt4, protein-threonine AMPylation (FIDO) domain protein, RES domain protein
Aliivibrio salmonicida	15-Vs224	JAKZZO010000104.1:164-11137	10973	14	~	antiviral reverse transcriptase Drt4, protein-threonine AMPylation (FIDO) domain protein, RES domain protein
Aliivibrio fischeri	SR5	AHIH01000015.1:5607-23817	18210	20	~	aspartate racemase, gloA
Aliivibrio sp.	S4MY1	NZ_JARACP010000030.1:14515-18236	3721	2	~	bacterial retron reverse transcriptase
Aliivibrio sp.	S4MY2	NZ_JARACU010000031.1:14515-18236	3721	2	~	bacterial retron reverse transcriptase

Aliivibrio sp.	S4MY3	NZ_JARACV010000034.1:1-3722	3721	2	~	bacterial retron reverse transcriptase
Aliivibrio sp.	S4MY4	NZ_JARACW010000031.1:14515-18236	3721	2	~	bacterial retron reverse transcriptase
Aliivibrio sp.	S4TY1	NZ_JARACQ010000032.1:14515-18236	3721	2	~	bacterial retron reverse transcriptase
Aliivibrio sp.	S4TY2	NZ_JARACX010000029.1:14515-18236	3721	2	~	bacterial retron reverse transcriptase
Aliivibrio fischeri	10N.261.55.A3	JBFRMZ010000114.1:249-2315	2066	4	~	Barstar RNAse (barnase) inhibitor family protein
Aliivibrio sifiae	CL6	LNRM01000024.1:5361-9819	4458	4	~	beta/gamma crystallin domain containing protein
Aliivibrio sp.	S4MY1	NZ_JARACP010000030.1:1500-11291	9791	12	~	Cap15 family cyclic dinucleotide receptor domain-containing protein, RM, nucleotidyltransferase family protein
Aliivibrio sp.	S4MY2	NZ_JARACU010000031.1:1500-11291	9791	12	~	Cap15 family cyclic dinucleotide receptor domain-containing protein, RM, nucleotidyltransferase family protein
Aliivibrio sp.	S4MY3	NZ_JARACV010000034.1:6946-16737	9791	12	~	Cap15 family cyclic dinucleotide receptor domain-containing protein, RM, nucleotidyltransferase family protein
Aliivibrio sp.	S4MY4	NZ_JARACW010000031.1:1500-11291	9791	12	~	Cap15 family cyclic dinucleotide receptor domain-containing protein, RM, nucleotidyltransferase family protein
Aliivibrio sp.	S4TY1	NZ_JARACQ010000032.1:1500-11291	9791	12	~	Cap15 family cyclic dinucleotide receptor domain-containing protein, RM, nucleotidyltransferase family protein
Aliivibrio sp.	S4TY2	NZ_JARACX010000029.1:1500-11291	9791	12	~	Cap15 family cyclic dinucleotide receptor domain-containing protein, RM, nucleotidyltransferase family protein
Aliivibrio "thorii"	SA5	JBXXRC010000002.1:1133852-1137273	3421	7	~	caspase family protein
Aliivibrio "thorii"	SA6	JBXXRD010000002.1:1133853-1137274	3421	7	~	caspase family protein
Aliivibrio fischeri	5F146	MAJR01000003.1:82757-86536	3779	4	~	caspase family protein
Aliivibrio fischeri_B	emors.8.7	WOBD01000007.1:275123-278902	3779	4	~	caspase family protein
Aliivibrio fischeri_B	ES401	SRJG01000005.1:212208-216443	4235	7	~	caspase family protein
Aliivibrio sifiae	CL1	LNRI01000015.1:17241-25588	8347	10	~	caspase family protein, RIP homotypic interaction motif-containing protein, nucleotidyltransferase family protein
Aliivibrio fischeri	MB15A5	NZ_LTZE01000026.1:14902-30103	15201	17	~	caspase family proteins, class I SAM-dependent methyltransferase

Aliivibrio finisterrensis_A	A2	NZ_SEZS0100041.1:6350-8059	1709	2	~	class I SAM-dependent methyltransferase
Aliivibrio finisterrensis_A	A33	NZ_SEZQ0100037.1:6350-8059	1709	2	~	class I SAM-dependent methyltransferase
Aliivibrio finisterrensis_A	A4	NZ_SEZR0100037.1:6350-8059	1709	2	~	class I SAM-dependent methyltransferase
Aliivibrio fischeri	KB4B5	NZ_LTZD01000010.1:2808-5858	3050	4	~	class I SAM-dependent methyltransferase
Aliivibrio fischeri	MB14A3	NZ_LTYX01000020.1:1043-5757	4714	6	~	class I SAM-dependent methyltransferase
Aliivibrio fischeri_B	ESF436_3	JAJVEE010000011.1:30780-33409	2629	3	~	class I SAM-dependent methyltransferase
Aliivibrio fischeri_B	ESF436_4	JAJVEF010000005.1:214402-217031	2629	3	~	class I SAM-dependent methyltransferase
Aliivibrio fischeri_B	ESF436_6	JAJVEH010000010.1:102377-105006	2629	3	~	class I SAM-dependent methyltransferase
Aliivibrio sifiae	CL4	LNRK01000020.1:8693-17242	8549	11	~	class I SAM-dependent methyltransferase
Aliivibrio sifiae	CL5	LNRL01000019.1:224-8773	8549	11	~	class I SAM-dependent methyltransferase
Aliivibrio sifiae	JAMM1683	JAKJTF010000004.1:1-2062	2061	4	~	class I SAM-dependent methyltransferase
Aliivibrio "friggae"	EL58	NZ_OMPC01000003.1:10678-15232	4554	5	~	class IV adenylate cyclase
Aliivibrio fischeri_B	ESM429_1	JAJVEB010000020.1:4094-9635	5541	8	~	class IV adenylate cyclase, gstA
Aliivibrio fischeri_B	ESM429_3	JAJVEC010000020.1:9017-14558	5541	8	~	class IV adenylate cyclase, gstA
Aliivibrio sifiae	CL4	LNRK01000020.1:585-5854	5269	3	~	class IV adenylate cyclase, mod
Aliivibrio sifiae	CL5	LNRL01000023.1:585-5854	5269	3	~	class IV adenylate cyclase, mod
Aliivibrio finisterrensis_A	A21	NZ_SEZN01000069.1:587-4341	3754	3	~	cold shock/excalibur domain-containing protein
Aliivibrio finisterrensis_A	S2TY2	JARACS010000025.1:653-2531	1878	2	~	cold shock/excalibur domain-containing protein
Aliivibrio finisterrensis_A	S3TY1	JARACT010000025.1:2496-4374	1878	2	~	cold shock/excalibur domain-containing protein
Aliivibrio fischeri	KB1A98	NZ_LTYV01000044.1:1701-6480	4779	7	~	cold shock/excalibur domain-containing protein
Aliivibrio fischeri_B	emors.4.2	WOBM01000016.1:3-2268	2265	4	~	cold shock/excalibur domain-containing protein
Aliivibrio fischeri_B	H905	NZ_JBCDWF010000002.1:718008-722593	4585	6	~	cold shock/excalibur domain-containing protein
Aliivibrio sifiae	2025O3Ga	JBXBSD010000007.1:1319169-1323976	4807	8	~	cold shock/excalibur domain-containing protein
Aliivibrio sifiae	CL4	LNRK01000023.1:6102-12807	6705	9	~	cold shock/excalibur domain-containing protein

Aliivibrio sifiae	CL5	LNRL01000021.1:6102-12807	6705	9	~	cold shock/excalibur domain-containing protein
Aliivibrio fischeri	DSM 507	NZ_CP092713.1:1033901-1035487	1586	2	~	deoxycytidylate deaminase domain protein
Aliivibrio sifiae	NBRC 100501	MSCP01000002.1:880314-882718	2404	2	~	flavodoxin WbrA family protein
Aliivibrio fischeri	SR5	CM001401.1:683931-688894	4963	6	~	gloA
Aliivibrio fischeri	5F7	MCGJ01000080.1:2275-5619	3344	6	~	gstA
Aliivibrio fischeri	DSM 507	NZ_CP092713.1:1050465-1054540	4075	4	~	gstA
Aliivibrio fischeri	ZF73	MAJX01000058.1:23-2034	2011	2	~	gstA
Aliivibrio fischeri_B	emors.1.1	WOBX01000028.1:2252-8490	6238	7	~	gstA
Aliivibrio fischeri_B	emors.10.1	WOBV01000095.1:195-1551	1356	2	~	gstA
Aliivibrio fischeri_B	emors.3.1	WOBP01000047.1:2868-7151	4283	5	~	gstA
Aliivibrio fischeri_B	emors.3.2	WOBO01000039.1:608-4856	4248	5	~	gstA
Aliivibrio fischeri_B	emors.7.2	WOBF01000031.1:439-4314	3875	5	~	gstA
Aliivibrio sifiae	JAMM1683	JAKJTF010000071.1:2-3384	3382	3	~	gstA
Aliivibrio fischeri_A	HQB-91	NZ_JBHZSK010000027.1:75-2542	2467	2	~	gstA (2)
Aliivibrio "friggae"	SR9	NZ_CM170645.1:1038156-1055158	17002	17	~	gstA, AlkD_like family protein, phospholipase D-like domain-containing protein, nitrilase family protein, nucleotidyltransferase family protein
Aliivibrio wodanis	SP11	JBREXC010000009.1:104481-109440	4959	6	~	gstA, HAD hydrolase
Aliivibrio fischeri	ES213	NZ_LTYU01000052.1:826-5486	4660	6	~	gstA, Hcp family type VI secretion system effector
Aliivibrio finisterrensis_A	A15	NZ_SEZO01000083.1:20-738	718	1	~	HAD hydrolase
Aliivibrio finisterrensis_A	A21	NZ_SEZN01000076.1:25-1391	1366	2	~	HAD hydrolase
Aliivibrio finisterrensis_A	A26	NZ_SEZM01000075.:24-1390	1366	2	~	HAD hydrolase
Aliivibrio finisterrensis_A	A37	NZ_SEZL01000079.1:24-1390	1366	2	~	HAD hydrolase
Aliivibrio finisterrensis_A	A46	NZ_SEZK0100079.1:25-1391	1366	2	~	HAD hydrolase
Aliivibrio finisterrensis_A	A9	NZ_SEZP01000072.1:24-1390	1366	2	~	HAD hydrolase
Aliivibrio fischeri	lpeal.1.1	WOAY01000046.1:547-3459	2912	3	~	HAD hydrolase
Aliivibrio fischeri_A	lpeal.1.2	WOAX01000044.1:485-4306	3821	3	~	HAD hydrolase
Aliivibrio fischeri_B	emors.9.1	WOBC01000012.1:1058-2382	1324	2	~	HAD hydrolase
Aliivibrio logei	ATCC 29985	JBREXA010000001.1:446340-448705	2365	3	~	HAD hydrolase
Aliivibrio sifiae	CL6	LNRM01000022.1:46-1206	1160	3	~	HAD hydrolase
Aliivibrio sifiae_A	ATCC 33715	MSCO01000002.1:496711-506789	10078	10	~	HAD hydrolase
Aliivibrio sifiae_A	ATCC 33715	MSCO01000002.1:504511-506789	2278	4	~	HAD hydrolase
Aliivibrio fischeri	5LC	NZ_CVOI01000027.1:450-14823	14373	14	~	HAD hydrolase, KAP family P-loop NTPase fold protein

Aliivibrio sifiae	CL4	LNRK01000024.1:1092-7610	6518	9	~	HAD hydrolase, Rho-binding antiterminator
Aliivibrio sifiae	CL5	LNRL01000024.1:1092-7610	6518	9	~	HAD hydrolase, Rho-binding antiterminator
Aliivibrio fischeri	MB13B2	NZ_LTZB01000042.1:1082-3527	2445	5	~	Hcp family type VI secretion system effector, gstA
Aliivibrio fischeri_B	ESM429_4	JAJVED010000030.1:153-14574	14421	18	~	Hcp family type VI secretion system effector, gstA, AAA family ATPase
Aliivibrio fischeri	KB2B1	NZ_LTYW01000029.1:2495-13571	11076	14	~	Hcp family type VI secretion system effector, gstA, protein-threonine AMPylation (FIDO) domain protein
Aliivibrio sifiae	JAMM1683	JAKJTF010000005.1:1495-7153	5658	7	~	Hcp family type VI secretion system effector, lecithin retinol acyltransferase family protein
Aliivibrio logei	ATCC 35077	ASAH01000002.1:404861-412290	7429	9	~	HEPN/RES N-terminal domain containing protein
Aliivibrio logei	ATCC 35077	ASAH01000003.1:404861-412290	7429	9	~	HEPN/RES N-terminal domain containing protein
Aliivibrio finisterrensis_A	A4	NZ_SEZR0100046.1:243-3684	3441	3	~	HNH endonclease
Aliivibrio "thorii"	SA5	JBXXRC010000002.1:1205869-1208299	2430	3	~	HNH endonuclease
Aliivibrio "thorii"	SA6	JBXXRD010000002.1:1205859-1208289	2430	3	~	HNH endonuclease
Aliivibrio fischeri	etasm.1.1	WOBA01000024.1:16141-23845	7704	7	~	HNH endonuclease
Aliivibrio fischeri	MB13B3	NZ_LTZC01000024.1:1037-3878	2841	3	~	HNH endonuclease
Aliivibrio fischeri_A	HQB-91	NZ_JBHZSK010000020.1:188-7245	7057	7	~	HNH endonuclease
Aliivibrio fischeri_B	emors.8.7	WOBD01000042.1:1773-5626	3853	4	~	HNH endonuclease
Aliivibrio fischeri_B	ESM429_1	JAJVEB010000021.1:3826-17680	13854	16	~	HNH endonuclease
Aliivibrio fischeri_B	ESM429_3	JAJVEC010000021.1:927-17696	16769	19	~	HNH endonuclease
Aliivibrio fischeri_B	ESM442_5	JAJVEI010000005.1:77304-86800	9496	11	~	HNH endonuclease
Aliivibrio fischeri_B	ESM442_6	JAJVEJ010000003.1:173864-183360	9496	11	~	HNH endonuclease
Aliivibrio fischeri_B	MB11B1	JBQAOX010000004.1:96545-99386	2841	3	~	HNH endonuclease
Aliivibrio wodanis	BL6	JBXXRA010000001.1:1178162-1186325	8163	11	~	HNH endonuclease
Aliivibrio wodanis	SP11	JBREXC010000009.1:89738-92090	2352	3	~	HNH endonuclease
Aliivibrio fischeri	S10_S31	JAAUWR010000010.1:84494-88585	4091	6	~	HNH endonuclease, nucleotidyltransferase family protein
Aliivibrio logei	ATCC 29985	JBREXA010000001.1:473074-482119	9045	12	~	HNH endonuclease, nucleotidyltransferase family protein, class I SAM-dependent methyltransferase
Aliivibrio fischeri_B	emors.7.1	WOBG01000049.1:85-5386	5301	7	~	HNH endonuclease, protein-threonine AMPylation (FIDO) domain protein, hptA
Aliivibrio logei	ATCC 29985	JBREXA010000002.1:1212260-1214444	2184	4	~	integron integrase (pseudogene)

Aliivibrio fischeri_B	EM30	JBUYRT010000002.1:466521-472364	5843	4	~	KAP family P-loop NTPase fold protein
Aliivibrio sifiae	JAMM1683	JAKJTF010000060.1:1197-8956	7759	8	~	KAP family P-loop NTPase fold protein
Aliivibrio fischeri	SR5	AHIH01000011.1:194006-201774	7768	8	~	lactoylglutathione lyase, DnaB helicase C-terminal domain-containing protein,
Aliivibrio finisterrensis_A	A2	NZ_SEZS01000045.1:194-5817	5623	6	~	lecithin retinol acyltransferase family protein
Aliivibrio finisterrensis_A	A33	NZ_SEZQ0100056.1:91-1523	1432	4	~	lecithin retinol acyltransferase family protein
Aliivibrio finisterrensis_A	A4	NZ_SEZR0100060.1:91-1523	1432	4	~	lecithin retinol acyltransferase family protein
Aliivibrio logei	SR45-2	JACJDQ010000086.1:6288-8726	2438	2	~	lecithin retinol acyltransferase family protein
Aliivibrio sifiae	CL3	LNRJ01000017.1:3089-8082	4993	5	~	lecithin retinol acyltransferase family protein
Aliivibrio fischeri	MB15A5	NZ_LTZE01000033.1:184-2628	2444	3	~	MdaB family protein
Aliivibrio salmonicida	JAMM1233	JAKJUB010000114.1:2156-5462	3306	3	~	metal-dependent hydrolase, beta-lactamase superfamily II, 5'-nucleotidase
Aliivibrio "friggae"	SR10	JBXXRI010000001.1:1050852-1055158	4306	4	~	nucleotidyltransferase family protein
Aliivibrio "friggae"	SR8	JBXXRG010000001.1:1049762-1054068	4306	4	~	nucleotidyltransferase family protein
Aliivibrio finisterrensis	LMG 23869	WBVP01000041.1:8-1929	1921	1	~	nucleotidyltransferase family protein
Aliivibrio finisterrensis_A	S2TY2	JARACS010000021.1:2954-9003	6049	10	~	nucleotidyltransferase family protein
Aliivibrio finisterrensis_A	S3TY1	JARACT010000021.1:22928-28977	6049	10	~	nucleotidyltransferase family protein
Aliivibrio fischeri	5F23	MAJQ01000059.1:53-2216	2163	4	~	nucleotidyltransferase family protein
Aliivibrio fischeri	5LC	NZ_CVOI01000030.1:547-6384	5837	7	~	nucleotidyltransferase family protein
Aliivibrio fischeri	MB13B1	NZ_LTZA01000017.1:33-6579	6546	6	~	nucleotidyltransferase family protein
Aliivibrio fischeri_B	emors.4.1	WOBN01000064.1:3500-21145	17645	22	~	nucleotidyltransferase family protein
Aliivibrio fischeri_B	etasm.2.1	WOAZ01000007.1:170893-175020	4127	6	~	nucleotidyltransferase family protein
Aliivibrio logei	SR45-2	JACJDQ010000153.1:2-1288	1286	2	~	nucleotidyltransferase family protein
Aliivibrio fischeri_B	emors.7.1	WOBG01000038.1:11-10288	10277	15	~	nucleotidyltransferase family protein, class IV adenylate cyclase
Aliivibrio sifiae	CL4	LNRK01000028.1:181-2077	1896	3	~	nucleotidyltransferase family protein, class IV adenylate cyclase
Aliivibrio sifiae	CL5	LNRL01000027.1:181-2077	1896	3	~	nucleotidyltransferase family protein, class IV adenylate cyclase
Aliivibrio finisterrensis_A	A2	NZ_SEZS0100047.1:1-3786	3785	8	~	nucleotidyltransferase family protein, protein-threonine AMPylation (FIDO) domain protein

Aliivibrio finisterrensis_A	A33	NZ_SEZQ01000042.1:1-3786	3785	8	~	nucleotidyltransferase family protein, protein-threonine AMPylation (FIDO) domain protein,
Aliivibrio finisterrensis_A	A4	NZ_SEZR01000042.1:1-3786	3785	8	~	nucleotidyltransferase family protein, protein-threonine AMPylation (FIDO) domain protein,
Aliivibrio wodanis	SP11	JBREXC010000003.1:39740-45502	5762	6	~	OLD family predicted ATP-dependent endonuclease
Aliivibrio logei	ATCC 29985	JBREXA010000001.1:551955-560778	8823	10	~	OLD/YbjD family predicted ATP-dependent nuclease
Aliivibrio fischeri	MB13B1	NZ_LTZA01000013.1:526943-528665	1722	3	~	OSA/FiwA-like plasmid fertility inhibition factor
Aliivibrio fischeri	SR5	AHIH01000015.1:34597-48407	13810	18	~	outer membrane beta-barrel domain protein
Aliivibrio fischeri_A	emors.5.2	WOBJ01000034.1:4909-7089	2180	2	~	outer membrane beta-barrel domain protein
Aliivibrio fischeri_B	emors.3.2	WOBO01000044.1:49-1757	1708	2	~	outer membrane beta-barrel domain protein
Aliivibrio sp.	S2MY1	NZ_JARACN010000048:2405-4542	2137	5	~	outer membrane beta-barrel domain protein
Aliivibrio sp.	S3MY1	NZ_JARACO010000047.1:2725-4862	2137	5	~	outer membrane beta-barrel domain protein
Aliivibrio fischeri_B	emors.1.1	WOBX01000035.1:50-1758	1708	2	~	outer membrane protein beta-barrel domain protein
Aliivibrio fischeri_B	emors.3.1	WOBP01000020.1:7170-11412	4242	4	~	panB
Aliivibrio fischeri_B	ESM442_5	JAJVEI010000005.1:62879-65870	2991	6	~	Pas factor saposin fold domain containing protein
Aliivibrio fischeri_B	ESM442_6	JAJVEJ010000003.1:194794-197785	2991	6	~	Pas factor saposin fold domain containing protein
Aliivibrio fischeri_B	emors.8.7	WOBD01000039.1:560-8863	8303	11	~	peptide deformylase family protein; HAD hydrolase
Aliivibrio finisterrensis_A	A15	NZ_SEZO01000059.1:323-8624	8301	12	~	phospholipase D-like domain-containing protein
Aliivibrio finisterrensis_A	A21	NZ_SEZN01000060:2039-10340	8301	13	~	phospholipase D-like domain-containing protein
Aliivibrio finisterrensis_A	A26	NZ_SEZM01000066.1:733-.14727	3994	8	~	phospholipase D-like domain-containing protein
Aliivibrio finisterrensis_A	A37	NZ_SEZL01000061.1:323-8624	8301	12	~	phospholipase D-like domain-containing protein
Aliivibrio finisterrensis_A	A46	NZ_SEZK01000070.1:733-4727	3994	8	~	phospholipase D-like domain-containing protein
Aliivibrio finisterrensis_A	A6	JARACR010000058.1:434-8735	8301	12	~	phospholipase D-like domain-containing protein
Aliivibrio finisterrensis_A	A9	NZ_SEZP01000056.1:323-8624	8301	13	~	phospholipase D-like domain-containing protein

Aliivibrio fischeri	5F7	MCGJ01000040.1:31-9622	9591	12	~	PhzF family protein
Aliivibrio fischeri	etasm.1.1	WOBA01000023.1:2809-7820	5011	6	~	PhzF family protein
Aliivibrio sp.	S2MY1	NZ_JARACN010000071:1-1600	1599	3	~	PhzF family protein
Aliivibrio sp.	S3MY1	NZ_JARACO010000069.1:1-1600	1599	3	~	PhzF family protein
Aliivibrio salmonicida	SP1	JBREXB010000001.1:1143486-1152404	8918	10	~	potassium channel family protein
Aliivibrio sifiae	CL4	LNRK01000022.1:1325-11331	10006	11	~	pPIWI-associating nuclease domain-containing protein, abortive infection system antitoxin AbiGi family protein
Aliivibrio sifiae	CL5	LNRL01000020.1:1325-11331	10006	11	~	pPIWI-associating nuclease domain-containing protein, abortive infection system antitoxin AbiGi family protein
Aliivibrio fischeri_B	emors.4.1	WOBN01000062.1:7197-10701	3504	3	~	protein kinase catalytic domain protein
Aliivibrio "vili"	1S165	MAJT01000006.1:3363-7697	4334	6	~	protein-threonine AMPylation (FIDO) domain protein
Aliivibrio "vili"	1S175	MAJS01000017.1:2298-4152	1854	4	~	protein-threonine AMPylation (FIDO) domain protein
Aliivibrio fischeri	5F33	MAJO01000057.1:1107-2853	1746	3	~	protein-threonine AMPylation (FIDO) domain protein
Aliivibrio fischeri	DSM 507	NZ_CP092713.1:1095349-1097148	1799	4	~	protein-threonine AMPylation (FIDO) domain protein
Aliivibrio fischeri	emors.6.1	WOBI01000007.1:333-5877	5544	9	~	protein-threonine AMPylation (FIDO) domain protein
Aliivibrio fischeri	emors.6.2	WOBH01000027.1:134-8210	8076	11	~	protein-threonine AMPylation (FIDO) domain protein
Aliivibrio fischeri	etasm.1.1	WOBA01000017.1:4056-9633	5577	5	~	protein-threonine AMPylation (FIDO) domain protein
Aliivibrio fischeri	LB10LO6	JAJVEL010000028.1:471-3937	3466	6	~	protein-threonine AMPylation (FIDO) domain protein
Aliivibrio fischeri	MB11B1	NZ_LTYY01000032.1:153-7011	6858	7	~	protein-threonine AMPylation (FIDO) domain protein
Aliivibrio fischeri	MB15A4	NZ_LTYZ01000014.1:544-3417	2873	5	~	protein-threonine AMPylation (FIDO) domain protein
Aliivibrio fischeri	ZF47	MAJY01000038.1:1101-8544	7443	13	~	protein-threonine AMPylation (FIDO) domain protein
Aliivibrio fischeri_A	lpeal.1.2	WOAX01000021.1:34-1833	1799	4	~	protein-threonine AMPylation (FIDO) domain protein
Aliivibrio fischeri_B	BW9	JBTYYO010000004.1:44253-53007	8754	13	~	protein-threonine AMPylation (FIDO) domain protein
Aliivibrio fischeri_B	emors.10.3	WOBU01000065.1:769-2319	1550	4	~	protein-threonine AMPylation (FIDO) domain protein
Aliivibrio fischeri_B	emors.3.1	WOBP01000036.1:2405-10887	8482	10	~	protein-threonine AMPylation (FIDO) domain protein

Aliivibrio fischeri_B	emors.3.2	WOBO01000010.1:143-13117	12974	13	~	protein-threonine AMPylation (FIDO) domain protein
Aliivibrio fischeri_B	emors.4.2	WOBM01000016.1:9920-18617	8697	12	~	protein-threonine AMPylation (FIDO) domain protein
Aliivibrio fischeri_B	etasm.2.1	WOAZ01000007.1:188860-196922	8062	11	~	protein-threonine AMPylation (FIDO) domain protein
Aliivibrio sifiae	CL6	LNRM01000018.1:8879-17860	8981	9	~	protein-threonine AMPylation (FIDO) domain protein
Aliivibrio finisterrensis_A	S2TY2	JARACS010000022.1:9126-21948	12822	14	~	protein-threonine AMPylation (FIDO) domain protein, AAA family ATPase, nitrilase family protein, nucleotidyltransferase family protein, Abi family protein
Aliivibrio finisterrensis_A	S3TY1	JARACT010000022.1:5338-18160	12822	14	~	protein-threonine AMPylation (FIDO) domain protein, AAA family ATPase, nitrilase family protein, nucleotidyltransferase family protein, Abi family protein
Aliivibrio "friggae"	ATCC 15382	CM170639.1:1217727-1225510	7783	9	~	protein-threonine AMPylation (FIDO) domain protein, gstA
Aliivibrio fischeri	LB10LO6	JAJVEL010000024.1:763-5662	4899	7	~	protein-threonine AMPylation (FIDO) domain protein, HNH endonuclease
Aliivibrio finisterrensis	LMG 23869	WBVP01000035.1:44-11393	11349	12	~	pseudogene rich
Aliivibrio finisterrensis	LMG 23869	WBVP01000037.1:543-7055	6512	6	~	pseudogene rich
Aliivibrio fischeri_B	BW9	JBTYYO010000004.1:56828-61641	4813	3	~	pseudogene rich
Aliivibrio fischeri_B	MB11B1	JBQAOX010000004.1:75189-82273	7084	5	~	pseudogene rich
Aliivibrio fischeri_A	emors.5.2	WOBJ01000047.1:910-3021	2111	4	~	radical SAM protein
Aliivibrio "friggae"	SR10	JBXXRI010000001.1:1065370-1073028	7658	10	~	Ras-like GTPase domain-containing protein, Rho-binding antiterminator, antiviral reverse transcriptase Drt2
Aliivibrio "friggae"	SR8	JBXXRG010000001.1:1064280-1071938	7658	10	~	Ras-like GTPase domain-containing protein, Rho-binding antiterminator, antiviral reverse transcriptase Drt2
Aliivibrio "friggae"	SR9	NZ_CM170645.1:1065370-1073028	7658	9	~	Ras-like GTPase domain-containing protein, Rho-binding antiterminator, antiviral reverse transcriptase Drt2
Aliivibrio "friggae"	SR10	JBXXRI010000002.1:1504671-1509759	5088	4	~	RES domain protein
Aliivibrio "friggae"	SR8	JBXXRG010000003.1:1504629-1509717	5088	4	~	RES domain protein
Aliivibrio "friggae"	SR9	NZ_CM170644.1:1504671-1509759	5088	4	~	RES domain protein
Aliivibrio fischeri_B	emors.1.1	WOBX01000026.1:7572-13426	5854	7	~	RES domain protein
Aliivibrio fischeri_B	emors.3.2	WOBO01000009.1:2128-14755	12627	11	~	RES domain-containing protein
Aliivibrio fischeri_A	emors.5.2	WOBJ01000032.1:75-10911	10836	13	~	retron Ec67 family RNA-directed DNA polymerase/endonuclease

Aliivibrio sp.	S2MY1	NZ_JARACN010000046:415-2159	1744	3	~	Rho-binding antiterminator
Aliivibrio sp.	S3MY1	NZ_JARACO010000046.1:415-2159	1744	3	~	Rho-binding antiterminator
Aliivibrio sp.	S3MY1	NZ_JARACO010000040.1:2284-13606	11322	9	~	Rho-binding antiterminator, AAA family ATPase
Aliivibrio logei	ATCC 35077	NZ_ASAH01000024.1:1313-16258	14945	13	~	Rho-binding antiterminator, HAD hydrolase, MBL fold metallo-hydrolase
Aliivibrio finisterrensis_A	S2TY2	JARACS010000021.1:15010-18383	3373	4	~	Rho-binding antiterminator, Pas factor domain protein
Aliivibrio finisterrensis_A	S3TY1	JARACT010000021.1:13548-16921	3373	4	~	Rho-binding antiterminator, Pas factor domain protein
Aliivibrio sifiae	CL4	LNRK01000031.1:2-1991	1989	1	~	RhuM family protein
Aliivibrio sifiae	CL5	LNRL01000030.1:2-1991	1989	1	~	RhuM family protein
Aliivibrio fischeri_B	emors.10.1	WOBV01000001.1:754-8867	8113	10	~	ribB, HAD hydrolase
Aliivibrio fischeri	SR5	AHIH01000016.1:8329-15308	6979	13	~	restriction endonuclease family protein
Aliivibrio salmonicida	JAMM1233	JAKJUB010000028.1:11538-17920	6382	9	~	restriction endonuclease family protein
Aliivibrio fischeri	5F23	MAJQ01000078.1:154-3221	3067	3	~	SDR family oxidoreductase
Aliivibrio fischeri_B	emors.10.3	WOBU01000066.1:1-1435	1434	1	~	SDR family oxidoreductase
Aliivibrio fischeri_B	emors.9.1	WOBC01000024.1:12-8529	8517	12	~	SDR family oxidoreductase
Aliivibrio fischeri	5LC	NZ_CVOI01000025.1:2882-13990	11108	16	~	SDR family oxidoreductase, alkD, class IV adenylate cyclase, RidA family protein, HEPN_AbiU2 domain containing protein, HNH endonuclease
Aliivibrio logei	ATCC 29985	JBREXA010000001.1:505508-519744	14236	17	~	SDR family oxidoreductase, protein kinase domain-containing protein, class I SAM-dependent methyltransferase
Aliivibrio fischeri	ES213	NZ_LTYU01000001.1:451-4812	4361	8	~	Shedu immune nuclease family protein
Aliivibrio fischeri	KB2B1	NZ_LTYW01000023.1:335850-340211	4361	8	~	Shedu immune nuclease family protein
Aliivibrio fischeri	MB11B1	NZ_LTYY01000045.1:450-4805	4355	8	~	Shedu immune nuclease family protein
Aliivibrio fischeri	MB13B2	NZ_LTZB01000047.1:450-4034	3584	6	~	Shedu immune nuclease family protein
Aliivibrio fischeri	MB13B3	NZ_LTZC01000001.1:450-4811	4361	8	~	Shedu immune nuclease family protein
Aliivibrio finisterrensis_A	A2	NZ_SEZS0100040.1:102-3422	3320	3	~	SIR2 family protein
Aliivibrio finisterrensis_A	A33	NZ_SEZQ0100036.1:102-3422	3320	3	~	SIR2 family protein
Aliivibrio finisterrensis_A	A4	NZ_SEZR0100036.1:102-3422	3320	3	~	SIR2 family protein

Aliivibrio finisterrensis_A	A15	NZ_SEZO01000074:679-3718	3039	2	~	S-type pyocin T6SS effector
Aliivibrio finisterrensis_A	A21	NZ_SEZN01000072:679-3718	3039	2	~	S-type pyocin T6SS effector
Aliivibrio finisterrensis_A	A26	NZ_SEZM01000070:679-3718	3039	2	~	S-type pyocin T6SS effector
Aliivibrio finisterrensis_A	A37	NZ_SEZL01000074:679-3718	3039	2	~	S-type pyocin T6SS effector
Aliivibrio finisterrensis_A	A6	JARACR010000064.1:315-3657	3342	3	~	S-type pyocin T6SS effector
Aliivibrio finisterrensis_A	A9	NZ_SEZP01000068.1:679-3718	3039	2	~	S-type pyocin T6SS effector
Aliivibrio finisterrensis_A	A2	NZ_SEZS01000019.1:74180-78737	4557	6	~	sugar O-acetyltransferase
Aliivibrio finisterrensis_A	A33	NZ_SEZQ0100006.1:95937-100476	4539	6	~	sugar O-acetyltransferase
Aliivibrio finisterrensis_A	A4	NZ_SEZR01000005.1:117068-121607	4539	6	~	sugar O-acetyltransferase
Aliivibrio sifiae	CL6	LNRM01000025.1:716-4000	3284	3	~	superinfection exclusion B family protein
Aliivibrio sifiae	CL7	LNRN01000004.1:373802-380668	6866	8	~	superinfection exclusion B family protein, AAA family ATPase
Aliivibrio wodanis	BL6	JBXXRA010000001.1:1107251-1110172	2921	5	~	superinfection immunity protein, HAD hydrolase
Aliivibrio logei	SR45-2	JACJDQ010000116.1:86-2338	2252	4	~	Syd domain containing protein
Aliivibrio sifiae	NBRC 100501	MSCP01000002.1:910838-912436	1598	4	~	tautomerase family protein
Aliivibrio fischeri_B	emors.10.3	WOBU01000021.1:3288-8414	5126	7	~	TIGR04141 family sporadically distributed protein
Aliivibrio fischeri_B	emors.8.2	WOBE01000117.1:3287-8413	5126	7	~	TIGR04141 family sporadically distributed protein
Aliivibrio fischeri_B	emors.9.2	WOBB01000011.1:671735-676861	5126	7	~	TIGR04141 family sporadically distributed protein
Aliivibrio sifiae	2025O3Ga	JBXBSD010000008.1:33837-35129	1292	2	~	toll/interleukin-1 receptor domain-containing protein
Aliivibrio fischeri_B	SA1	JBUYRP010000001.1:39356-44151	4795	4	~	toll/interleukin-1 receptor domain-containing protein, ppGpp synthetase catalytic domain protein
Aliivibrio logei	1S159	MAJU01000022.1:96-1097	1001	2	~	transcriptional antiterminator Rof
Aliivibrio fischeri_B	ESF436_3	JAJVEE010000011.1:10000-15161	5161	7	~	Trypsin-like peptidase domain protein, Pas factor saposin fold domain containing protein
Aliivibrio fischeri_B	ESF436_4	JAJVEF010000005.1:193622-198783	5161	7	~	Trypsin-like peptidase domain protein, Pas factor saposin fold domain containing protein
Aliivibrio fischeri_B	ESF436_6	JAJVEH010000010.1:120625-125786	5161	7	~	Trypsin-like peptidase domain protein, Pas factor saposin fold domain containing protein
Aliivibrio logei	5S186	AJYJ02000124.1:13922-19959	6037	5	~	wobble nucleotide-excising tRNase
Aliivibrio sp.	S4MY1	NZ_JARACP010000035.1:542-8443	7901	6	~	YiiX/YebB-like N1pC/P60 family cysteine hydrolase

Aliivibrio sp.	S4MY3	NZ_JARACV010000039.1:542-8443	7901	6	~	YiiX/YebB-like N1pC/P60 family cysteine hydrolase
Aliivibrio sp.	S4MY4	NZ_JARACW010000036.1:26-7927	7901	6	~	YiiX/YebB-like N1pC/P60 family cysteine hydrolase
Aliivibrio sp.	S4TY1	NZ_JARACQ010000038.1:542-8443	7901	6	~	YiiX/YebB-like N1pC/P60 family cysteine hydrolase
Aliivibrio sp.	S4TY2	NZ_JARACX010000035.1:26-7927	7901	6	~	YiiX/YebB-like N1pC/P60 family cysteine hydrolase
Aliivibrio fischeri_A	HQB-91	NZ_JBHZSK010000020.1:10995-26069	15074	20		class I SAM-dependent methyltransferase, lysozyme inhibitor LprI family protein
Aliivibrio finisterrensis_A	A46	NZ_SEZK01000074:679-3781	3102	3		S-type pyocin T6SS effector
Aliivibrio "friggae"	SR10	JBXXRI010000001.1:1077442-1094186	16744	22		yqfB, nucleotidyltransferase family protein, ribB
Aliivibrio "friggae"	SR8	JBXXRG010000001.1:1076352-1093096	16744	21		yqfB, nucleotidyltransferase family protein, ribB
Aliivibrio "friggae"	SR9	NZ_CM170645.1:1077442-1094186	16744	21		yqfB, nucleotidyltransferase family protein, ribB

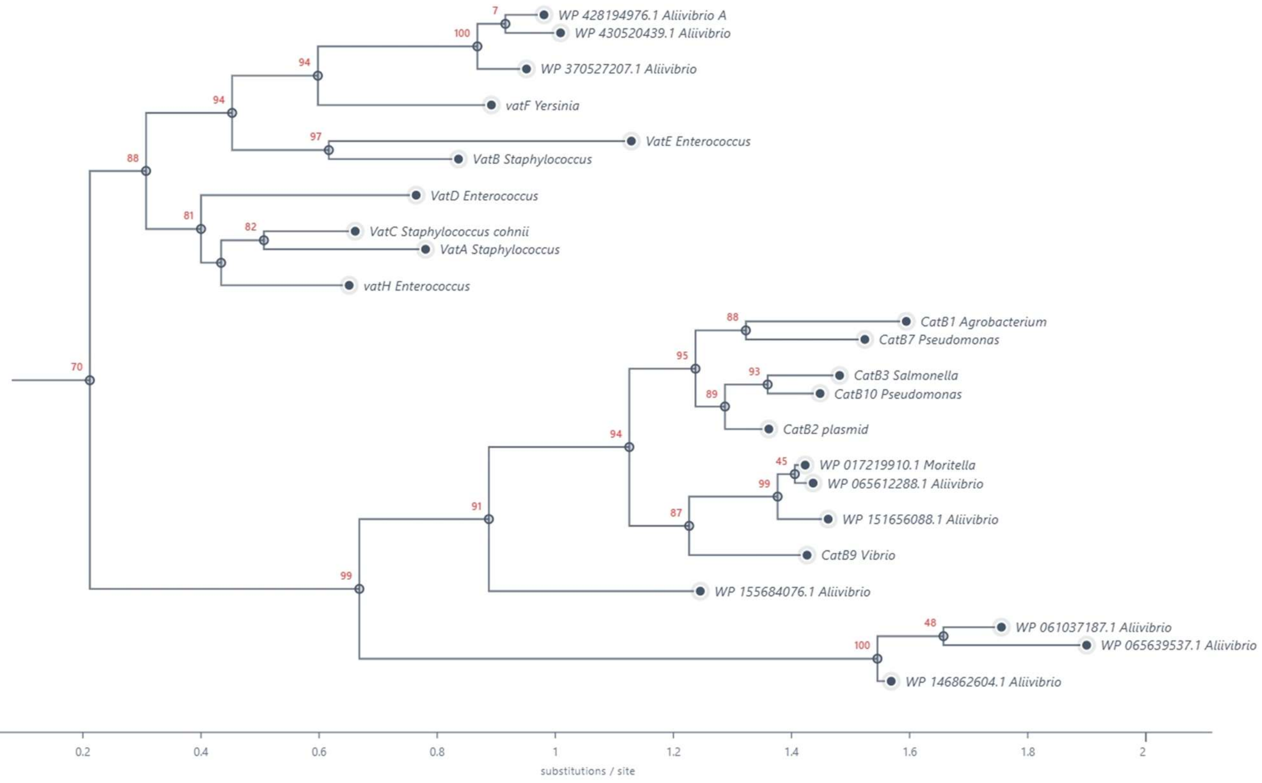

Figure S1. Comparison of CatB (chloramphenicol acetyltransferase type B) and Vat (virginiamycin/streptogramin acetyltransferases) homologous proteins from *Aliivibrio* and reference sequences from KEGG orthology. The outgroup sequence is GlmU (WP_017020791.1). The tree was generated using phylogeny.fr (MAFFT/FastTree).
